# Prevention of radiotherapy-induced pro-tumorigenic microenvironment by SFK inhibitors

**DOI:** 10.7150/thno.100970

**Published:** 2025-01-01

**Authors:** Yong June Choi, Myung Jun Kim, Young Joo Lee, Munkyung Choi, Wan Seob Shim, Miso Park, Yong-Chul Kim, Keon Wook Kang

**Affiliations:** 1 College of Pharmacy, Research Institute of Pharmaceutical Sciences and Natural Products Research Institute, Seoul National University, Seoul 08826, Republic of Korea.; 2 Department of Pharmacy, Kangwon National University, Chuncheon 24341, Republic of Korea.; 3 School of Life Sciences, Gwangju Institute of Science and Technology, Gwangju 61005, Republic of Korea.

**Keywords:** Radiotherapy, Pro-tumorigenic microenvironment, SRC family kinases, Macrophages

## Abstract

**Background:** Radiotherapy is a widely employed technique for eradication of tumor using high-energy beams, and has been applied to approximately 50% of all solid tumor patients. However, its non-specific, cell-killing property leads to inevitable damage to surrounding normal tissues. Recent findings suggest that radiotherapy-induced tissue damage contributes to the formation of a pro-tumorigenic microenvironment.

**Methods:** Here, we utilized two mouse strains and two organ-targeted radiotherapy models to uncover the mechanisms underlying the development of the radiotherapy-induced microenvironment.

**Results:** Radiotherapy-induced tissue damage stimulates infiltration of monocyte-derived macrophages and their differentiation into M2 macrophages, ultimately leading to fibrosis and the formation of a pro-tumorigenic microenvironment. Notably, SRC family kinases (SFKs) emerged as crucial factors in the formation of the radiotherapy-induced pro-tumorigenic microenvironment. SFKs activation in epithelial cells and fibroblasts was triggered by direct exposure to irradiation or M2 macrophage cytokines. Remarkably, the administration of SFK-targeted inhibitors reversed myofibroblast activation, effectively ameliorating fibrosis and the pro-tumorigenic microenvironment in radiated tissues. Further, combined administration of radiotherapy and SFK-targeted inhibitors significantly enhanced the survival of tumor-bearing mice.

**Conclusions:** Reshaping the tissue microenvironment by targeting SFKs is a potential strategy for preventing metastasis and recurrence following radiotherapy. The finding that clinically imperceptible damage can trigger a pro-tumorigenic microenvironment suggests the need for combining SFK-targeted inhibitors with radiotherapy.

## Introduction

Radiotherapy employing high-energy beams stands as a pivotal technique for direct elimination of cancer cells 1. It has been widely adopted as an anti-cancer tool, currently being utilized in over 50% of solid cancer cases 2. However, the considerable energy it delivers not only targets cancer cells but also impinges on surrounding healthy tissues 2. A notable side effect, radiotherapy-induced lung injury (RILI), is incurred by approximately 50% of patients undergoing thoracic radiotherapy 3, 4. Recent technological advancements, three-dimensional conformal radiotherapy (3D-CRT), intensity-modulated radiotherapy (IMRT), and stereotactic body radiotherapy (SBRT) have emerged, promising more precise applications 5. Despite these innovations, complete avoidance of side effects on normal tissues remains unrealistic 4, 6. Notably too, recent studies indicate that RILI has the potential to transform the lung into a pro-tumorigenic microenvironment 7-9. Clinical observations have shown that radiotherapy may lead to increased rates of local recurrence or mortality in certain cases 10-12. Given this, the prevention and management of RILI are critical not only for restoring lung function but also for reducing risks of radiotherapy-induced metastasis, recurrence, and related mortality 2, 7, 10-13. Although glucocorticoids are commonly used for treating RILI, controlled studies evaluating their effectiveness in humans remain limited 14, 15. Furthermore, glucocorticoid therapy is generally considered ineffective for patients with chronic conditions, such as radiation-induced fibrosis 15. This limitation emphasizes the urgent need for the development of more targeted and effective therapeutic options.

SRC family kinases (SFKs), a family of non-receptor protein tyrosine kinases, play a crucial role in signal transduction of various cellular processes in cancer cells 16, 17. The roles and mechanisms of SFKs in tumorigenesis have been extensively investigated, and SFKs recently have been recognized as potential targets for anti-cancer therapy 17-19. Nevertheless, research on the functions of SFKs and their targeting in other cell types within the tumor microenvironment remains limited. Recent studies have begun to uncover the role of SFKs in fibrosis and myofibroblast activation 20, 21, with findings indicating that Fyn is involved in liver fibrosis and Fgr in pulmonary fibrosis 22, 23. However, the roles of SFKs, particularly SRC and YES1, in fibrosis-driven tumor microenvironment modification are still not well understood.

Inflammation after tissue damage is a key trigger of tissue regeneration and fibrosis. Acute damage in parenchymal cells not only induces inflammation but also polarizes it by recruiting diverse types of innate and adaptive immune cells 24. A recent study has shown that radiation-induced neutrophil infiltration into the lung contributes to a pro-tumorigenic microenvironment 7. Specifically, neutrophil granulation promotes cancer cell engraftment through activation of Notch signaling in lung epithelial cells 7. However, the functions of other immune cells and the mechanisms contributing to increase in radiotherapy-induced metastasis remain incompletely understood.

This study employed mouse models to investigate the mechanisms underlying the formation of a radiotherapy-induced pro-tumorigenic microenvironment. The findings confirm that increased macrophage infiltration and M2 differentiation are required for fibrogenesis in irradiated lung tissues. Furthermore, SFKs emerged as a crucial factor associated with radiotherapy-induced fibrosis. Two SFK-targeted inhibitors effectively reversed myofibroblast activation induced by TGF-β or direct irradiation. In animal experiments, these inhibitors also reversed lung cancer cell engraftment by inhibiting fibrosis. Based on the findings, this study proposes SFKs as a novel target for prevention of early-stage lung fibrosis induced by radiotherapy.

## Results

### Formation of pro-tumorigenic lung microenvironment following radiotherapy-induced early-stage lung fibrosis

**Figure 1:**
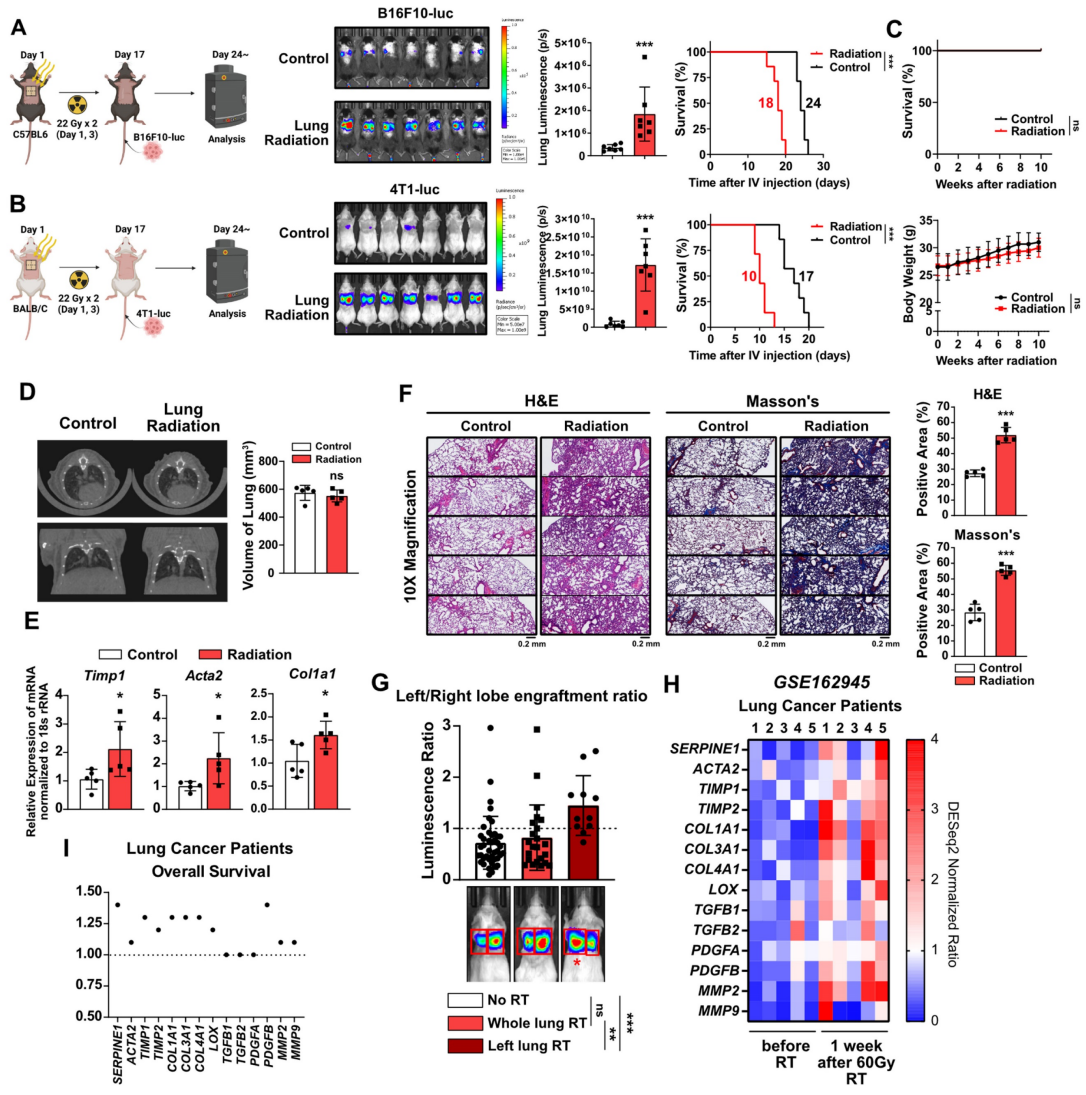
** Early-stage lung fibrosis caused by radiotherapy creates pro-tumorigenic lung microenvironment.** (**A, B**) The assessment of lung engraftment of cancer cells and mouse survival. Cancer cells were injected into the tail vein, 2 weeks after irradiation (22 Gy×2). The median survival time is shown on the graph. *n* = 7/group. (**C**) The assessment of survival rate and body weight changes in mice after irradiation alone. Survival rate and body weight changes were monitored for 10 weeks. *n* = 7/group. C57BL6 mice were used. (**D**) CT scans to detect lung injury, 4 weeks after irradiation. *n* = 5/group. Functional lung volume was calculated using micro-CT software. C57BL6 mice were used. (**E**) mRNA expressions of fibrosis markers (*Timp1*, *Acta2* and *Col1a1*) in mouse lung tissues, 4 weeks after irradiation. (**F**) Detection of alveolar collapse (H&E staining) and collagen accumulation (Masson's trichrome staining, Blue) in mouse lung tissues, 4 weeks after irradiation. Whole lung cross-section data are shown in Figure S10. *n* = 5/group. (**G**) Comparison of cancer cell engraftment in the left and right lung lobes after targeted radiotherapy to the left lung (44 Gy×1, *n* = 11). BALB/c mice with 4T1-luc cells were used. Graphs for the no RT group and the whole lung RT group were generated using the data from Figure 1 to 7. (**H**) Comparison gene expression of fibrosis markers in lung tissues before and 1 week after 60 Gy RT treatment in lung cancer patients. The NCBI public dataset, GSE162945, was used. (**I**) Correlation between each fibrosis marker and overall survival in lung cancer patients. The risk of survival was expressed as hazard ratio. The GEPIA public database was used. All data were presented as mean ± SD. Statistical significance of the differences was determined by two-tailed Student t-test or one-way ANOVA followed by the Tukey's test.

To investigate the potential induction of early-stage lung fibrosis and the shaping of a pro-tumorigenic lung microenvironment following chest irradiation, mouse models were established. The chest of mice irradiated within a specified region (1.5 cm × 1 cm), mimicking the dosage used in clinical stereotactic body radiotherapy 25 (**Figure S1**). For experimental purposes, the irradiation was applied to a broader anatomical region than is typically the case clinically. This led to increased lung engraftment of metastatic cancer cells in two different mouse strains, resulting in reduced survival rates (**Figure 1A and 1B**). However, radiation alone did not cause weight loss or mortality during the same period (**Figure 1C**). To determine if the radiation-induced engraftment of metastatic cancer cells in the lungs was due to lung fibrosis, micro-computed tomography (CT) scans were performed four weeks after irradiation. Although there was no apparent lung damage in the CT scans, upregulation of fibrosis-associated genes was detected (**Figure 1D and 1E**). Histological analyses using H&E and Masson's Trichrome staining revealed augmented alveolar collapse and collagen accumulation, confirming the formation of early-stage lung fibrosis (**Figure 1F**). These findings indicate that even mild fibrosis caused by chest irradiation may contribute to the formation of a pro-tumorigenic lung microenvironment.

Further investigations utilized a left lung-targeted radiation model (0.2 cm × 0.2 cm) (**Figure S1**). Because the right lung containing four lobes has a larger overall volume compared with the left lung containing just one lobe 26, relatively higher luminescence of cancer cells was detected in the right lungs of sham and whole lungs irradiation groups (**Figure 1G**). However, increased left lung engraftment (left/right lung luminescence ratio) was found in the left lung-targeted radiation model, confirming the formation of a radiation-specific pro-tumorigenic microenvironment (**Figure 1G**). Analysis of lung cancer patients from a public dataset confirmed elevated fibrosis markers in lung tissues 1 week after 60 Gy SBRT treatment (**Figure 1H**), and the majority of the fibrosis markers were verified as being associated with negative impacts on patient survival (**Figure 1I**). These results demonstrate that radiotherapy can induce clinically undetectable, early-stage lung fibrosis, which may create a pro-tumorigenic lung microenvironment.

### Induction of lung fibrosis by radiotherapy via macrophage infiltration and M2 differentiation

**Figure 2:**
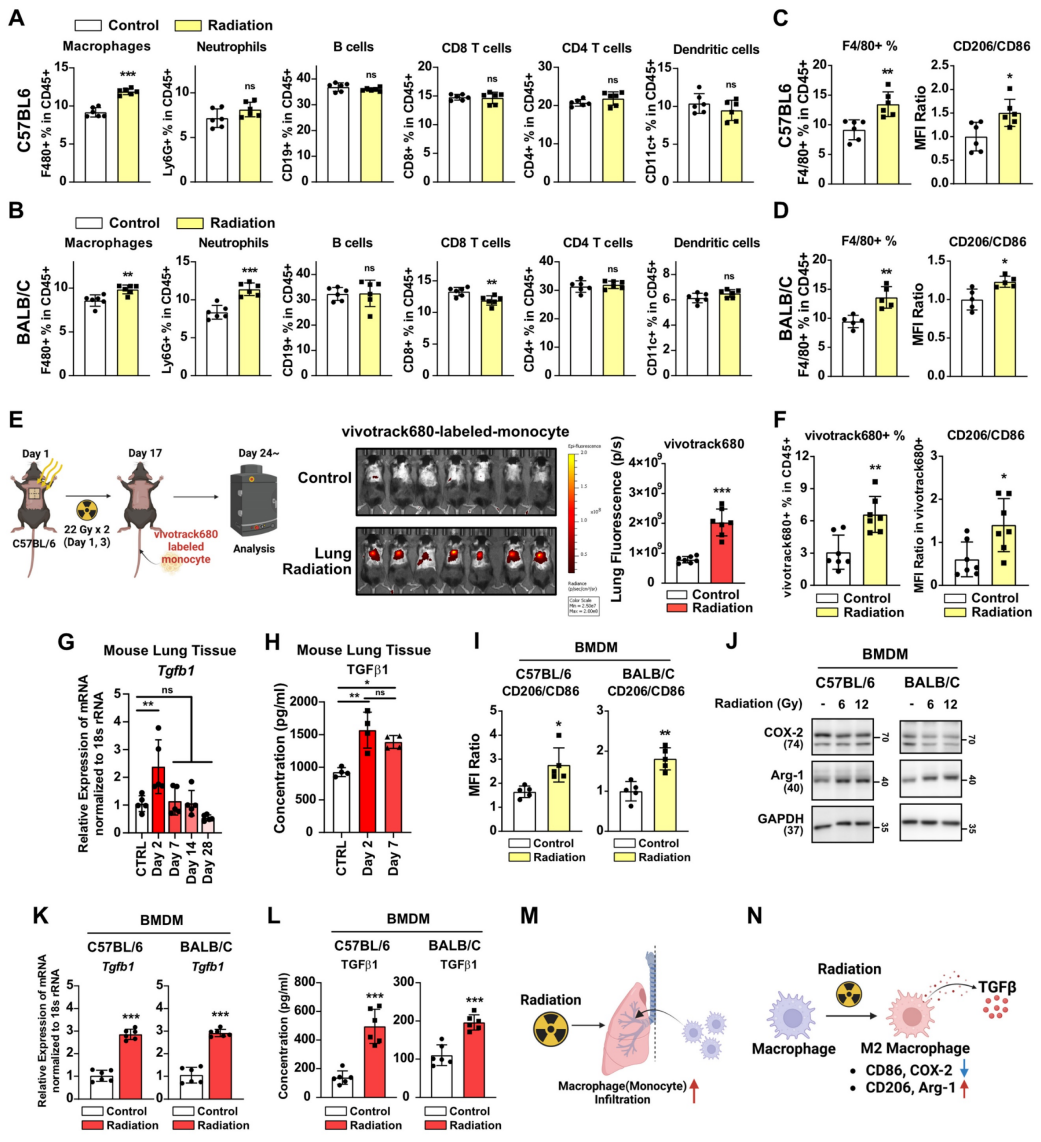
** Radiotherapy induces macrophage infiltration and M2 differentiation.** (**A, B**) Changes in immune cells infiltration in lung tissues. Lung tissues were collected 3 days after radiation. *n* = 6/group. (**C, D**) Changes in macrophage infiltration and M2 differentiation in lung tissues, 2 weeks after radiation (22 Gy×2). *n* = 5 or 6/group. The gating strategy and the percentages of M1 and M2 macrophages are shown in Figure S2. (**E**) Fluorescence of vivotrack680-labeled monocyte and monocyte-derived macrophages. *n* = 7/group. (**F**) Infiltration and M2 differentiation of vivotrack680-labeled cells in lung tissues. (**G**) Identification of Tgf beta 1 mRNA levels in lung tissue after chest irradiation (22 Gy×2). Mice were sacrificed 2, 7, 14, and 28 days after irradiation. *n* = 5/group. C57BL6 mice were used. (**H**) Identification of secreted TGF beta 1 protein levels in lung tissue after chest irradiation. Mice were sacrificed 2 and 7 days after irradiation. *n* = 4/group. C57BL6 mice were used. (**I**) M2 differentiation of BMDMs 24 h after direct radiation exposure. (**J**) Western blot analyses for M1 and M2 markers in BMDMs 24 h after direct radiation exposure. (**K**) Measurement of Tgf beta 1 mRNA levels 24 h after direct irradiation of BMDM (12 Gy). (**L**) Measurement of TGF beta 1 protein secretion 24 h after direct irradiation of BMDM (12 Gy). (**M, N**) Schematic illustrations representing Figure 2 results. All data were presented as mean ± SD. Statistical significance of the differences was determined by two-tailed Student t-test or one-way ANOVA followed by the Tukey's test. All western blot analyses in this study were repeated three times independently.

We investigated the mechanisms underlying radiotherapy-induced lung fibrosis, focusing on the impact of radiation on immune cells as the key contributor to tissue damage 27. Chest irradiation led to increased macrophage infiltration in the lungs of mice, 3 days after irradiation, as was observed in both strains (**Figure 2A and 2B, Figure S2**). Enhanced neutrophil infiltration was evident only in BALB/c mice, as previously reported 7 (**Figure 2B**). Importantly, macrophage infiltration persisted two weeks post-irradiation, accompanied by increased M2 differentiation (CD206/CD86 MFI ratio) (**Figure 2C and 2D**). As both forms of macrophage, tissue-resident and monocyte-derived, coexist within lung tissues 28, 29, additional lung macrophage markers were analyzed to identify the specific macrophage type(s) affected by radiation (**Figure S3A**). It was found that chest irradiation increased the infiltration of monocytes and monocyte-derived macrophages into the lungs, but that there was no change in the tissue-resident macrophages (**Figure S3B**). Moreover, increased M2 differentiation was observed only in the monocyte-derived macrophages (**Figure S3C and S3D**). To further validate the increased infiltration of monocytes and monocyte-derived macrophages into lung tissues by radiation, vivotrack680-labeled-monocytes and -monocyte-derived macrophages were injected into the mice following chest irradiation. Three days after the cell injection, *in vivo* tracking of labeled monocytes and monocyte-derived macrophages confirmed the increased infiltration and M2 differentiation in the irradiated group (**Figure 2E and 2F**). TGF-β, a key cytokine secreted by M2 macrophages, plays a major role in mediating fibrosis 30. We investigated radiation-induced TGF-β production and secretion in mouse lung tissue. Our results showed that the production and secretion of TGF-β1 increased in the early-stages following radiation exposure (**Figure 2G and 2H**). *In vitro* experiments further corroborated that monocyte-derived macrophages had directly responded to radiation, leading to increased M2 differentiation (**Figure 2I and 2J**). Additionally, direct radiation exposure was shown to increase both TGF-β1 production and secretion in macrophages (**Figure 2K and 2L**). Overall, it could be established lung irradiation induces infiltration of monocyte-derived macrophages into lung tissues, thereby enhancing M2 differentiation both directly and indirectly (**Figure 2M and 2N**).

**Figure 3:**
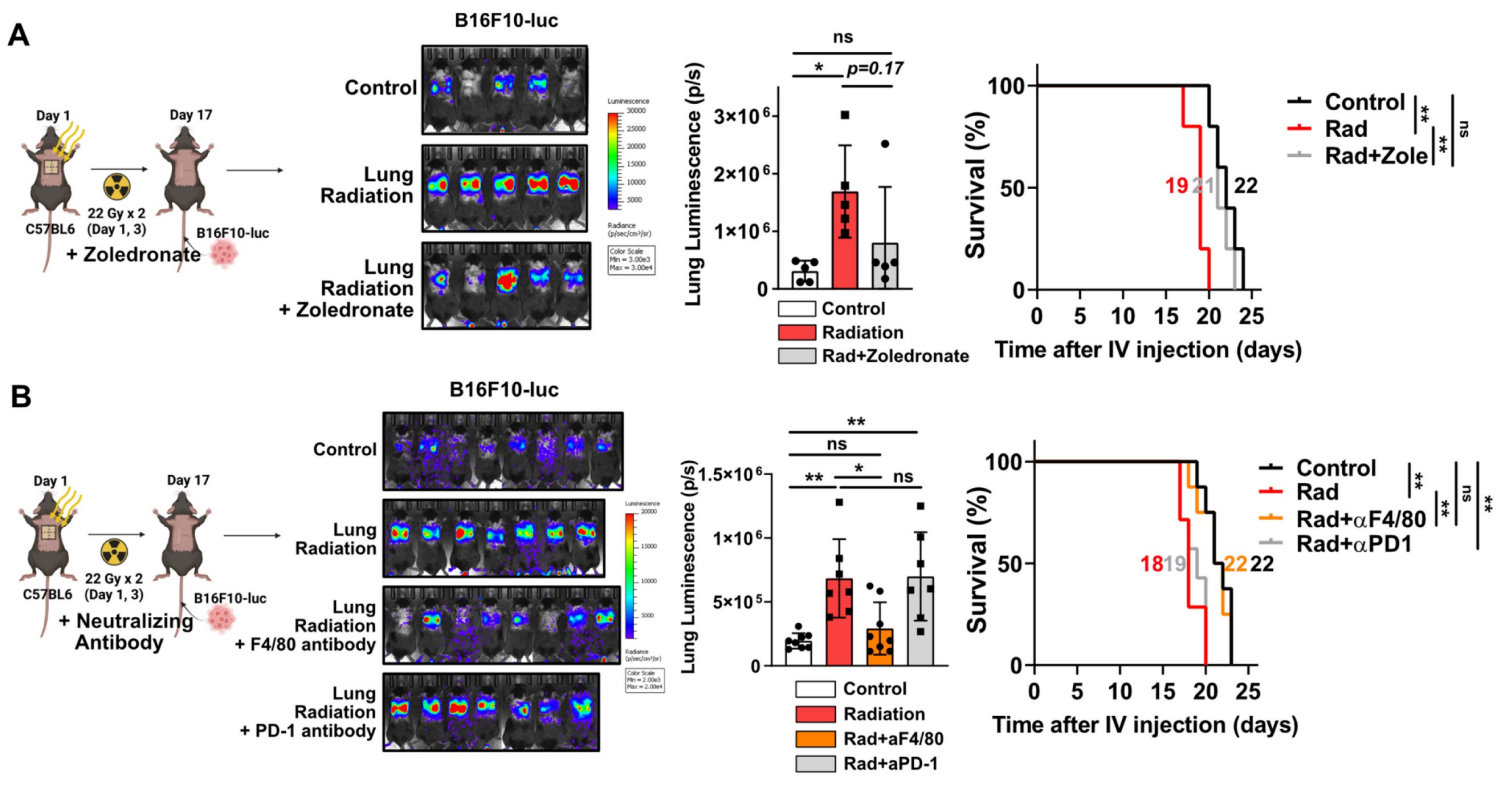
** Macrophage infiltration and M2 differentiation contribute to the pro-tumorigenic lung microenvironment.** (**A**) The assessment of lung engraftment of cancer cells and mouse survival using chemical-induced macrophage depletion (zoledronate, 0.2 mg/kg, per week, IP). Cancer cells were injected into the tail vein, 2 weeks after irradiation (22 Gy×2). The median survival time is shown on the graph. *n* = 5/group. (**B**) The assessment of lung engraftment of cancer cells and mouse survival using macrophage depletion antibody (F4/80 antibody, 10 mg/kg, twice a week, IP) or PD-1 antibody (10 mg/kg, twice a week, IP). Cancer cells were injected into the tail vein, 2 weeks after irradiation (22 Gy×2). *n* = 7 or 8/group. All data were presented as mean ± SD. Statistical significance of the differences was determined by one-way ANOVA followed by the Tukey's test.

Next, we investigated whether increased macrophage infiltration caused by radiation plays a pivotal role in pro-tumorigenic microenvironment formation. After chest irradiation, the fibrotic areas exhibited infiltrated F4/80-positive cells (macrophages) relative to the non-fibrotic area (**Figure S4A**). Furthermore, TGF-β expression was found to coincide with areas of F4/80-positive cell infiltration, supporting its involvement in fibrosis development (**Figure S4B**). Bisphosphonate, commonly used to treat osteoporosis, can induce macrophage death and reduce M2 differentiation while increasing M1 differentiation 31, 32. Administrations with a zoledronate or F4/80 neutralizing antibody, targeting macrophages, successfully reduced cancer cell engraftment and improved mice survival (**Figure 3A and 3B**). However, co-administration of PD-1 antibody failed to affect either cancer cell engraftment or survival (**Figure 3B**). These results imply that formation of a pro-tumorigenic lung microenvironment induced by radiation depends on the functions of macrophages rather than T cells.

To determine how radiation induces macrophage recruitment, we performed a cytokine array analysis. A cytokine array analysis further identified elevated levels of P-selectin, TSP-1, CXCL7, CXCL14, and CXCL2 in irradiated lung tissues, all of which are known to be associated with increased infiltration of monocyte-derived macrophages 33-35 (**Figure S5**). These findings confirm that radiation-induced increases in specific cytokines may play a role in driving macrophage infiltration.

### SFK-dependent myofibroblast activation by TGF-β

**Figure 4:**
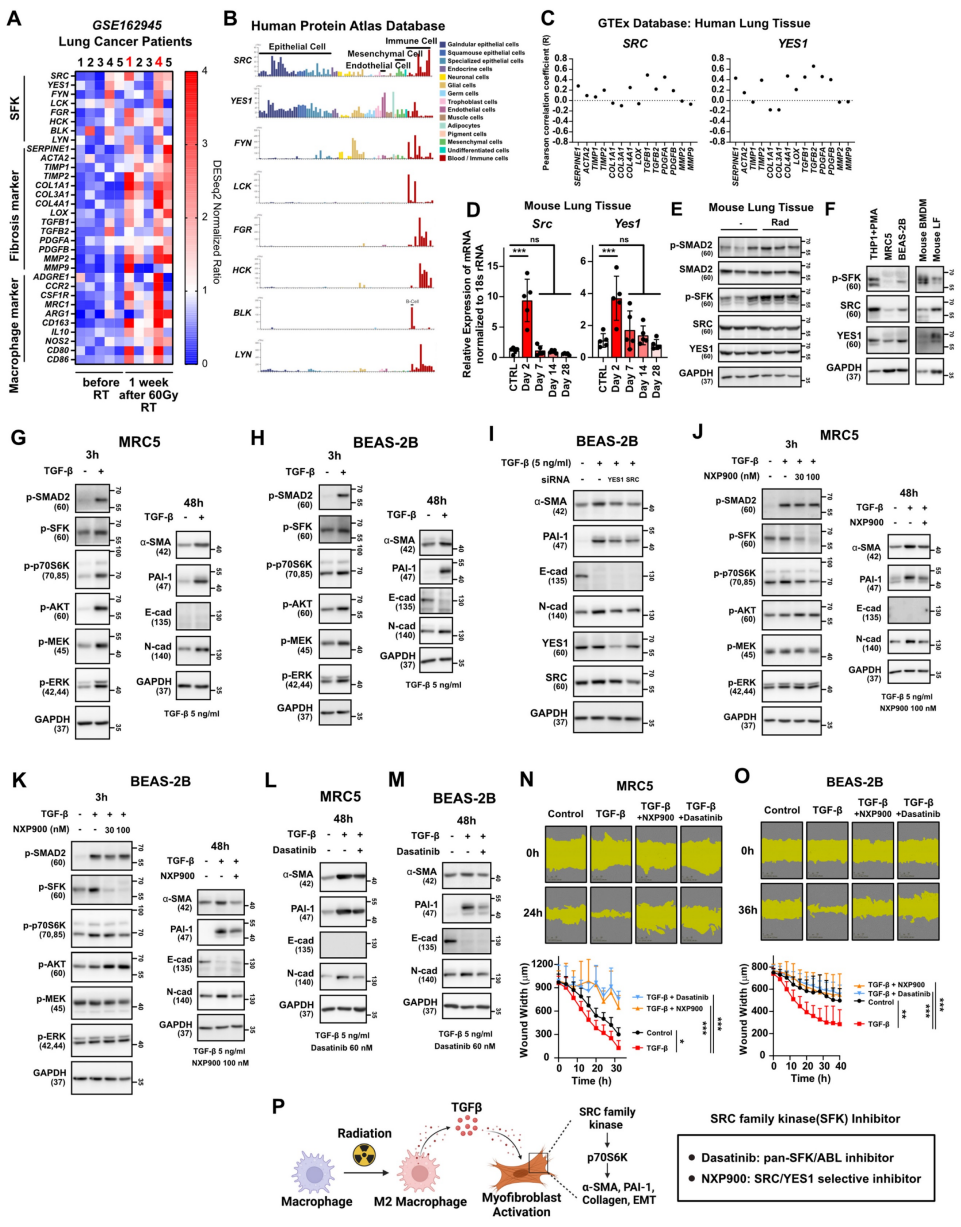
** TGF-β-induced myofibroblast activation mediates SFKs activation in the lung microenvironment.** (**A**) Comparison gene expression of SFKs, fibrosis, macrophage infiltration markers in lung tissues before and 1 week after 60 Gy SBRT treatment in lung cancer patients. The NCBI public dataset, GSE162945, was used. (**B**) Cell-type dependent gene expression of SFKs. The Human Protein Atlas database was used. (**C**) Correlation analyses between each fibrosis marker and SRC or YES1. The GEPIA database was used. (**D**) Identification of Src and Yes1 mRNA levels in lung tissue after chest irradiation. Mice were sacrificed 2, 7, 14, and 28 days after irradiation. *n* = 5/group. (**E**) Identification of SFKs and SMAD2 activity in lung tissue after chest irradiation. Mice were sacrificed 3 days after irradiation. *n* = 3/group. (**F**) Expression and activity of SFKs protein in mouse macrophages and lung fibroblasts and their relevant human cell lines. (**G, H**) Activation of myofibroblasts by TGF-β treatment and its mechanism. Total protein levels are shown in Figure S11. (**I**) Reversal of TGF-β-induced myofibroblast activation by specific knockdown of SRC or YES1. (**J, K**) Reversal of TGF-β-induced myofibroblast activation and its mechanism by NXP900. Total protein levels are shown in Figure S11. (**L, M**) Reversal of TGF-β-induced myofibroblast activation by dasatinib. (**N, O**) Reversal of TGF-β-induced wound healing phenotype by SFK-inhibitors. (**P**) Schematic illustration representing Figure 4 results. All data were presented as mean ± SD. Statistical significance of the differences was determined by two-tailed Student t-test or one-way ANOVA followed by the Tukey's test. All western blot analyses in this study were repeated three times independently.

We next investigated how macrophage infiltration and M2 differentiation in lung tissues contribute to progression of lung fibrosis. Analysis of public data from lung cancer patients treated with 60 Gy of SBRT revealed a significant increase in markers indicating macrophage infiltration and fibrosis, particularly in patients 1 and 4 (**Figure 4A**). Notably, an elevation in the expression of SFKs was also observed in these patients (**Figure 4A**), supporting the potential association between SFKs and radiotherapy-induced lung fibrosis. SFKs, a group of eight protein kinases known for their role in cancer growth and metastasis 16, recently have been linked to fibrosis processes 20, 22, 23. Examining SFKs expression across various cell types in lung tissues revealed predominant expression in immune cells, with SRC and YES1 exhibiting especially high levels in epithelial, mesenchymal, and endothelial cells, all of which play crucial roles in fibrosis progression (**Figure 4B**). Importantly, SRC and YES1 expression were found to be correlated with the majority of fibrosis markers in human lung tissues (**Figure 4C**). When we assessed the expression of SRC and YES1 in irradiated mouse lung tissue, radiation led to increased mRNA expression of both kinases (**Figure 4D**). Additionally, SFKs activation in mouse lung tissues was heightened by irradiation (**Figure 4E**). Expression of SRC or YES1 also was validated in primary cells and their surrogate human cell lines (**Figure 4F**). Accordingly, to investigate possible TGF-β mediation of fibrosis via SFKs activation, we assessed TGF-β-stimulated downstream fibrosis signaling in lung fibroblasts and epithelial cells. TGF-β induced SFKs activation in both MRC5 and BEAS-2B cells, resulting in increases in myofibroblast activation markers such as α-SMA, PAI-1, and N-cadherin, along with a decrease in E-cadherin (**Figure 4G and 4H**). Selective knockdown of SRC and YES1 reversed the increase in myofibroblast activation markers caused by TGF-β (**Figure 4I**). Taking all of these results together, TGF-β, an M2 cytokine, can be said to mediate fibrosis via SFKs activation in lung fibroblasts and epithelial cells.

To investigate the potential preventive effects of SFK-targeted inhibitors on myofibroblast activation induced by TGF-β, two SFK-inhibitors were employed: dasatinib, an FDA-approved SFK-targeted inhibitor used for chronic myeloid leukemia 36, and NXP900, a novel SRC/YES1-targeted inhibitor with superior selectivity that currently is in clinical trials 37, 38. NXP900 treatment effectively reduced SFKs activation induced by TGF-β in both MRC5 and BEAS-2B cells (**Figure 4J and 4K left**), and, furthermore, reversed the myofibroblast activation induced by TGF-β (**Figure 4J and 4K right**). Notably, NXP900 did not affect the canonical TGF-β pathway, as evidenced by the unchanged SMAD2 phosphorylation, but did impact the non-canonical pathway through decreased p70S6K activity (**Figure 4J and 4K left**). Additionally, dasatinib also reversed the myofibroblast activation induced by TGF-β (**Figure 4L and 4M**). Both NXP900 and dasatinib hindered TGF-β-induced wound healing in MRC5 and BEAS-2B cells, further confirming the functional role of SFKs in myofibroblast activation (**Figure 4N and 4O**). In summary, SFK-targeted inhibitors can effectively prevent myofibroblast activation mediated by M2 cytokine TGF-β (**Figure 4P**).

Given that the highest expression and activity of SFKs was in macrophages (**Figure 4B and 4F**), the direct effects of SFK-targeted inhibitor on macrophage differentiation were further examined. To assess this, macrophages were treated with NXP900 before and after differentiation. Mouse bone marrow cells were differentiated into macrophages for 6 days and then treated with NXP900 for 24 hours (**Figure S6A**). NXP900 effectively inhibited SFK activity in these macrophages but did not impact cell viability or M1 and M2 differentiation (**Figure S6B-S6D**). In a separate experiment, mouse bone marrow cells were treated with NXP900 continuously during differentiation (**Figure S6E**). NXP900 treatment did not alter the overall macrophage count but did result in a slight increase in M2 differentiation (**Figure S6F**). Additionally, THP-1 cells, a human monocyte cell line, were differentiated into macrophages via PMA pretreatment and then exposed to NXP900. NXP900 had no significant effect on M1 and M2 differentiation in human monocyte cells (**Figure S6G and S6H**). To examine the activity of SFK during M2 differentiation, mouse macrophages were differentiated into M2 macrophages using TGF-β and IL-4, respectively. SFK activity remained unchanged or was slightly suppressed during M2 differentiation induced by either TGF-β or IL-4 (**Figure S6I and S6J**). These results confirm that SFK-targeted inhibitor may exert limited effects on macrophage differentiation.

### Inhibitory effect of SFK-targeted inhibitors on direct irradiation-induced myofibroblast activation

**Figure 5:**
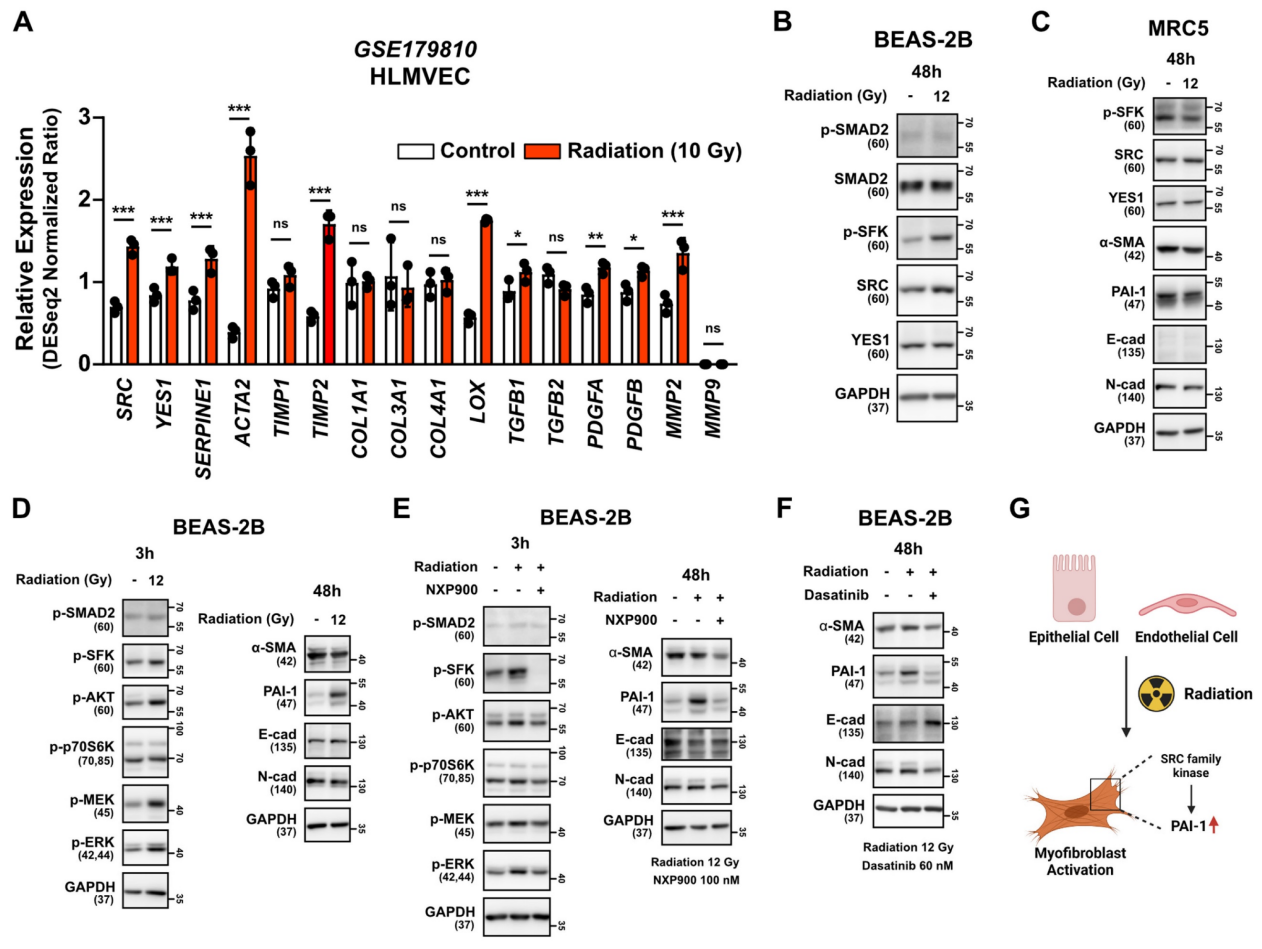
** SFK-targeted inhibitors suppress direct radiation-induced myofibroblast activation.** (**A**) Comparison gene expression of SRC, YES1 and fibrosis markers in HLMVEC, 24 h after 10 Gy direct irradiation. The NCBI public dataset, GSE179810, was used. (**B**) SFKs expression and activation by direct irradiation in BEAS-2B cells. (**C**) Myofibroblast activation by direct irradiation in MRC5 cells. (**D**) Myofibroblast activation and its mechanism following direct irradiation in BEAS-2B cells. Total protein levels are shown in Figure S11. (**E, F**) Reversal of direct radiation-induced myofibroblast activation by SFK-inhibitors. Total protein levels are shown in Figure S11. (**G**) Schematic illustration representing these results. All western blot analyses in this study were repeated three times independently.

To determine if myofibroblast activation is directly induced by irradiation, we examined the National Center for Biotechnology Information (NCBI) public datasets on the human lung endothelial cell line HLMVEC. Increased expression of SRC, YES1 and fibrosis markers was observed 24 hours after direct irradiation. (**Figure 5A**). Additionally, direct irradiation to the BEAS-2B cells led to increased SRC protein expression and SFKs activation, but not SMAD2 activation, as is consistent with the public data (**Figure 5B**). Interestingly, direct irradiation did not increase phosphorylated SFKs or markers for myofibroblast activation in a human lung fibroblast cell line, MRC5 (**Figure 5C**), but it did induce PAI-1 expression in the BEAS-2B cells (**Figure 5D**). Subsequently, we investigated whether SFK-targeted inhibitors could reverse myofibroblast activation induced by direct irradiation. Both SFK-targeted inhibitors effectively reversed radiation-induced myofibroblast activation (**Figure 5E and 5F**). These results suggest that radiation has the potential to directly induce myofibroblast activation in lung epithelial cells and, also, that treatment of SFK-targeted inhibitors could prevent this process (**Figure 5G**).

### Inhibition of radiotherapy-induced lung fibrosis by SFK-targeted inhibitors and subsequent prevention of pro-tumorigenic lung microenvironment

**Figure 6:**
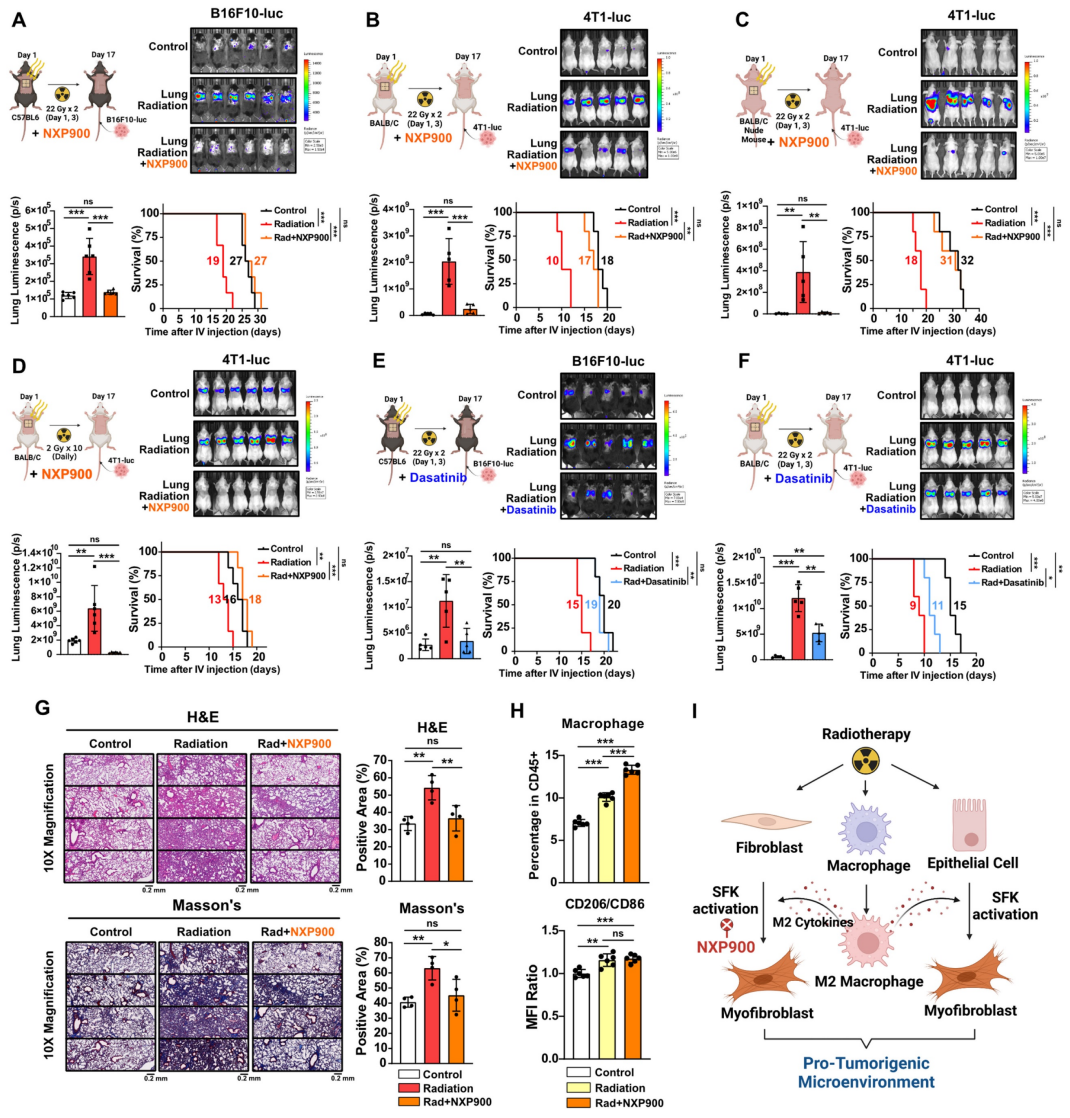
** SFK-targeted inhibitors prevent pro-tumorigenic lung microenvironment through alleviating lung fibrosis.** (**A-C**) The assessment of lung engraftment of cancer cells and mouse survival. Cancer cells were injected into the tail vein, 2 weeks after irradiation (22 Gy×2, day 1, 3). NXP900 (60 mg/kg, daily, PO) was administered from day 1 to 16. The median survival time is shown on the graph. *n* = 5 or 6/group. (**D**) The assessment of lung engraftment and mouse survival. Mice received radiation at a dose of 2 Gy per day for a total of 10 days. *n* = 6/group. (**E, F**) The assessment of lung engraftment and mouse survival. Cancer cells were injected into the tail vein, 2 weeks after irradiation. Dasatinib (30 mg/kg, daily, PO) was administered from day 1 to 16. The median survival time is shown on the graph. *n* = 5/group. (**G**) Detection of alveolar collapse (H&E) and collagen accumulation (Masson's trichrome, Blue) in mouse lung tissues, 4 weeks after irradiation. NXP900 (60 mg/kg, daily, PO) was administered from day 1 to 28. *n* = 4/group. Whole lung cross-section data are shown in Figure S10. (**H**) Macrophage infiltration and M2 differentiation in the lungs following irradiation and NXP900 administration. Mice were sacrificed 2 weeks after irradiation (22 Gy×2, day 1, 3). NXP900 (60 mg/kg, daily, PO) was administered from day 1 to 16. (**I**) Schematic illustration representing this study. All data were presented as mean ± SD. Statistical significance of the differences was determined by one-way ANOVA followed by the Tukey's test.

We then investigated the potential of SFK-targeted inhibitors to prevent radiotherapy-induced lung fibrosis and subsequent development of a pro-tumorigenic lung microenvironment. Mice were subjected to chest irradiation coupled with daily oral administration of NXP900 for 16 days, and metastatic cancer cells (B16F10-luc and 4T1-luc) were injected through the tail vein. NXP900 administration reversed cancer cell engraftment, leading to improved mice survival (**Figure 6A and 6B**). Further confirming the negligible role of T cells in establishing a pro-tumorigenic lung microenvironment post-irradiation (**Figure 2A and 3B**), T cell-deficient BALB/c-nude mice still exhibited increased cancer cell engraftment after chest irradiation, which was effectively reversed by NXP900 administration (**Figure 6C**). To assess whether a pro-tumorigenic lung microenvironment could be induced by repetitive low-dose radiotherapy, mice were subjected to daily chest irradiation of 2 Gy for 10 days. This repetitive low-dose radiotherapy also resulted in increased engraftment of cancer cells, which was completely reversed by NXP900 administration (**Figure 6D**). Importantly, administration of NXP900 without radiation did not impact cancer cell engraftment, confirming that the reversal of cancer cell engraftment by NXP900 was not a direct inhibitory effect on cancer cells due to the remaining drug in the blood (**Figure S7**). Furthermore, dasatinib showed the same tendency with NXP900, reversing cancer cell engraftment and improving mice survival in both strains (**Figure 6E and 6F**). To confirm that NXP900 prevents lung fibrosis induced by radiation, C57BL/6 mice after chest irradiation were orally administered NXP900 for 4 weeks. Histological analyses revealed that NXP900 effectively inhibited both alveolar collapse and collagen accumulation by radiation (**Figure 6G**). Although NXP900 treatment increased the infiltration of macrophages into the lungs, it did not affect the extent of M2 differentiation (**Figure 6H**). Therefore, it could be inferred that the SFK-targeted inhibitor suppresses fibrosis and pro-tumorigenic microenvironment by targeting myofibroblast activation rather than macrophage modulation. In summary, SFK-targeted inhibitors have the potential to prevent a pro-tumorigenic lung microenvironment by inhibiting lung fibrosis that arises through either the direct-irradiation or indirect-macrophage pathway (**Figure 6I**).

### Combination therapy with SFK-targeted inhibitors improves radiotherapy-induced anti-cancer effects

**Figure 7:**
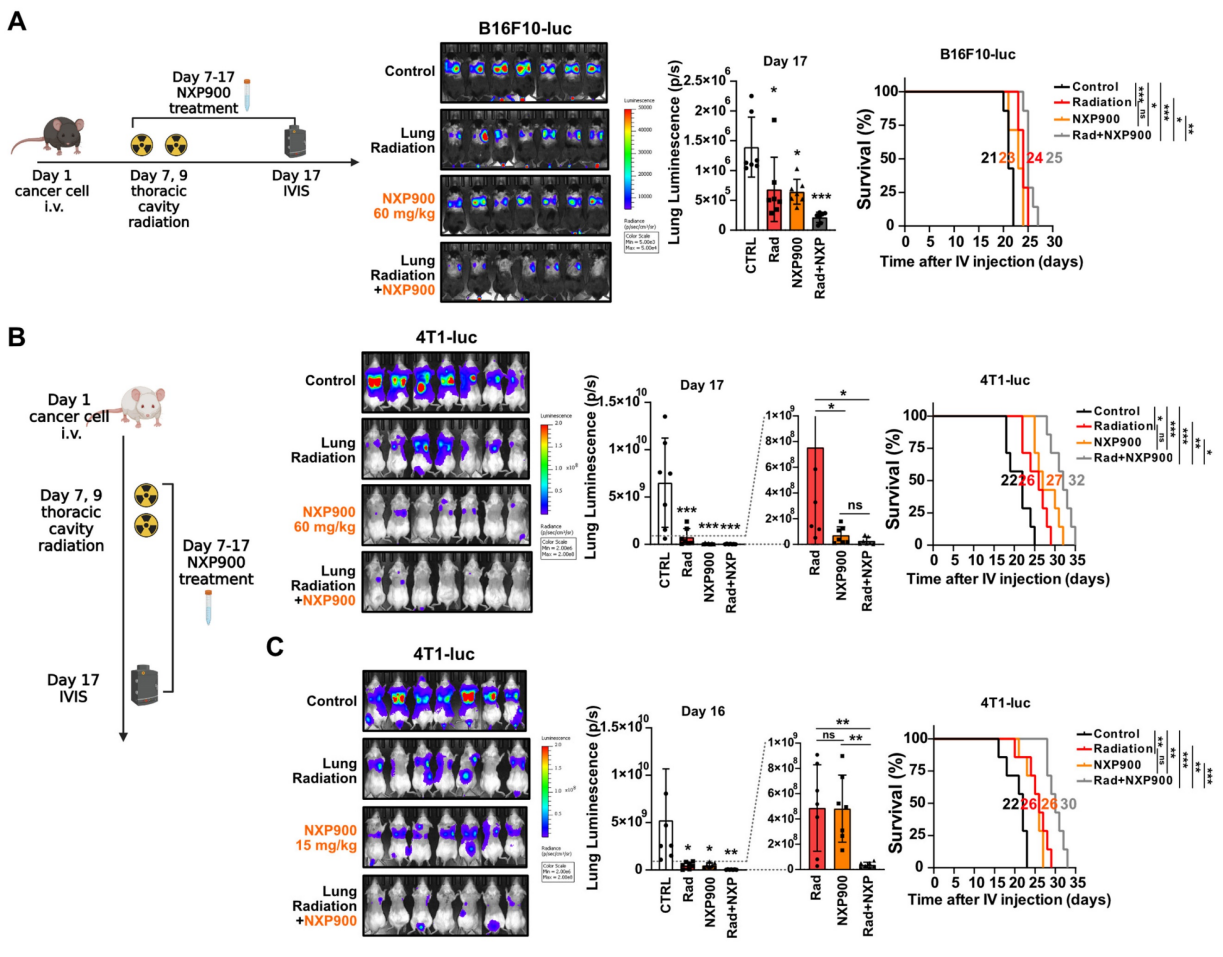
** Combination of NXP900 with radiotherapy demonstrate synergistic anti-cancer efficacy.** (**A**) The anti-cancer efficacy of combination treatment using radiotherapy (22 Gy×2, day 7, 9) and NXP900 (60 mg/kg, daily, PO, day 7-17). B16F10-luc cells were injected into C57BL/6 mice (day 1) and treatment started on day 7. The median survival time is shown on the graph. *n* = 7/group. (**B**) The anti-cancer efficacy of combination treatment using radiotherapy (22 Gy×2, day 7, 9) and high dose NXP900 (60 mg/kg, daily, PO, day 7-17). 4T1-luc cells were injected into BALB/c mice (day 1) and treatment started on day 7. The median survival time is shown on the graph. *n* = 7/group. (**C**) The anti-cancer efficacy of combination treatment using radiotherapy (22 Gy×2, day 7, 9) and low dose NXP900 (15 mg/kg, daily, PO, day 7-17). *n* = 7/group. All data were presented as mean ± SD. Statistical significance of the differences was determined by two-tailed Student t-test or one-way ANOVA followed by the Tukey's test.

Given the multifaceted roles of SFK in regulating pathways associated with cell proliferation and metastasis 39, we assessed whether SFK-targeted inhibitors directly inhibit proliferation and migration of cancer cells. Both SFK-targeted inhibitors were able to directly suppress proliferation and migration of mouse cancer cells (**Figure S8A-S8D**). To elucidate the mechanisms by which SFK-targeted inhibitors suppress proliferation and migration of cancer cells, various downstream signaling pathways under the control of SFKs were examined. SFK-targeted inhibitor-mediated inhibition was observed in the Ras/ERK, PI3K/AKT, YAP, and Cortactin pathways, with slightly varying effects based on the cell type (**Figure S8E-S8G**). Overall, we confirmed the direct effect of SFK-targeted inhibitors on cancer cells, which suggests their potential to enhance therapeutic effectiveness in combination with radiotherapy. In a mouse model bearing B16F10-luc tumors, the combination of radiotherapy and NXP900 demonstrated superior efficacy relative to each treatment alone (**Figure 7A**). Furthermore, combination therapy with two doses of NXP900 in a mouse model bearing 4T1-luc tumors exhibited improved efficacy relative to monotherapy (**Figure 7B and 7C**).

### Utilizing SFK-targeted inhibitors for concurrent radiotherapy treatment of different organs

**Figure 8:**
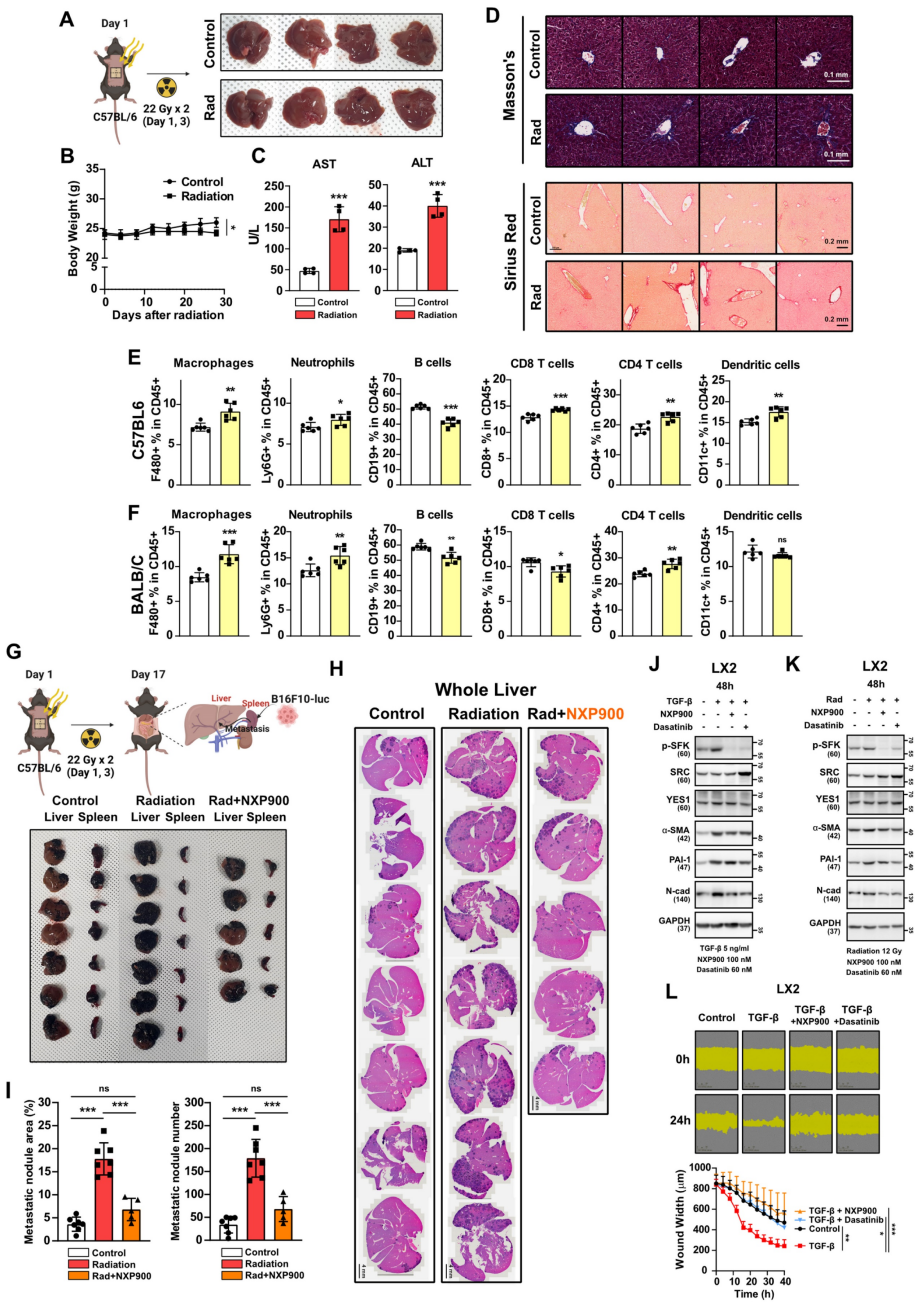
** SFK-targeted inhibitor efficacy for abdomen radiotherapy.** (**A**) Visual confirmation of liver damage after abdominal irradiation (22 Gy×2). Mice were sacrificed 4 weeks after abdominal irradiation. *n* = 4/group. (**B**) Body weight changes after abdominal irradiation. (**C**) Hematological liver damage indices, AST and ALT, after abdominal irradiation. (**D**) Detection of collagen accumulation (Masson's trichrome and Sirius Red staining) in mouse liver tissues, 4 weeks after irradiation. Representative part was selected and shown at 20× magnification. (**E, F**) Changes in immune cells infiltration in mouse liver tissue, 3 days after abdominal irradiation. *n* = 6/group. (**G**) Visual confirmation of spleen-liver metastasis caused by abdominal irradiation. B16F10 cells were injected through spleen (day 17), 2 weeks after irradiation (22 Gy×2, day 1, 3). NXP900 (60 mg/kg, daily, PO) was administered from day 1 to 16. Mice were sacrificed on day 27. *n* = 5 or 7/group. (**H**) Detection of liver metastases (H&E staining, dark purple) in mouse liver tissue. (**I**) Quantification of liver metastases. The area and number of nodules were measured. (**J**) Reversal of TGF-β-induced myofibroblast activation by SFK-inhibitors. (**K**) Reversal of direct irradiation-induced myofibroblast activation by SFK-inhibitors. (**L**) Reversal of TGF-β-induced wound healing phenotype by SFK-inhibitors. All data were presented as mean ± SD. Statistical significance of the differences was determined by two-tailed Student t-test or one-way ANOVA followed by the Tukey's test. All western blot analyses in this study were repeated three times independently.

Radiotherapy is a common treatment option not only for lung cancer but also for various solid carcinomas such as liver, breast, and prostate cancers 2. To investigate whether radiotherapy induces fibrosis and a pro-tumorigenic microenvironment in other organs, we irradiated the mouse abdomen targeting the liver, and assessed the outcomes. Four weeks after irradiation, visual examination showed no significant liver damage (**Figure 8A**). Although there was a slight weight loss attributed to radiation, no fatalities were observed (**Figure 8B**). Plasma AST or ALT levels, which are indicators of liver damage, showed mild increases (**Figure 8C**), along with an elevation in collagen accumulation, indicating early-stage liver fibrosis comparable to radiotherapy-induced early-stage lung fibrosis (**Figure 8D**). An immune cell infiltration analysis three days following abdominal radiation revealed an increase in macrophage infiltration consistent with observations in the chest irradiation model (**Figure 8E and 8F**). Next, to determine if radiation accelerates liver metastasis via formation of a pro-tumorigenic microenvironment in the liver, cancer cells were transplanted through the spleen two-weeks after abdominal irradiation. We found that spleen-to-liver metastasis had been stimulated by radiation, which was effectively reversed by NXP900 administration (**Figure 8G-8I**). Because liver fibrosis is initiated by trans-differentiation of hepatic stellate cells to myofibroblasts 40, SFK-targeted inhibitors were tested for their ability to inhibit myofibroblast activation in LX2 cells, a human hepatic stellate cell line. Both inhibitors successfully reversed myofibroblast activation as induced by the TGF-β or direct irradiation (**Figure 8J and 8K**), and they also mitigated wound healing caused by TGF-β treatment in LX2 cells (**Figure 8L**). These data suggest that SFK-targeted inhibitors show promise in counteracting the formation of a pro-tumorigenic microenvironment in the liver as well as the lungs.

### Effectiveness of SFK-targeted inhibitors in late-stage lung fibrosis

**Figure 9:**
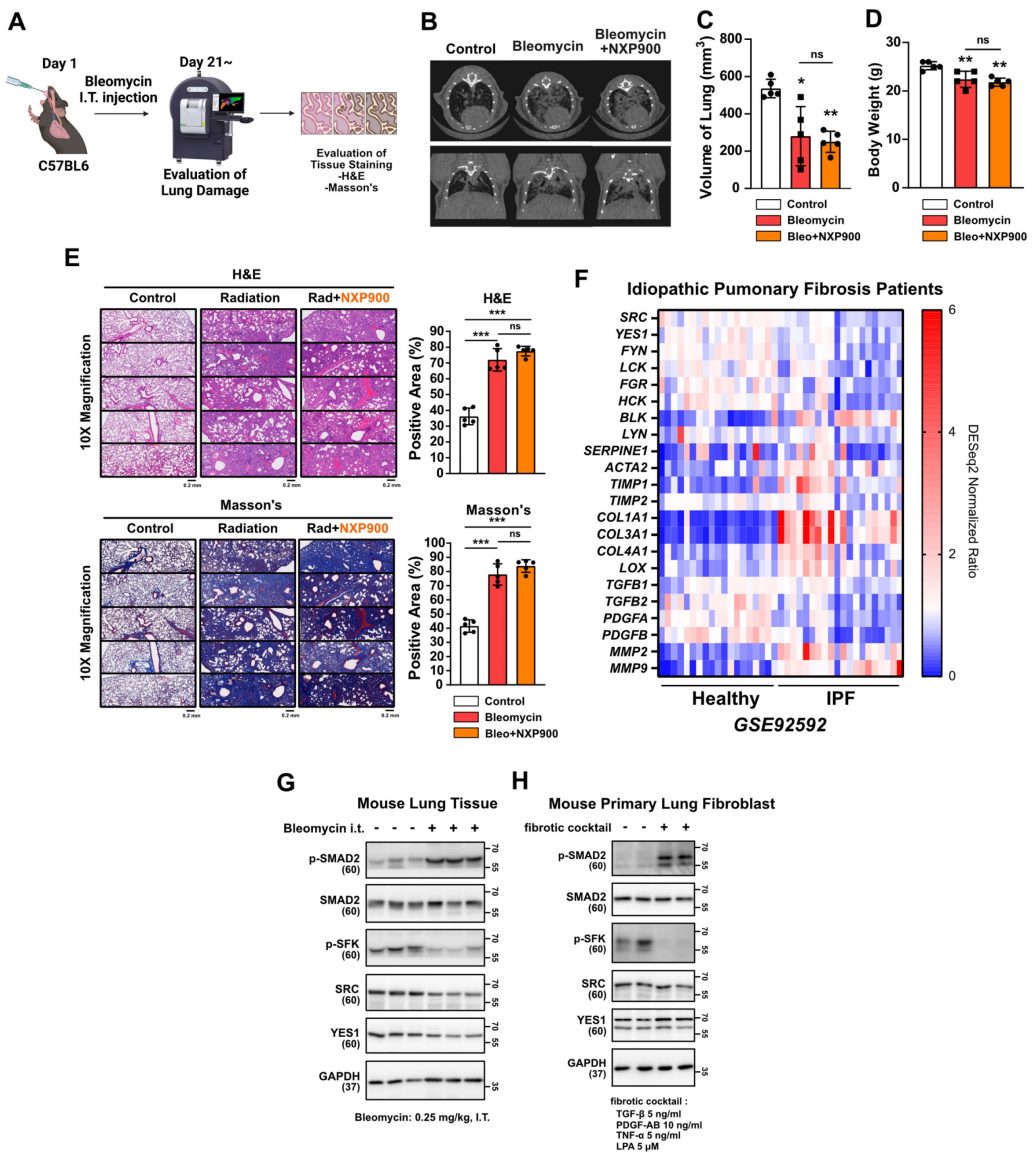
** Effects of SFK-inhibitor in late-stage lung fibrosis.** (**A**) Efficacy of NXP900 in bleomycin-induced lung fibrosis model. Bleomycin was administered intratracheally at 0.25 mg/kg, 40 μL (day 1). NXP900 was administered orally (60 mg/kg, daily, day 2-21).* n* = 5/group. (**B-D**) CT scans to detect lung injury, 3 weeks after bleomycin treatment. Functional lung volume was calculated using micro-CT software. (**E**) Detection of alveolar collapse (H&E) and collagen accumulation (Masson's trichrome) in mouse lung tissues, 3 weeks after bleomycin treatment. Whole lung cross-section data are shown in Figure S10. (**F**) Comparison gene expression of fibrosis markers in lung tissues between IPF patients with healthy individuals. The NCBI public dataset, GSE92592, was used. (**G**) SFKs activation in bleomycin-induced mouse lung tissue. (**H**) SFKs activation by fibrotic cocktail treatment (5 ng/ml TGF-β, 10 ng/ml PDGF-AB, 5 ng/ml TNF-α and 5 μM LPA). All data were presented as mean ± SD. Statistical significance of the differences was determined by two-tailed Student t-test or one-way ANOVA followed by the Tukey's test. All western blot analyses in this study were repeated three times independently.

Idiopathic pulmonary fibrosis (IPF) is a challenging disease characterized by progressive lung fibrosis with an uncertain etiology, as is often associated with poor prognosis 41. The FDA-approved drugs pirfenidone and nintedanib, currently used for treatment of IPF patients, lack sufficient efficacy 42. In this study then, we explored the potential therapeutic benefits of SFK-targeted inhibitors in a mouse model mimicking late-stage lung fibrosis (**Figure 9A**). It is known that intratracheal administration of bleomycin, widely employed, induces robust lung fibrosis resembling IPF 43. Three weeks after bleomycin insult in the present study, CT scans revealed that NXP900 administration had not reversed lung fibrosis (**Figure 9B-9D**). Additionally, tissue staining showed no reversal of the bleomycin-induced alveolar collapse or collagen accumulation (**Figure 9E**). In an analysis of gene expression in lung tissues from 20 IPF-diagnosed patients using public data, SFKs gene expression was found to be decreased, inconsistent with radiotherapy-induced lung fibrosis (**Figure 9F**). Furthermore, it was confirmed that SFKs activity was diminished in IPF-mimic animal and cell models (**Figure 9G and 9H**). In summary, SFK-targeted inhibitors are not effective in treating advanced stages of lung fibrosis such as IPF. This suggests that effective application of SFK-targeted inhibitors may be confined to an early-stage of lung fibrosis such as radiotherapy-induced lung fibrosis.

## Discussion

RILI is a prevalent side effect affecting approximately 50% of patients undergoing thoracic radiotherapy 3, 4. It has the potential to progress into lung fibrosis, thereby promoting a pro-tumorigenic lung microenvironment 2, 13, 27. Some cohort studies have indicated that thoracic radiotherapy may increase local recurrence rates in patients with breast and lung cancer 11, 12. Thus, while radiotherapy is widely recognized as an effective for treating primary tumors, awareness of its potential risks for secondary recurrence and metastasis is crucial. While recent studies have aimed to elucidate the mechanisms driving the formation of a radiotherapy-induced pro-tumorigenic microenvironment 7, 9, the precise mechanisms remain elusive, and currently, no drugs are available for preventing or treating this condition. Hence, it is imperative to clarify the mechanisms underlying the development of a pro-tumorigenic microenvironment induced by radiation and to propose potential therapeutic strategies.

A recent study demonstrated that chest irradiation in both BALB/c and NSG mice resulted in neutrophil infiltration and granulation, activating Notch signaling in lung epithelial cells 7. Our study confirmed neutrophil infiltration in BALB/c mice, as was consistent with that previous report (**Figure 2B**). However, C57BL/6 mice did not exhibit radiation-induced neutrophil infiltration (**Figure 2A**), implying potential strain-dependent variations in the immune cells involved in RILI. Considering that cytotoxic T cells are key players influencing cancer cell death 44, it is plausible that cytotoxic T cells have a role in the formation of a radiotherapy-induced pro-tumorigenic microenvironment. However, irradiation still induces a pro-tumorigenic microenvironment in T cell-deficient BALB/c-nude mice (**Figure 6C**). Additionally, PD-1 antibody administration failed to reverse the radiotherapy-induced pro-tumorigenic microenvironment (**Figure 3B**), indicating that cytotoxic T cells may not play a pivotal role in this process, at least not in mouse radiation models. Rather, we identified that macrophages, implicated in both strains and organs (lung and liver), emerged as significant contributors to the radiation-induced reshaping of the tumor microenvironment (**Figure 2A, 2B, 8E, and 8F**).

This study emphasizes SFKs, specifically SRC and YES1, as a main regulator in the development of a radiotherapy-induced pro-tumorigenic microenvironment. SFKs, a family of non-receptor protein tyrosine kinases, is recognized for its involvement in various cellular processes, including proliferation, differentiation, and apoptosis 16, 17. Although the functional roles of SFKs in tumorigenesis have been extensively studied 18, 19, 39, few recent studies have focused on their roles in tissue fibrosis 20, 21. It has been reported that SFKs members Fyn and Fgr are involved in liver and lung fibrosis, respectively 22, 23. Nonetheless, the functions of SFKs, especially SRC and YES1, in altering the tumor microenvironment influenced by fibrosis remain largely unclear. In the present study, SRC and YES1 exhibited elevated expression and activation in lung tissues from both lung cancer patients and mice exposed to radiation (**Figure 4A, 4D, and 4E**). Moreover, TGF-β treatment or direct irradiation induced SFKs activation in lung epithelial cells (**Figure 4G and 5C**). Specifically, by analyzing the cell type-specific expression of SFKs and co-expression with fibrosis-related gene sets, we identified SRC and YES1 as crucial factors in radiotherapy-induced fibrosis (**Figure 4B and 4C**).

Differentiation of macrophages into M2 and subsequent release of TGF-β, acting via the canonical pathway, SMAD2, are recognized as pivotal elements in tissue fibrosis 45, 46. Our study revealed that TGF-β treatment induced activation of SFKs and SMAD2 in lung epithelial cells and fibroblasts (**Figure 4G and 4H**). SFKs inhibition did not affect SMAD2, AKT or RAS activation but did significantly reduce p70S6K activation (**Figure 4J and 4K**), suggesting that SFKs influence the non-canonical myofibroblast activation pathways through p70S6K (**Figure 4P**). Notably, direct irradiation to lung epithelial cells exclusively activated SFKs without impacting the canonical pathway, and induced only an increase in PAI-1 expression, unlike TGF-β-mediated canonical signaling (**Figure 5D and 5E**). Unfortunately, this study could not unveil this mechanism, which fact highlights the need for additional mechanistic research.

We conducted comprehensive investigations into the effects of an SFK-targeted inhibitor on macrophages, given their elevated expression of SFKs. Our findings indicate that while SFK-targeted inhibitors may induce a slight increase in M2 activity in differentiating macrophages, their overall impact on fully differentiated macrophages was limited (**Figure S6**). This was further supported by results from animal experiments (**Figure 7H**). Notably, NXP900 demonstrated beneficial effects on fibrosis and the pro-tumorigenic microenvironment in animal models, suggesting that the application of SFK-targeted inhibitors may still offer advantages in the treatment of radiotherapy-induced fibrosis.

IPF being a late-stage pulmonary fibrosis of unknown cause that typically is diagnosed late and in an advanced state, the prognosis for patients with this affliction is generally poor 41. While two drugs have been FDA approved for IPF, they have not yet demonstrated sufficiently therapeutic effects 42. We investigated whether an SFK-targeted inhibitor could exhibit therapeutic efficacy for IPF. Interestingly, NXP900 showed no efficacy in the bleomycin-induced fibrosis mouse model (**Figure 9A-9E**). Considering the decreased SFKs expression in the lung tissues of IPF patients and the diminished SFKs activity in both mouse lung tissues and primary lung fibroblasts, as suggestive of IPF pathology (**Figure 9F-9H**), SFKs seem not to be a significant target in late-stage lung fibrosis.

In this study, we applied two SFK-targeted inhibitors: dasatinib, approved by the FDA for chronic myeloid leukemia 36, and NXP900, a novel SRC/YES1-inhibitor currently undergoing clinical trial (NCT05873686). Whereas both inhibitors demonstrated efficacy in preventing a radiotherapy-induced pro-tumorigenic microenvironment, we propose that NXP900 is a more selective and potent inhibitor to prevent radiotherapy-induced lung fibrosis and reshaping of pro-tumorigenic microenvironment (**Figure 6-8**). Unlike dasatinib, NXP900 makes structural changes in SFKs proteins, leading to closed conformation with significantly reduced activity 37. Moreover, NXP900 does not inhibit ABL, another major target of dasatinib, indicating its better selectivity profiles in comparison with dasatinib 37, 38. In conclusion, this study identified SFKs as a crucial factor in the development of a radiotherapy-induced pro-tumorigenic lung microenvironment and proposes NXP900 as a novel SFK-targeted inhibitor. NXP900 is anticipated to positively impact cancer patient survival, particularly when applied in the early-stages of lung fibrosis, including radiotherapy-induced lung injury. The discovery that a pro-tumorigenic microenvironment is triggered even in cases of clinically imperceptible damage emphasizes the importance of proactively incorporating combination therapy that integrates SFK-targeted inhibitors with radiotherapy.

## Materials and Methods

### Cell lines and mice

The used cell lines and their respective culture methods are detailed in **Table S1**. B16F10, MRC5, THP1 cell lines were sourced from Korean Cell Line Bank (Seoul, Korea). The B16F10 cells were transfected with lentiviral particles containing CMV-luciferase (firefly) gene (#LVP009, AMSBIO, Cambridge, MA, USA) to establish B16F10-luc cell line. The 4T1 and 4T1-luc cell lines were obtained from Dr. Byun Y (Seoul National University). The BEAS-2B cell line was obtained from Dr. Chung KH (Sungkyunkwan University), and the LX2 cell line from Dr. Friedman SL (Icahn School of Medicine at Mount Sinai). All cell lines were maintained in a humidified incubator at 37℃ with 5% CO_2_.

In all animal experiments, 7-9 weeks old male C57BL/6 and BALB/c mice (Raonbio, Seoul, Korea) were maintained in the specific-pathogen-free facility of Seoul National University Institute of Laboratory Animal Resources (Seoul, South Korea).

### Irradiation

For *in vivo* radiation model, mice received two fractions of 22 Gy (22 Gy×2) on chest or abdomen, with a targeting area of 1.5 cm × 1 cm, utilizing a beam-guided collimator (**Figure S1**). For the left lung-targeted radiation model, a single dose of 44 Gy was delivered to the middle part of the left lung, focusing on an area of 0.2 cm × 0.2 cm. The irradiation procedures were conducted using the X-RAD 320 (Precision, Madison, CT, USA) equipped with a Dynamic Collimator (Precision) and an F2 filter (1.5 mm Al + 0.25 mm Cu + 0.75 mm Sn) at 320 KV, 12.5 mA, and a source-to-shelf distance (SSD) of 33 cm. Cell irradiation was conducted using the X-RAD 160 (Precision). The procedure involved the use of an F1 filter (2 mm Al) at 160 KV, 19 mA, and a SSD of 50 cm.

### Evaluation of lung metastasis after irradiation

14 days after chest irradiation, either 6×10^5^ 4T1-luc cells (BALB/c) or 4×10^5^ B16F10-luc cells (C57BL/6) were intravenously injected via the tail vein. BALB/c-nude mice were injected with 1.5×10^5^ 4T1-luc cells. Bioluminescence intensity in the lung was detected using IVIS Spectrum In Vivo Imaging System (PerkinElmer, Waltham, MA, USA). For luminescence, 150 mg/kg D-Luciferin Potassium Salt (#122799, PerkinElmer) was injected intraperitoneally into the mice 10 min before detection. The survival rate was determined by documenting the date of death of the mice. Ethical approval for all procedures was obtained from the Institutional Animal Care and Use Committee of Seoul National University (#SNU-230228-3, #SNU-230624-3, #SNU-230916-3, #SNU-231111-2).

### Reverse transcriptase-quantitative polymerase chain reaction (RT-qPCR) and small interfering RNA transfection

Total RNA extraction was carried out using Trizol reagent (#15596018, ThermoFisher, Waltham, MA, USA), chloroform, and absolute ethanol following the manufacturer's protocol. cDNA synthesis was accomplished by using 1 μg of total RNA with the Maxime RT PreMix kit (#25081, IntronBio, Seongnam, South Korea), followed by qPCR using SYBR green supermix (#1708884, Bio-Rad laboratories, Hercules, CA, USA) on the CFX Connect Real-Time PCR System (Bio-Rad laboratories). The specific primer sequences employed are detailed in **Table S2**.

The knockdown of *YES1 and SRC* was accomplished using Lipofectamine 2000 transfection reagents (#11668027, ThermoFisher) according to the manufacturer's protocol. Briefly, to start the transfection process, Lipofectamine 2000 and TOM media (transfection optimized medium, #TR004-01, WELGENE, Gyeongsan, South Korea) were mixed and incubated for 5 min. Subsequently, 100 *p*mol siRNA was added to the mixture and incubated for an additional 20 min, and cells were incubated with the preincubated mixture for 24 h. Predesigned *siYES1* pools (No. 7525-1,2,3), *siSRC* pools (No. 6714-1,2,3), and negative control siRNA (#SN-1001) were purchased from Bioneer (Daejeon, South Korea).

### *In vivo* fibrosis evaluation: micro-CT and immunohistochemistry (IHC)

To assess lung damage in mice in a clinically relevant manner, chest CT images were obtained one month after lung irradiation using a Quantum GX2 Micro-CT Imaging System (PerkinElmer). The Micro-CT utilized an X-ray filter (0.06 mm Cu + 0.5 mm Al) at 90 KV, 0.088 mA. Subsequent analysis of mouse lung volume was carried out using Caliper Micro-CT analysis software (Caliper LifeSciences, Hopkinton, MA, USA).

For IHC, the lung and liver tissues were dissected from sacrificed mice. Paraffin blocks were prepared and subsequent staining was conducted using Hematoxylin and Eosin (H&E), Masson's trichrome, or Sirius Red. Stained slides were photographed using a Vectra instrument (PerkinElmer). 10× magnification images were analyzed using ImageJ software (Bethesda, MD, USA). All 1x magnification images are presented in **Figure S10**. Ethical approval for all procedures was obtained from the Institutional Animal Care and Use Committee of Seoul National University (#SNU-230325-1, #SNU-231029-1). The used IHC antibodies are listed in **Table S3**.

### Immune cell analysis

The isolated tissues were processed using a gentleMACS dissociator (Miltenyi Biotec) with dissociation kit (#130-096-730, Miltenyi Biotec, Bergisch Gladbach, Germany). Immune cells were selectively isolated from the dissociated tissues using percoll gradient and then stained with flow cytometry antibodies. The analysis of immune cells was performed using Novocyte flow cytometer (Agilent, Santa Clara, CA, USA). The used flow cytometry antibodies are listed in **Table S3** and the gating strategies are illustrated in **Figure S2 and S3**. Ethical approval for all procedures was obtained from the Institutional Animal Care and Use Committee of Seoul National University (#SNU-230513-2, #SNU-230520-1, #SNU-231031-1).

### Bone marrow derived monocyte isolation and macrophage differentiation

After trimming the femur and tibia with scissors, the bone marrow was extruded using a syringe. The extruded bone marrow was pipetted thoroughly in RPMI-1640 medium containing 10% FBS and 1% P/S, followed by centrifugation at 500×g for 5 min to collect cells. Red blood cells from the obtained cell pellets were removed by ACK lysis buffer (Gibco, Gaithersburg, MD, USA). The bone marrow cells were seeded into a petri dish, and treated with 30 ng/ml macrophage-colony stimulating factor (M-CSF, Peprotech, Rocky Hill, NJ, USA) for 6 days to induce macrophage differentiation. Subsequently, the differentiated macrophages were detached from the petri dish and utilized for further experiments. As a human macrophage surrogate cells, THP1 cells were treated with 25 ng/ml phorbol myristate acetate (PMA) for 3 days.

### Transfusion of Vivotrack680-labeled monocytes and monocyte-derived macrophages

Mouse bone marrow cells were exposed to M-CSF for 3 days, and subsequently labeled with Vivotrack680 (#NEV12000, PerkinElmer). A total of 3×10^6^ stained cells were transfused through the mouse tail vein. Three days after injection, *in vivo* detection of Vivotrack680 fluorescence was conducted using an IVIS Spectrum (PerkinElmer). After sacrifice, the lungs were dissected to analyze the infiltration of Vivotrack680-labeled cells. Ethical approval for all procedures was obtained from the Institutional Animal Care and Use Committee of Seoul National University (#SNU-230513-2).

### Enzyme-linked immunosorbent assay (ELISA)

To determine the amount of TGF-β1 secretion in mouse lung tissue, whole mouse lungs were lysed in PBS using a gentleMACS dissociator (Miltenyi Biotec). The lungs of one mouse was dissolved in 45 ml of PBS and used for ELISA. To measure the amount of TGF-β1 secreted from mouse BMDM, cells were seeded at 5 ×10^5^ cells/2 ml in a 6-well plate. Supernatants obtained from each experiment were measured using the DuoSet mouse TGF-beta 1 ELISA kit (#DY1679, R&D Systems, Minneapolis, MN, USA).

### Western blot analysis

Cell lysis was initiated using a lysis buffer composed of 10 mM Tris-Cl, 1% Triton X-100, 100 mM sodium chloride, 10% glycerol, 1 mM EDTA, 30 mM sodium pyrophosphate, 5 mM glycerol-2-phosphate, 1 mM sodium fluoride, 1 mM sodium orthovanadate, 1% phosphatase inhibitor cocktail 2 (#P5726, Sigma-Aldrich, St. Louis, MO, USA), 1% phosphatase inhibitor cocktail 3 (#P0044, Sigma-Aldrich), and 0.02 tablet/ml protease inhibitor cocktail (#11697498001, Roche, Basel, Switzerland). Bradford assay was employed for quantifying the protein concentrations in the samples. The protein samples were then separated on SDS-polyacrylamide gels and transferred to 0.45 μm nitrocellulose membranes (#10600002, Cytiva, Marlborough, MA, USA). Subsequently, membranes were blocked with 5% skim milk in phosphate-buffered saline with 0.1% Tween 20 (PBST), and incubated with primary antibodies overnight at 4℃. Secondary antibodies were applied for 1 h at room temperature. Membranes were visualized using a chemiluminescent HRP substrate (#WBKLS0500, Merck Millipore, Burlington, MA, USA) and detected by LAS-3000 mini (Fujifilm, Tokyo, Japan). The used antibodies are listed in **Table S3**. All western blot analyses in this study were repeated three times at least independently. All immunoblot full images are presented in Supplementary Materials.

### Wound healing assay

BEAS-2B, MRC5, and LX2 cells were seeded at 90-95% confluence in the IncuCyte Imagelock 96-well plate (#BA-04856, Sartorius, Gottingen, Germany). The next day, the medium was replaced with serum free medium, and a wound scratch was made using an IncuCyte 96-well wound maker (EssenBioscience, Ann Arbor, MI, USA). The extent to which cells migrate and recover from wounds was scanned and analyzed using an IncuCyte Zoom device (EssenBioscience).

### Lung orthotopic radiotherapy model

6×10^5^ 4T1-luc cells or 4×10^5^ B16F10-luc cells were intravenously injected via the tail vein. 6 days after cancer cell injection, mice were randomly separated, and NXP900 treatment or radiotherapy was initiated. Bioluminescence intensity in the lung was detected using the IVIS Spectrum In Vivo Imaging System (PerkinElmer). The survival rate was determined by documenting the date of death of the mice. Ethical approval for all procedures was obtained from the Institutional Animal Care and Use Committee of Seoul National University (#SNU-230823-4).

### Spleen-liver metastasis evaluation after irradiation

14 days after abdomen irradiation, 1×10^5^ B16F10 cells were injected into the spleen. 10 days after the spleen injection of cancer cells, the mice were sacrificed, and the liver and spleen were dissected to assess the extent of metastasis through tissue staining. Metastatic nodule areas and numbers were analyzed using ImageJ software. Ethical approval for all procedures was obtained from the Institutional Animal Care and Use Committee of Seoul National University (#SNU-231212-1).

### *In vivo* treatment of drugs

To evaluate the efficacy of SFK-targeted drugs in animals, mice were treated with NXP900 (60 mg/kg, dissolved in 3 mM sodium citrate buffer) or dasatinib (30 mg/kg, dissolved in 3 mM sodium citrate buffer) once daily by oral gavage for two weeks. For non-specific macrophage suppression, zoledronate (0.2 mg/kg, dissolved in PBS) was administered once a week through intraperitoneal injection. The administration of zoledronate was initiated one week prior to irradiation. Anti-mouse F4/80 (10 mg/kg) or anti-mouse PD-1 (10 mg/kg), were administered twice a week through intraperitoneal injection. The administration of neutralizing antibodies initiated three days before the irradiation. The used neutralizing antibodies are listed in **Table S3**.

### Isolation of mouse lung fibroblasts

Mouse lung tissues were dissociated using gentleMACS dissociator (Miltenyi Biotec) in DMEM/F12 medium with 0.14 Wunsch units/ml Liberase Blendzyme 3 and 1× antibiotic/antimycotic. Following dissociation, lung tissues were pipetted thoroughly in DMEM/F12 medium containing 10% FBS and 1% P/S, and then centrifuged at 500*g* for 5 min to collect cells. The obtained cell pellets were resuspended and seeded into a culture dish. After one week of seeding, cells were detached from the culture dish and used for required experiments.

### Late-stage lung fibrosis mouse model: bleomycin-induced lung fibrosis

C57BL/6 mice were intratracheally administered with 0.25 mg/kg bleomycin sulfate. The day following bleomycin administration, the groups were randomly divided, and NXP900 was orally administered daily. Three weeks later, chest imaging was performed using the Quantum GX2 micro-CT Imaging System (PerkinElmer). After imaging, mouse lungs were collected, paraffin blocks were prepared, and H&E and Masson's trichrome staining were conducted. Stained slides were captured using a Vectra instrument (PerkinElmer) and analyzed using ImageJ software (Bethesda, MD, USA). Ethical approval for all procedures was obtained from the Institutional Animal Care and Use Committee of Seoul National University (#SNU-231111-1).

### Public data

Survival rate analysis based on gene expression in cancer patients was conducted utilizing the Gene Expression Profiling Interactive Analysis (GEPIA) web server 47. Additionally, correlation analysis of multiple genes was carried out using the GEPIA web server. Examination of the expression of various SFKs genes and the results of single-cell sequencing of lung tissues were obtained from the Human Protein Atlas database 48. For the analyses of various patient samples, the NCBI GEO database were used: GSE162945 49, GSE179810, and GSE92592 50. For heat map analysis using the NCBI GEO dataset, DESeq2 normalization was implemented.

### Statistical analysis

Statistical significance was determined using GraphPad Prism 7.0. The significance between experimental groups were analyzed using either one-way ANOVA followed by Tukey's test or an unpaired two-tailed Student t-test. *P* values less than 0.05 were considered as significant differences. *P* value presented in this study follows the NEJM style; *p < 0.05, **p < 0.01, ***p < 0.001.

## Supplementary Material

Supplementary methods, figures and tables.

Supplementary data.
