# Supplementary material for: Prevention of radiotherapy-induced pro-tumorigenic microenvironment by SFK inhibitors
Source: Theranostics. 2025 Jan 1;15(3):875–93. doi: 10.7150/thno.100970 (PMC11700852; doi:10.7150/thno.100970)
Supplement: Supplementary file 1 — Supplementary methods, figures and tables. [file thnov15p0875s1.pdf]

**Supplementary Information: Methods/Tables/Figures**

# Prevention of radiotherapy-induced pro-tumorigenic microenvironment by SFK inhibitors

Yong June Choi[1], Myung Jun Kim[1], Young Joo Lee[1], Munkyung Choi[1], Wan Seob Shim[1], Miso Park[2], Yong-Chul Kim[3] and Keon Wook Kang[1,*]

[1]College of Pharmacy, Research Institute of Pharmaceutical Sciences and Natural Products Research Institute, Seoul National University, Seoul 08826, Republic of Korea. [2]Department of Pharmacy, Kangwon National University, Chuncheon 24341, Republic of Korea. [3]School of Life Sciences, Gwangju Institute of Science and Technology, Gwangju 61005, Republic of Korea.

**Corresponding Author:** Keon Wook Kang, Ph.D. E-mail: kwkang@snu.ac.kr

# Supplementary Materials and Methods

## Immunohistochemistry (IHC)

For IHC, the lung tissues were dissected from sacrificed mice. Paraffin blocks were prepared and subsequent staining was conducted using specific F4/80 antibody or TGF-beta 1 antibody. Stained slides were photographed using a Vectra instrument (PerkinElmer). The used IHC antibodies are listed in **Table S3**.

## Cytokine array

The mouse chest underwent a single irradiation with 22 Gy, and the mouse lungs were dissected three days later. Isolated lung tissues were processed according to the cytokine array manufacturer's manual (#AAM-BLM-1-2, Raybiotech, Norcross, GA, USA). Briefly, the isolated lungs were lysed using the provided lysis buffer, and supernatant was quantified and biotinylated. The biotinylated sample underwent a reaction with the membrane, and following washing, HRP-conjugated streptavidin was applied. Membranes were visualized and detected by LAS-3000 mini (Fujifilm).

## Macrophage cell viability assessment

Macrophages were seeded in 96-well plates ($2 \times 10^4$ cells/well) and were treated with NXP900. After 24 h incubation, 10 μL WST-8 solution (#CM-VA1000, Precaregene, Anyang, South Korea) were added and incubated for additional 4 h in the dark. The absorbance at 450 nm was measured using a multi-mode microplate reader (SpectraMax iD3, Molecular Devices, San Jose, CA, USA) to calculate the number of viable cells.

## M2 differentiation of macrophages

The mouse bone marrow cells were seeded into a petri dish, and treated with 30 ng/ml

macrophage-colony stimulating factor (M-CSF) for 6 days to induce macrophage differentiation. Differentiated macrophages were treated with TGF-β 5 ng/ml or IL-4 20 ng/ml for 24 h for further M2 differentiation.

## *In vitro* proliferation and migration assays

For the proliferation assay, cancer cells were seeded in 96-well plate (2 x $10^3$ cells/well), and after the drug treatment, cell confluence was scanned and analyzed by the IncuCyte Zoom device (EssenBioscience). For the transwell migration assay, cancer cells (3,000 cells/upper well) were seeded in the IncuCyte Clearview 96-well plate (#4582, Sartorius). To create a gradient of chemoattractant, 1% FBS was added to the upper well, while 15% FBS was added to the lower well. Drugs were added to both the upper and lower wells at the indicated concentrations. The number of cells above and below was scanned and analyzed by the IncuCyte Zoom device (EssenBioscience).

## Statistical analysis

Statistical significance was determined using GraphPad Prism 7.0. The significance between experimental groups were analyzed using either one-way ANOVA followed by Tukey's test or an unpaired two-tailed Student t-test. *P* values less than 0.05 were considered as significant differences. *P* value presented in this study follows the NEJM style; $*p < 0.05$, $**p < 0.01$, $***p < 0.001$.

# Supplementary Tables

**Table S1.** The used cell lines and their respective culture methods.

| Cell line | Culture Medium |
|---|---|
| **4T1** | RPMI-1640 medium + 10% FBS + 1% P/S |
| **4T1-luc** | RPMI-1640 medium + 10% FBS + 1% P/S |
| **B16F10** | High glucose DMEM medium + 10% FBS + 1% P/S |
| **B16F10-luc** | High glucose DMEM medium + 10% FBS + 1% P/S |
| **THP1** | RPMI-1640 medium + 10% FBS + 1% P/S |
| **MRC5** | RPMI-1640 medium + 10% FBS + 1% P/S |
| **BEAS2B** | High glucose DMEM medium + 10% FBS + 1% P/S |
| **LX2** | High glucose DMEM medium + 10% FBS + 1% P/S |

DMEM: Dulbecco's modified Eagle's medium

FBS: Fetal bovine serum, Thermo Fisher Scientific, Waltham, MA, USA

P/S: penicillin/streptomycin, Hyclone, Logan, UT, USA

**Table S2.** The specific primer sequences employed in study.

| Name | Forward (5'-3') | Reverse (5'-3') |
|---|---|---|
| ***Timp1*** | CTTGGTTCCCTGGCGTACTC | ACCTGATCCGTCCACAAACAG |
| ***Acta2*** | GGCTCTGGGCTCTGTAAGG | CTCTTGCTCTGGGCTTCATC |
| ***Col1a1*** | GCCCGAACCCCAAGGAAAAGAAGC | CTGGGAGGCCTCGGTGGACATTAG |
| ***Tgf-beta1*** | TCGACATGGAGCTGGTGAAA | GGGACTGGCGAGCCTTAGTT |
| ***Src*** | GTTGCTTCGGAGAGGTGTGGAT | CACCAGTTTCTCGTGCCTCAGT |
| ***Yes1*** | GGTCTGGCAAAAGATGCTTGGG | GGCATCATTGTACCTGGCTTTAG |
| ***18s rRNA*** | AGGATCCATTGGAGGGCAAGT | TCCAACTACGAGCTTTTTAACTGCA |

**Table S3.** The used antibodies.

## Antibodies for western blotting

| Name | Company | Cat No. |
|---|---|---|
| **COX-2** | Abcam (Cambridge, UK) | Ab15191 |
| **Arginase-1** | Santa Cruz Biotechnology (Dallas, TX, USA) | sc-271430 |
| **YAP** | | sc-376830 |
| **GAPDH** | Merck Millipore (Burlington, MA, USA) | CB1001 |
| **Mouse YES1** | Proteintech (Rosemont, IL, USA) | 20243-1-AP |
| **Phospho-SFK** | Cell Signaling Technology (Danvers, MA, USA) | 2101s |
| **SRC** | | 2108s |
| **YES1** | | 3201s |
| **Phospho-Smad2** | | 3101s |
| **Phospho-p70S6K** | | 9234s |
| **Phospho-AKT** | | 9271s |
| **Phospho-MEK** | | 9154s |
| **Phospho-ERK** | | 9101s |
| **Phospho-YAP** | | 4911s |
| **Phospho-Cortactin** | | 4569s |
| **SMAD2** | | 5339s |
| **p70S6K** | | 9202s |
| **AKT** | | 9272s |
| **MEK** | | 8727s |
| **ERK** | | 9102s |
| **Cortactin** | | 3502s |
| **HRP-linked anti-rabbit IgG** | | 7074s |

| | | |
|---|---|---|
| **HRP-linked anti-mouse IgG** | | 7076s |
| **α-SMA** | Sigma-Aldrich (St. Louis, MO, USA) | A5228 |
| **PAI-1** | BD Biosciences (Franklin Lakes, NJ, USA) | 612024 |
| **E-cadherin** | | 610181 |
| **N-cadherin** | | 610920 |

## Antibodies for flow cytometry

| Name | Company | Cat No. |
|---|---|---|
| **PE anti-mouse CD45** | Biolegend (San Diego, CA, USA) | 103105 |
| **APC anti-mouse F4/80** | | 123115 |
| **FITC anti-mouse Ly6G** | | 127605 |
| **APC/Cy7 anti-mouse CD19** | | 115530 |
| **APC anti-mouse CD8** | | 100711 |
| **FITC anti-mouse CD4** | | 100509 |
| **APC/Cy7 anti-mouse CD11c** | | 117324 |
| **FITC anti-mouse CD206** | | 141703 |
| **APC/Cy7 anti-mouse CD86** | | 105029 |
| **PE/Cy7 anti-mouse CD64** | | 139313 |
| **PE/Cy5 anti-mouse CD11b** | | 101209 |
| **APC anti-mouse SiglecF** | | 155507 |

## Antibodies for in vivo neutralization

| Name | Company | Cat No. |
|---|---|---|
| **InVivoMAb anti-mouse F4/80** | Bio X Cell (Lebanon, NH, USA) | BE0206 |
| **InVivoMAb anti-mouse PD-1** | | BE0146 |

## Antibodies for IHC

| Name | Company | Cat No. |
|---|---|---|
| **F4/80** | Cell Signaling Technology<br>(Danvers, MA, USA) | 70076s |
| **TGF-beta 1** | Sigma-Aldrich<br>(St. Louis, MO, USA) | SAB4502954 |

# Supplementary Figures

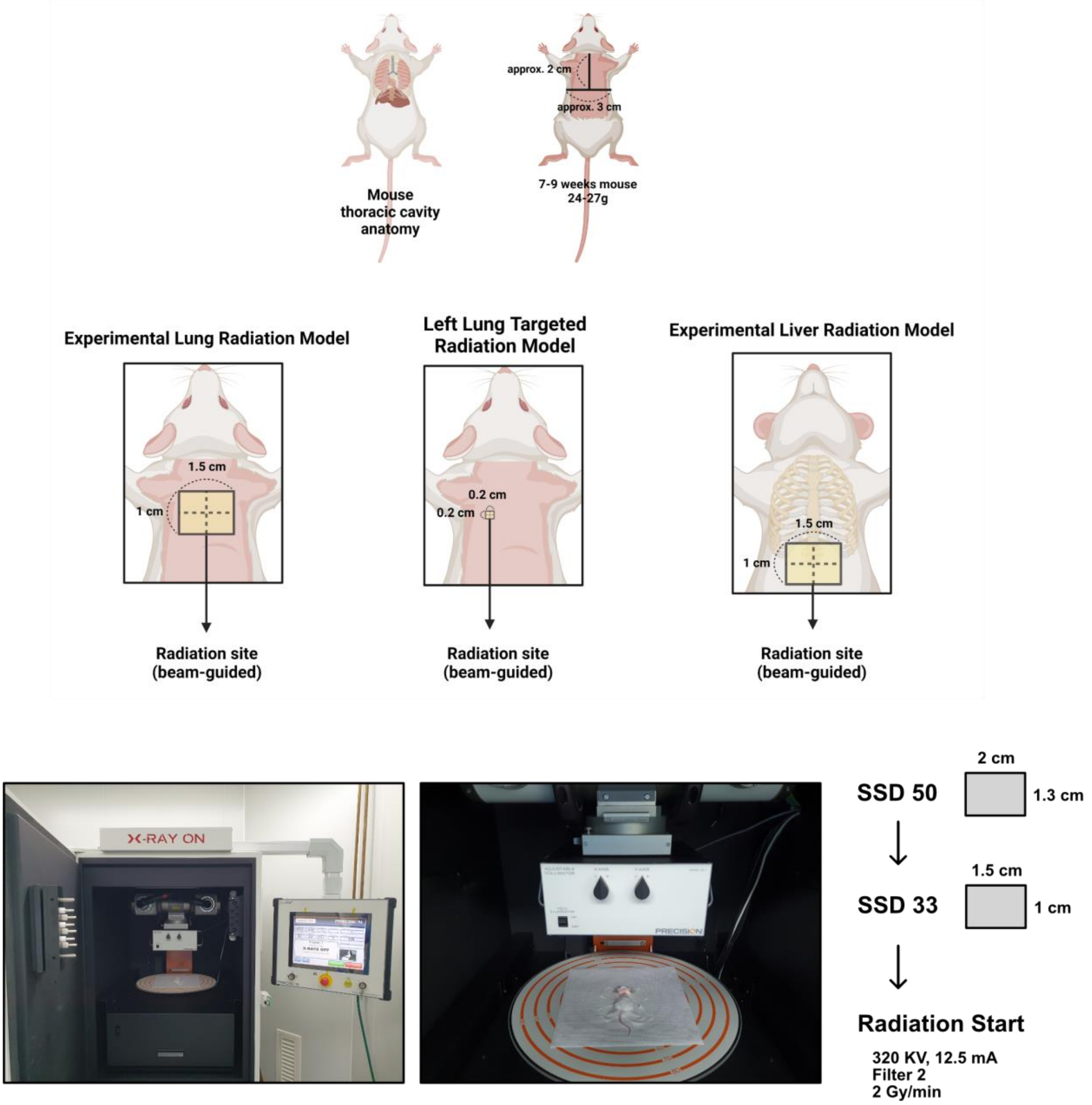


**Figure S1. Experimental mouse radiotherapy model**

Schematic illustration of experimental mouse radiotherapy model. For the experimental implementation of lung radiotherapy, radiation was delivered to a 1.5 cm×1 cm area on the dorsal of the thoracic cavity (left). To mimic stereotactic body radiotherapy, radiation was delivered to a 0.2 cm×0.2 cm area on the dorsal of the left thoracic cavity (middle). For the experimental implementation of liver radiotherapy, radiation was delivered to a 1.5 cm×1 cm

area on the abdomen (right). The irradiation procedures were conducted using the X-RAD 320 (Precision) equipped with a Dynamic Collimator (Precision) and an F2 filter.

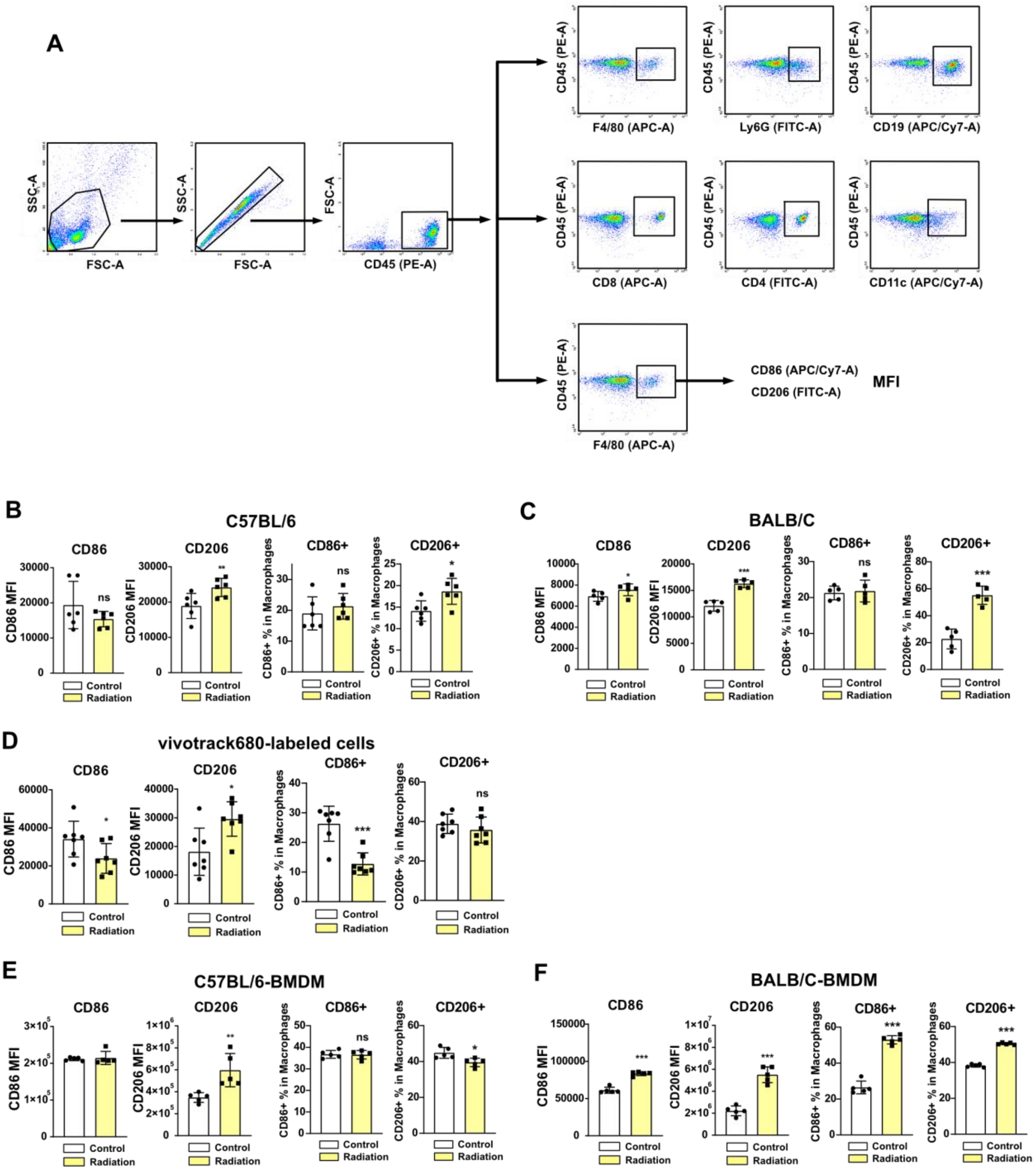


**Figure S2. Gating strategies and individual values of M1, M2 markers**

(**A**) Gating strategy for Figure 2 and 8. The used flow cytometry antibodies are listed in Table S3. (**B-F**) Percentage of M1 and M2 macrophages and individual values of CD86 and CD206 MFI about Figure 2C, D, F, and I, respectively. All data were presented as mean ± SD. Statistical significance of the differences was determined by two-tailed Student t-test.

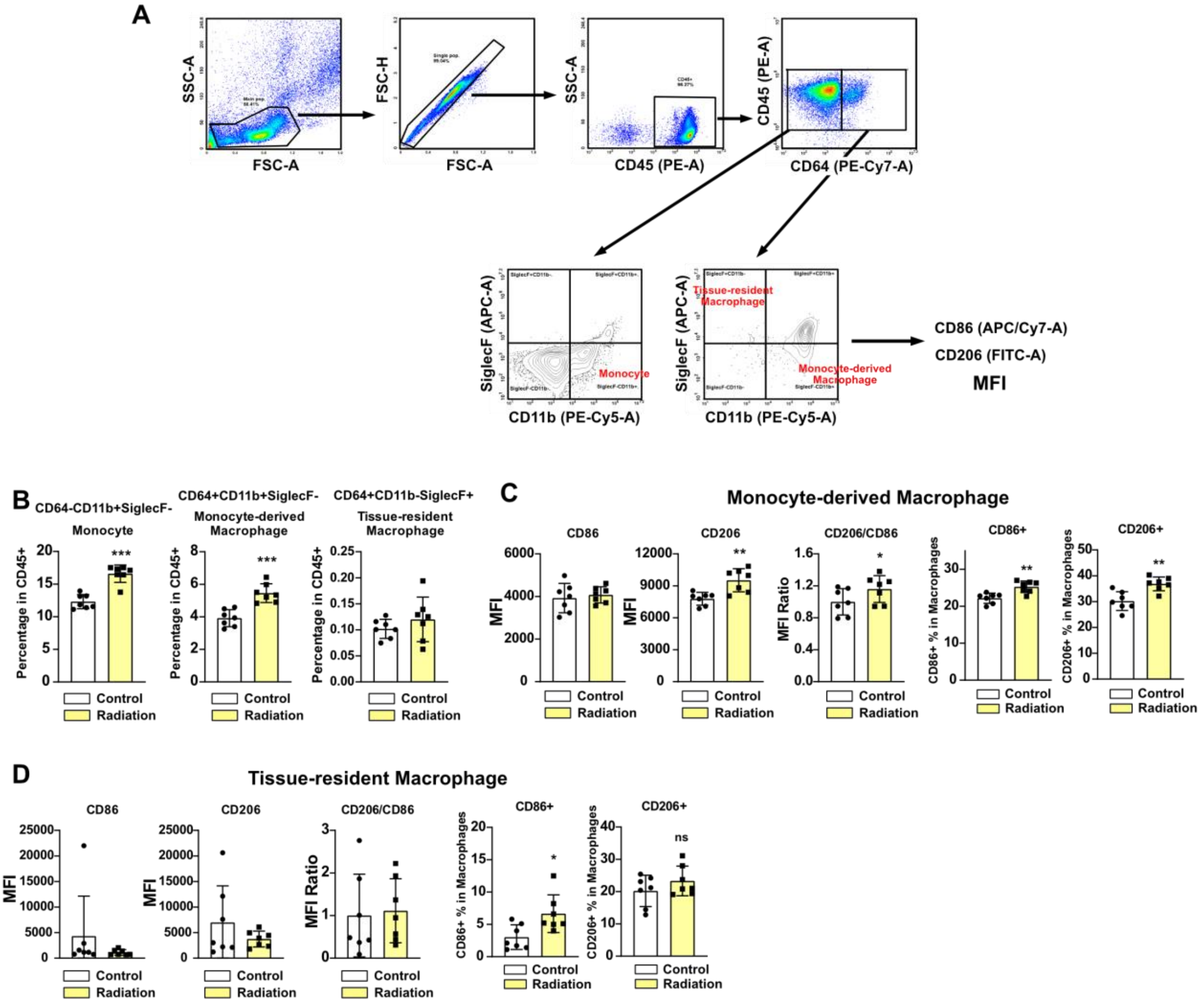


**Figure S3. Radiation-induced changes of monocyte-derived macrophages and tissue-resident macrophages in mouse lung tissues**

Changes in immune cells infiltration in lung tissues. Lung tissues were collected 2 weeks after radiation (22 Gy×2). *n* = 6/group. (**A**) Gating strategy in flow cytometry. The used flow cytometry antibodies are listed in Table S3. (**B**) Percentage of monocytes (CD45+CD64-CD11b+SiglecF-), monocyte-derived macrophages (CD45+CD64+CD11b+SiglecF-), and tissue-resident macrophages (CD45+CD64+CD11b-SiglecF+) after radiation. (**C**) Radiation-induced M2 differentiation of monocyte-derived macrophages. (**D**) Radiation-induced M2 differentiation of tissue-resident macrophages. All data were presented as mean ± SD. Statistical significance of the differences was determined by two-tailed Student t-test.

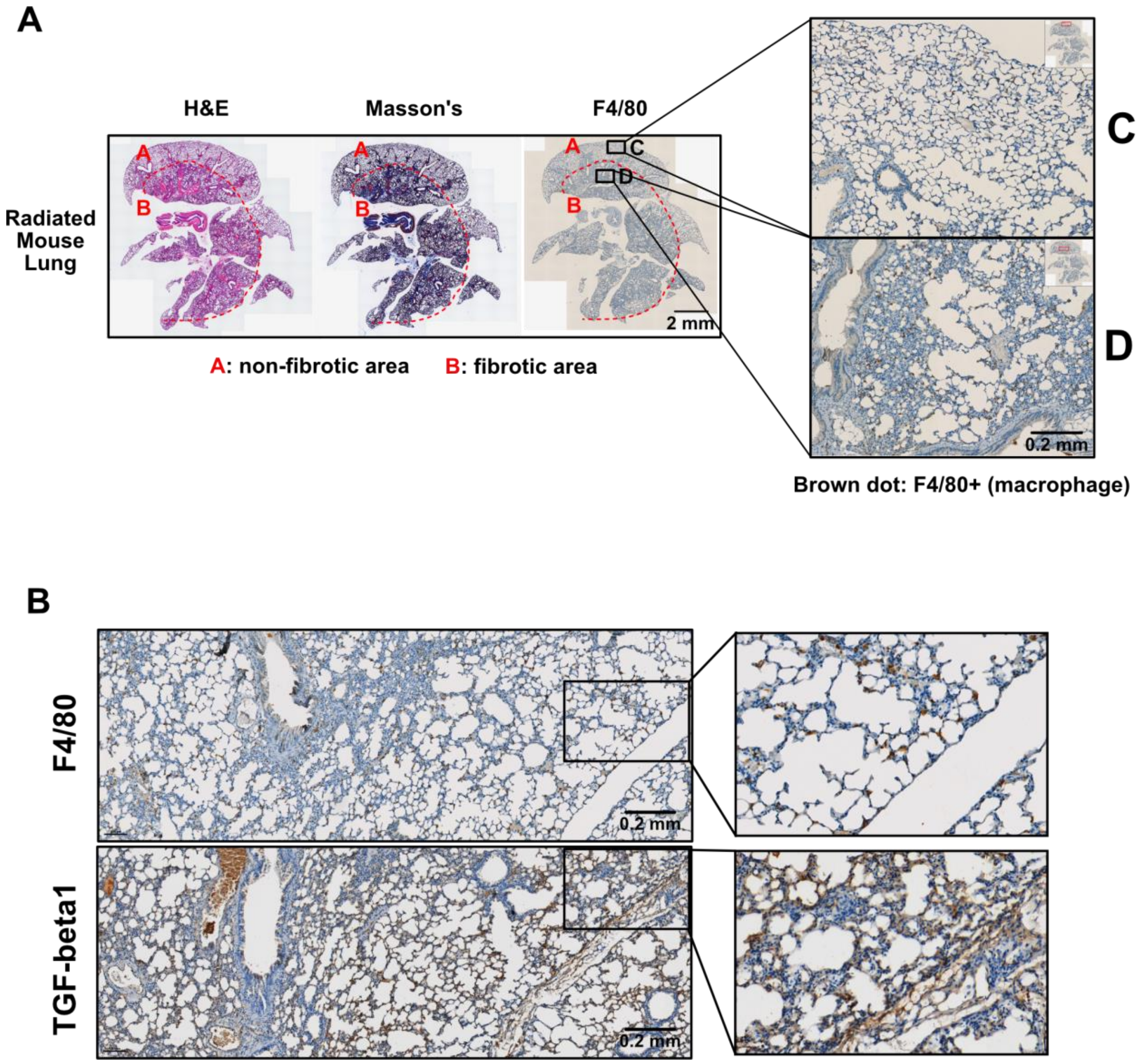


**Figure S4. F4/80 and TGF-β1 immunohistochemistry staining at the irradiated site**

(**A**) Comparison of F4/80+ macrophage infiltration into irradiated and non-irradiated sites. (**B**) Co-localization of F4/80+ macrophages and TGF-β1. The used IHC antibody is listed in Table S3.

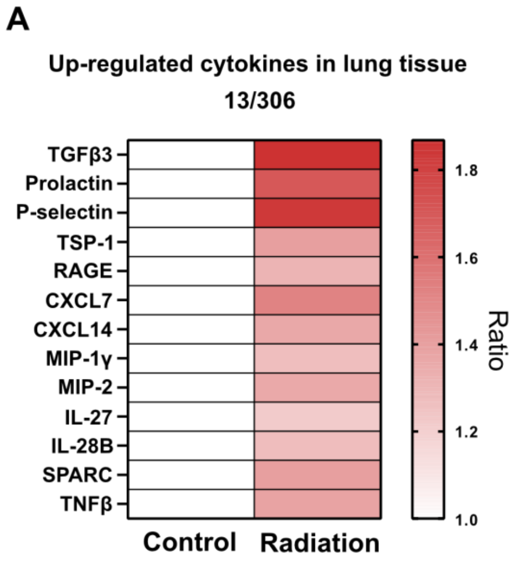


**Figure S5. Identification of other cytokines increased in irradiated lung tissue**

(**A**) Increased cytokines in irradiated lung tissues were represented. The mouse chest underwent a single irradiation with 22 Gy, and the mouse lungs were dissected three days later. Isolated lung tissues were processed according to the cytokine array manufacturer's manual.

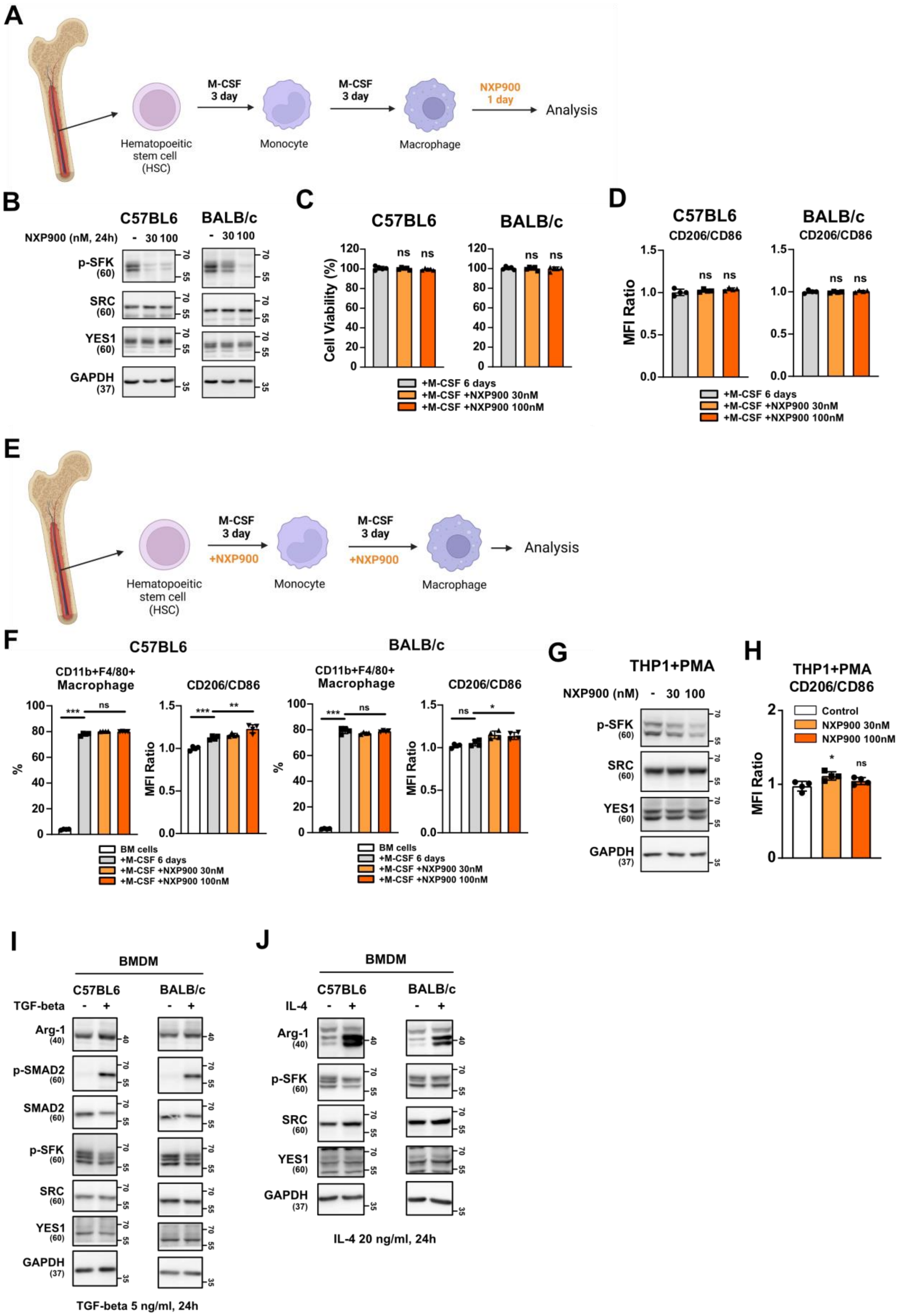
A
M-CSF 3 day
M-CSF 3 day
NXP900 1 day
Analysis
Hematopoeitic stem cell (HSC)
Monocyte
Macrophage
B
C57BL6
BALB/c
NXP900 (nM, 24h)
p-SFK (60)
SRC (60)
YES1 (60)
GAPDH (37)
C
Cell Viability (%)
+M-CSF 6 days
+M-CSF +NXP900 30nM
+M-CSF +NXP900 100nM
D
CD206/CD86
MFI Ratio
E
+NXP900
F
CD11b+F4/80+ Macrophage
BM cells
G
THP1+PMA
NXP900 (nM)
H
THP1+PMA CD206/CD86
Control
NXP900 30nM
NXP900 100nM
I
BMDM
TGF-beta
Arg-1 (40)
p-SMAD2 (60)
SMAD2 (60)
TGF-beta 5 ng/ml, 24h
J
IL-4
IL-4 20 ng/ml, 24h

**Figure S6. Effect of SFK-targeted inhibitor on macrophage**

(**A**) Schematic illustration of the experiment for Figure B-D. (**B**) Confirmation of SFK activity blockade in macrophages following NXP900 treatment. (**C**) Cell viability of macrophages treated with NXP900. (**D**) Effects of NXP900 on differentiated macrophages. (**E**) Schematic illustration of the experiment for Figure F. (**F**) Effects of NXP900 on differentiating macrophages. (**G, H**) Effects of NXP900 on THP1 human macrophages. (**I**) Confirmation of SFK activity during M2 differentiation induced by TGF-β treatment. (**J**) Confirmation of SFK activity during M2 differentiation induced by IL-4 treatment. All data were presented as mean ± SD. Statistical significance of the differences was determined by two-tailed Student t-test or one-way ANOVA followed by the Tukey's test. All western blot analyses in this study were repeated three times independently.

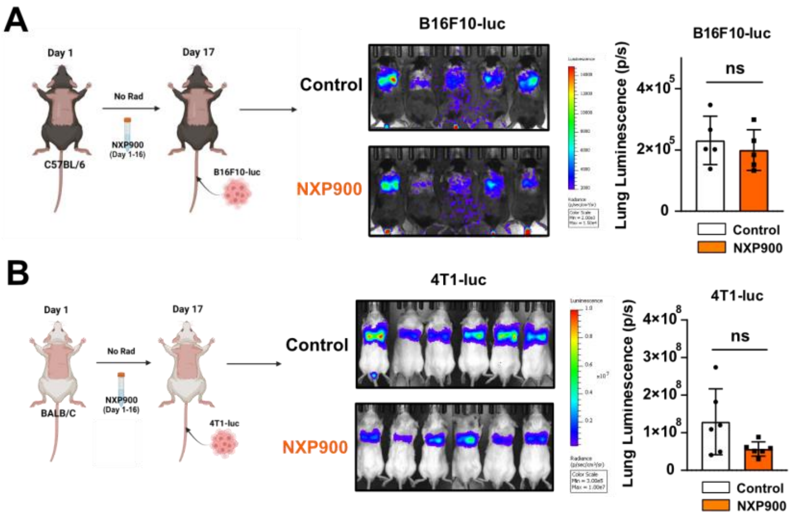


**Figure S7. Inhibition of lung engraftment by residual drug**

(**A, B**) NXP900 (60 mg/kg, daily, PO) was administered from day 1 to 16. One day after stopping the drug, the cancer cells were injected into the tail vein. $n$ = 5 or 6/group. All data were presented as mean ± SD. Statistical significance of the differences was determined by two-tailed Student t-test

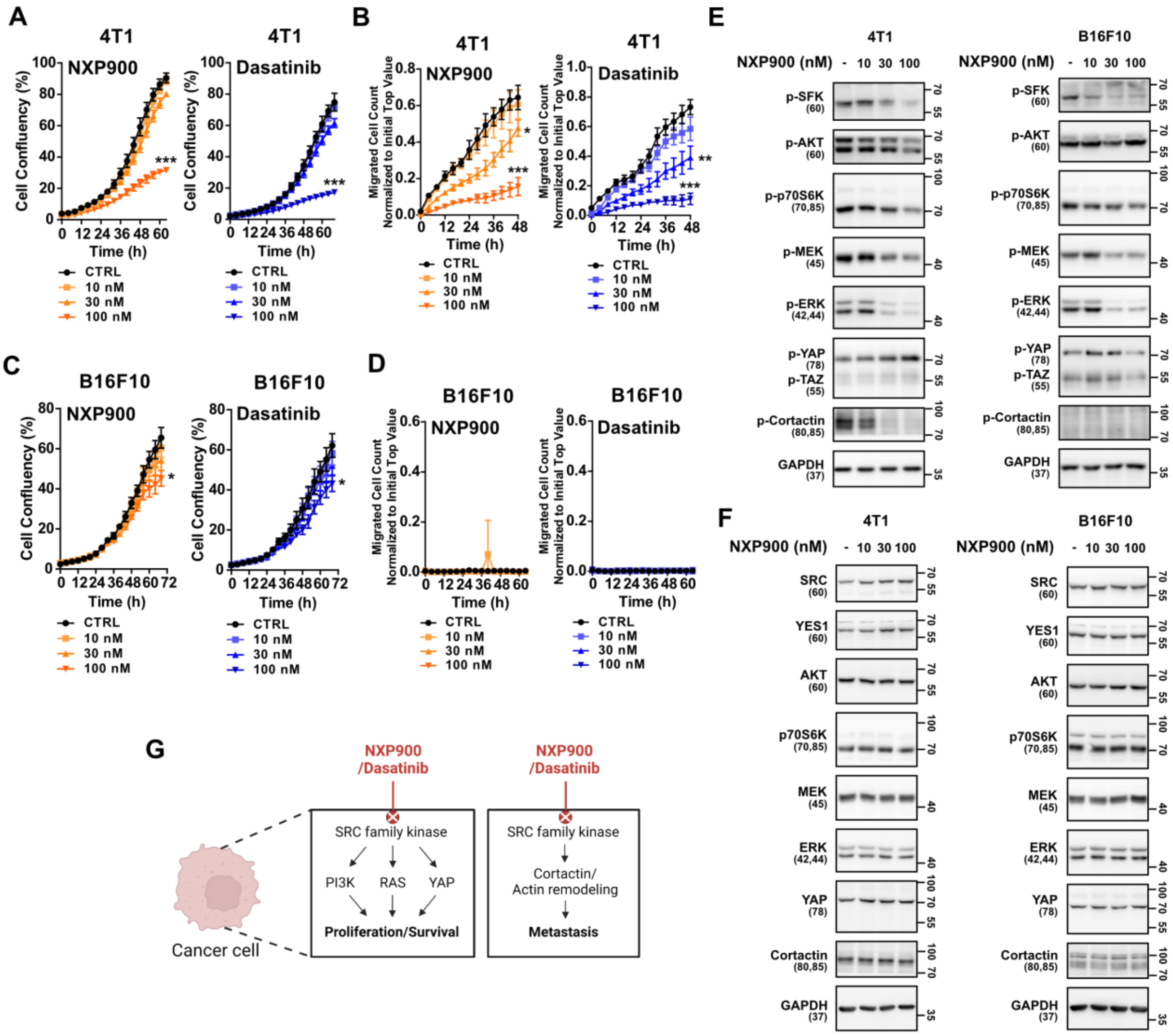


**Figure S8. Direct effect of SFK-targeted inhibitors on cancer cells**

(**A**) Anti-proliferative activities of SFK-targeted inhibitors in 4T1 mouse breast cancer cells. (**B**) Anti-migratory activities of SFK-targeted inhibitors in 4T1 cells. (**C**) Anti-proliferative activities of SFK-targeted inhibitors in B16F10 mouse melanoma cells. (**D**) Anti-migratory activities of SFK-inhibitors in B16F10 cells. (**E, F**) Inhibition of SFKs downstream pathways by SFK-targeted inhibitors in mouse cancer cells. (**G**) Schematic illustration representing this figure. All data were presented as mean ± SD. Statistical significance of the differences was determined by one-way ANOVA followed by the Tukey's test. All western blot analyses in this study were repeated three times independently.

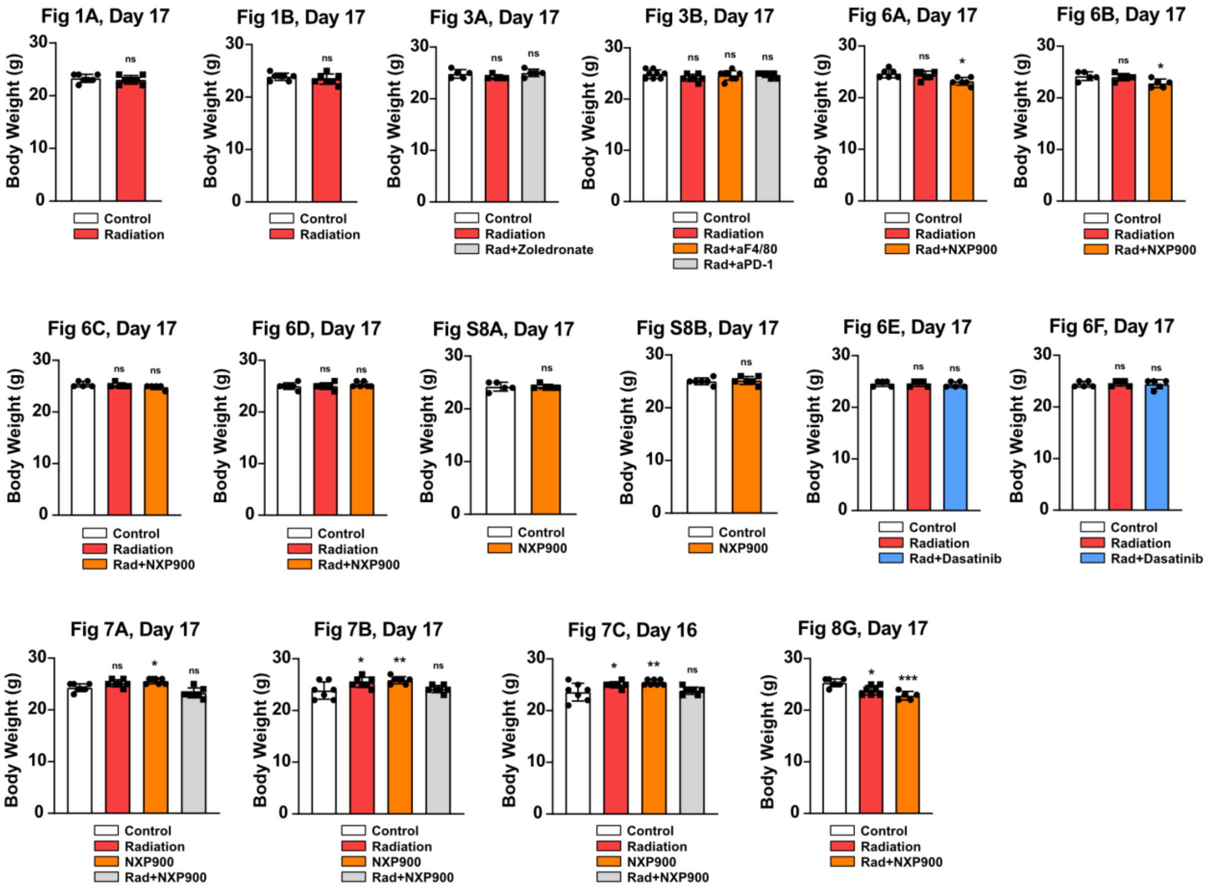


**Figure S9. Body weight changes in animal experiments**

In all animal experiments, body weight changes are presented. The corresponding figure number is entered for each graph. All data were presented as mean (SD). Statistical significance of the differences was determined by two-tailed Student t-test or one-way ANOVA followed by the Tukey's test.

Figure 1D

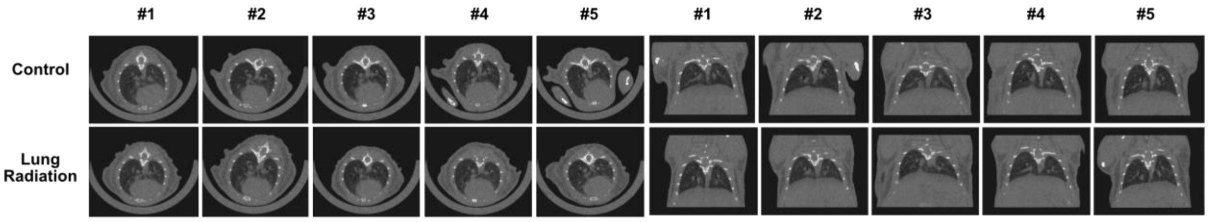


Figure 1F

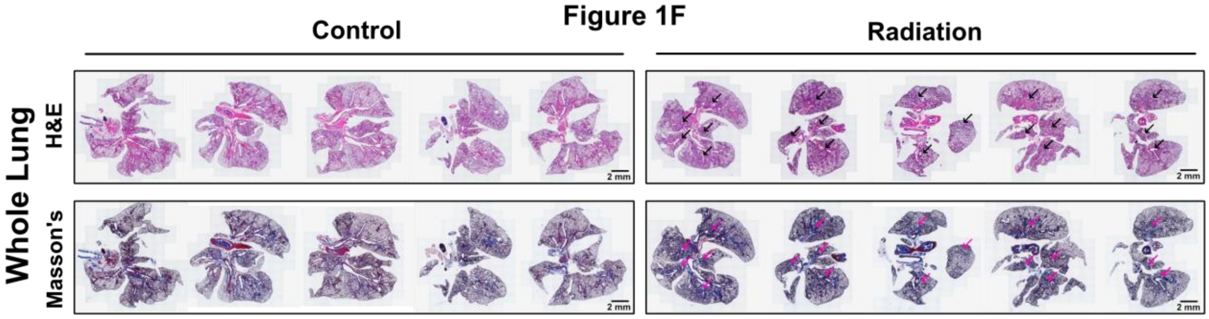


Figure 6G

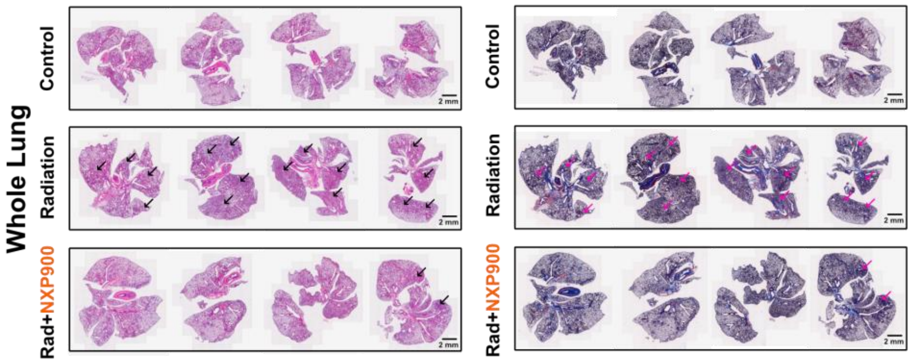


Figure 9B

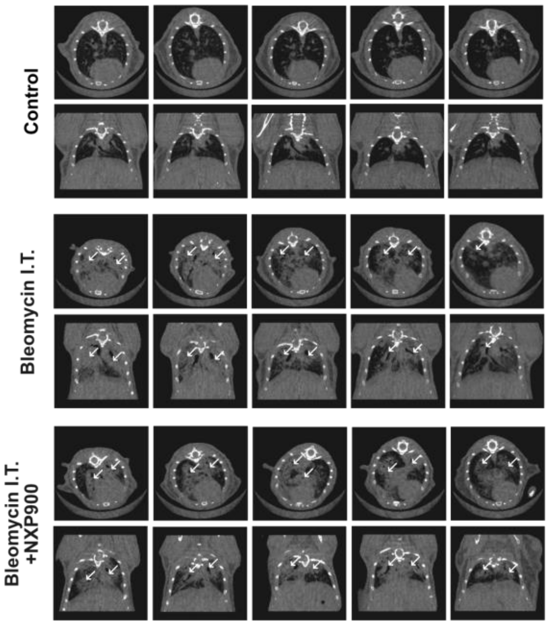


Figure 9E

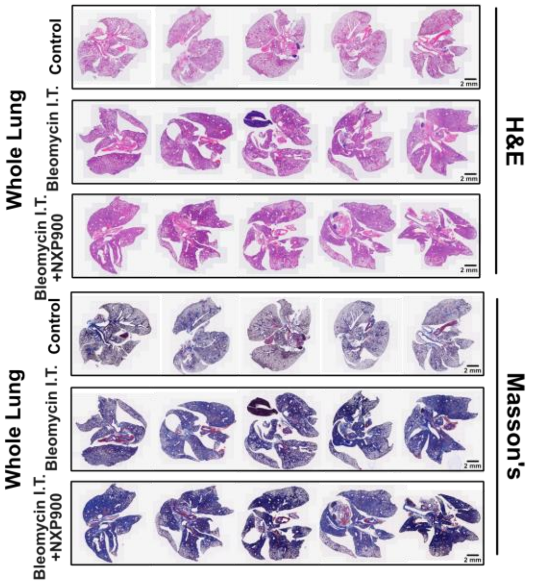

**Figure S10. Individual images of tissue staining and micro-CT from all animal experiments**

Individual images of tissue staining and micro-CT from all animal experiments are presented. The corresponding figure number is indicated for each image. Arrows in each image highlight areas of alveolar collapse (H&E staining) and collagen deposition (Masson's trichrome staining).

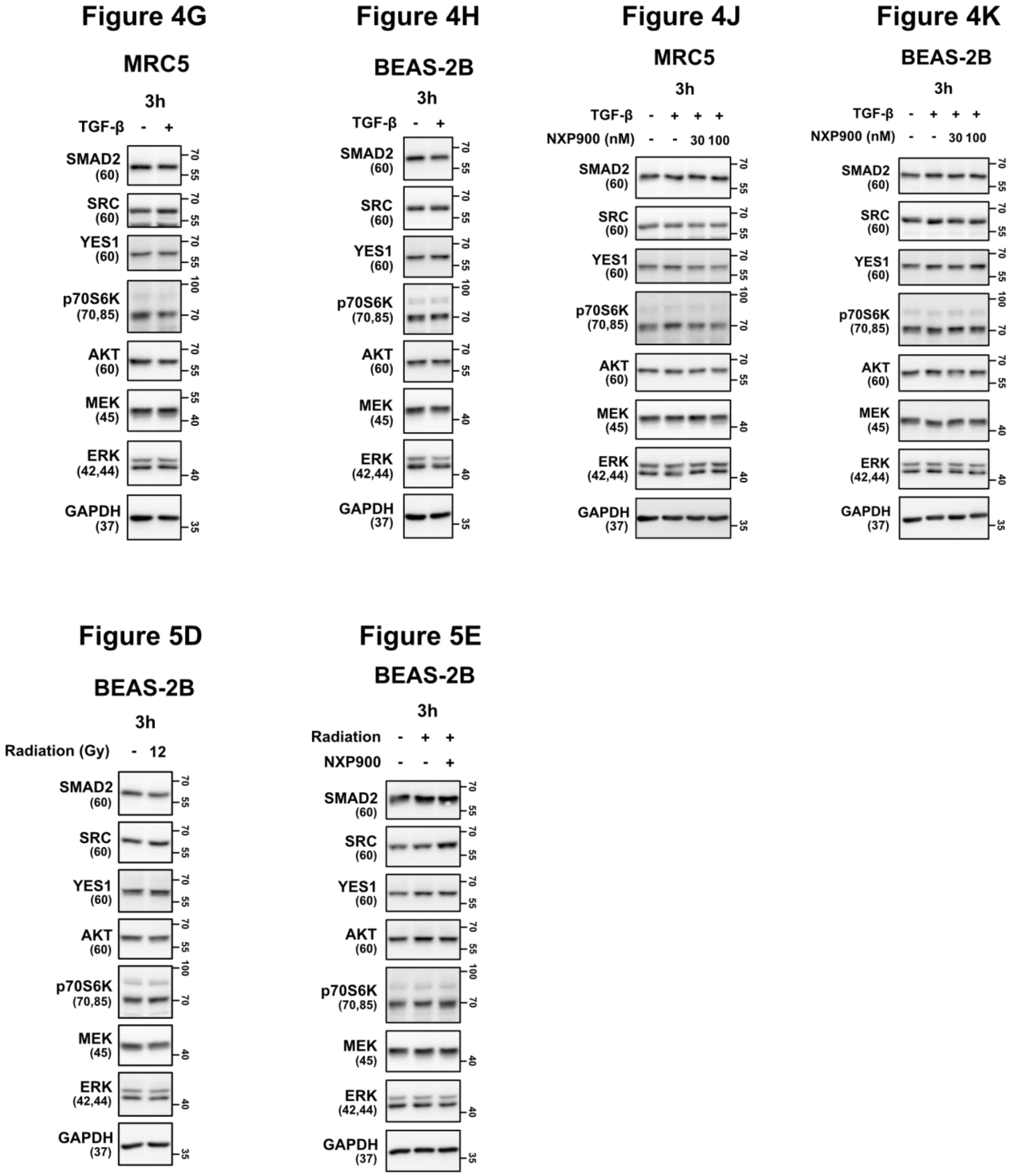


**Figure S11. Total protein levels for Figure 4 and 5**

Total protein levels are presented for Figure 4 and 5, with the corresponding figure numbers indicated for each graph. All western blot analyses in this study were performed independently in triplicate.
