# Supplementary material for: Prevention of radiotherapy-induced pro-tumorigenic microenvironment by SFK inhibitors
Source: Theranostics. 2025 Jan 1;15(3):875–93. doi: 10.7150/thno.100970 (PMC11700852; doi:10.7150/thno.100970)

Fig. 2J

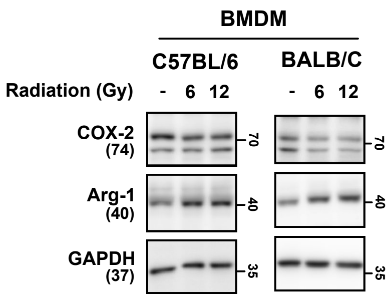

5 Biological Replicates

C57BL/6

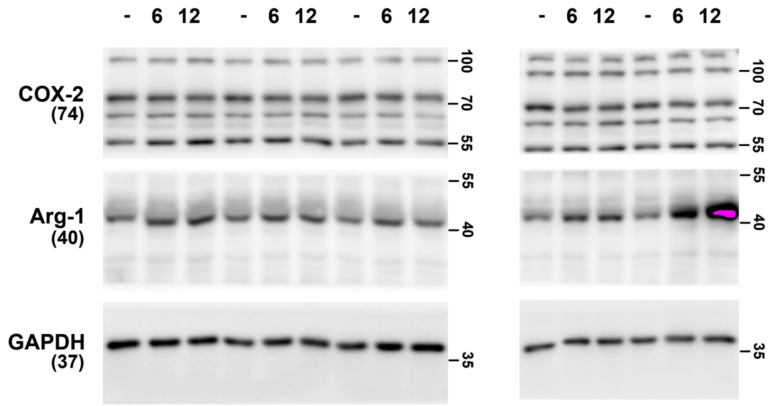

3 Biological Replicates

BALB/C

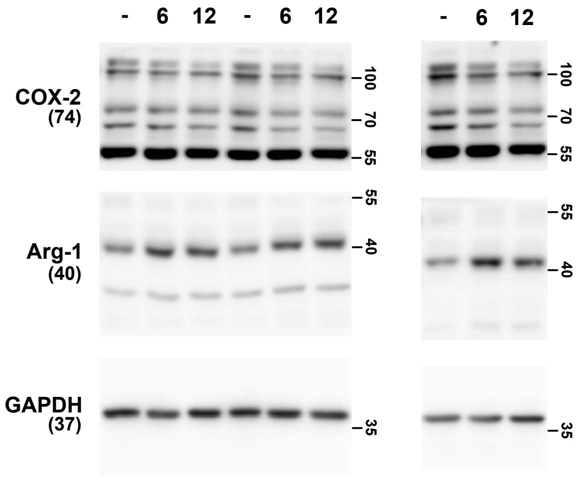

Fig. 4E

Mouse Lung Tissue

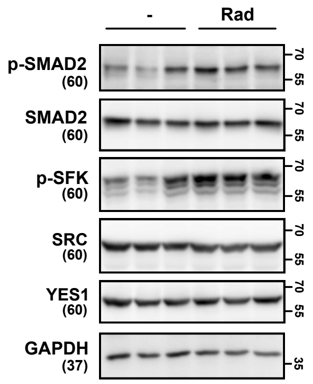

3 Different Mouse Tissue

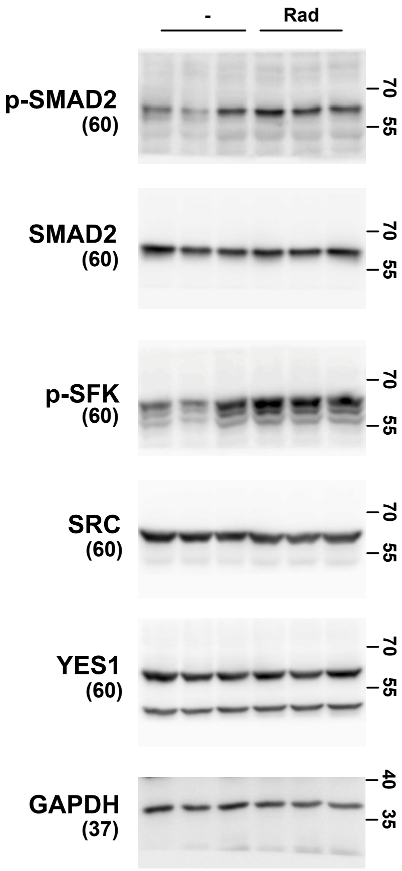

Fig. 4F

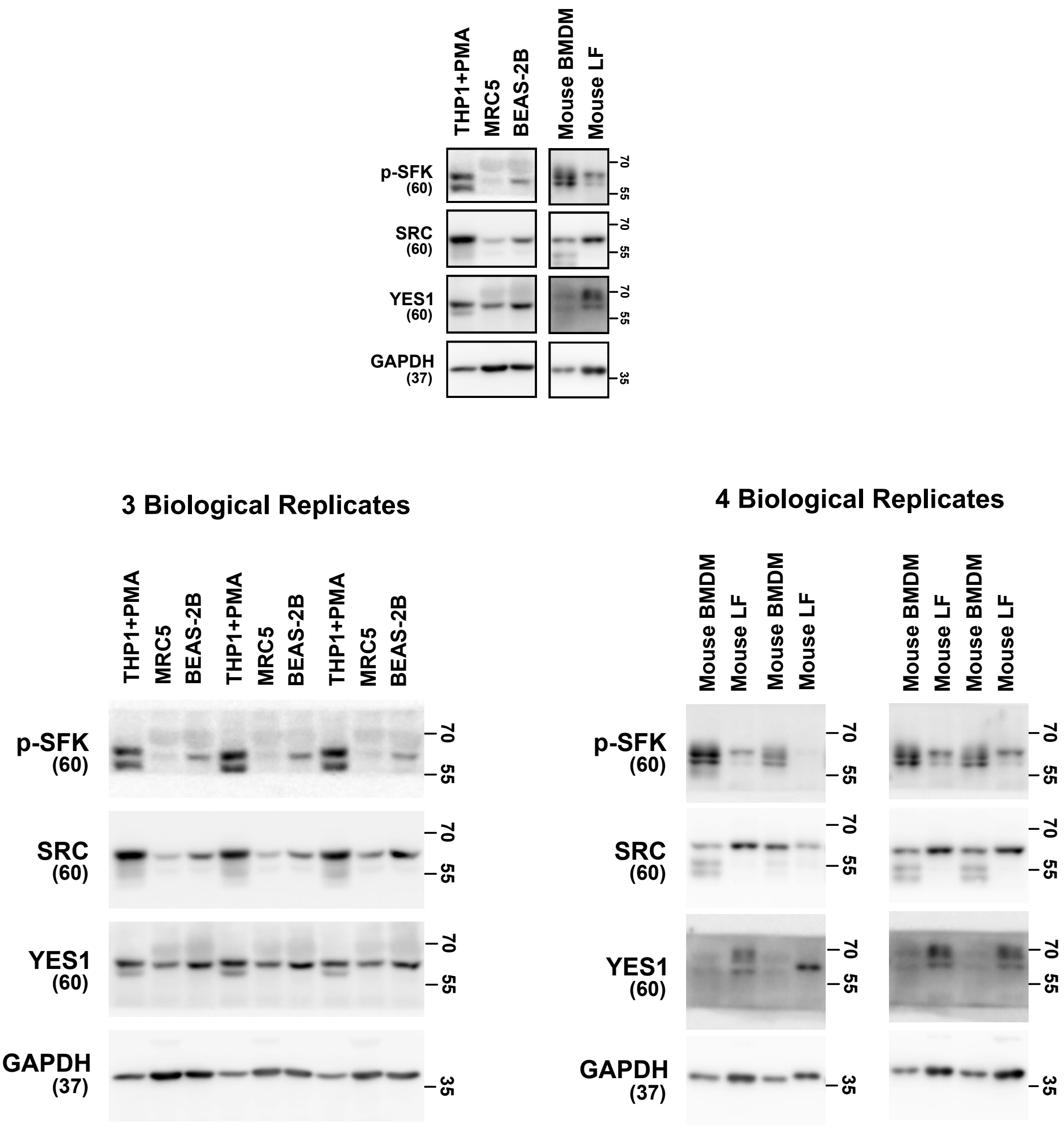
THP1+PMA
MRC5
BEAS-2B
Mouse BMDM
Mouse LF
p-SFK (60)
SRC (60)
YES1 (60)
GAPDH (37)
70
55
35
3 Biological Replicates
4 Biological Replicates

Fig. 4G **Supplementary Fig. 11**

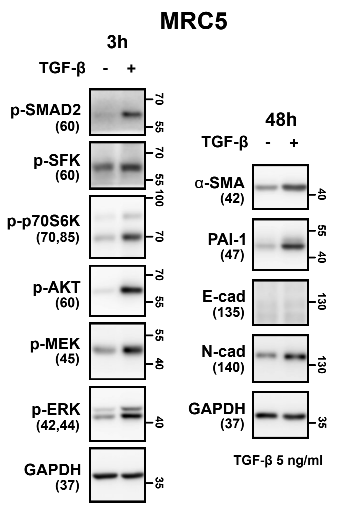

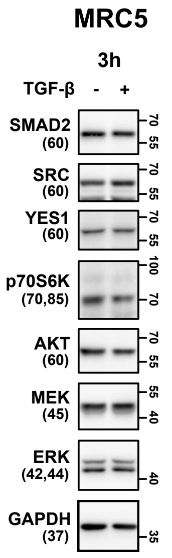

5 Biological Replicates

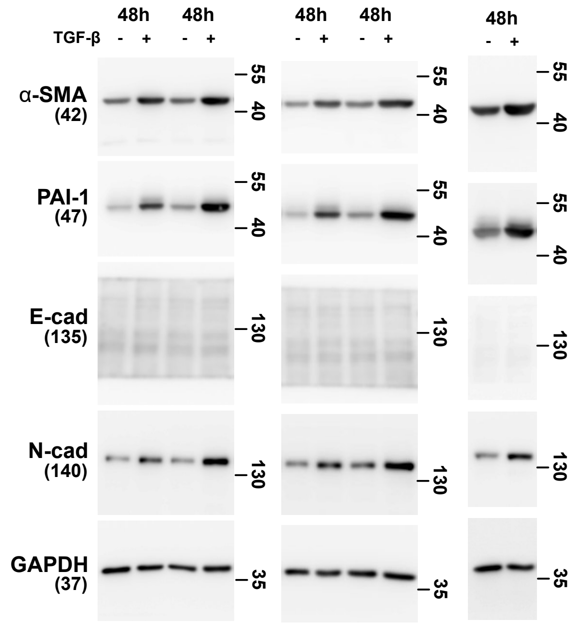

3 Biological Replicates

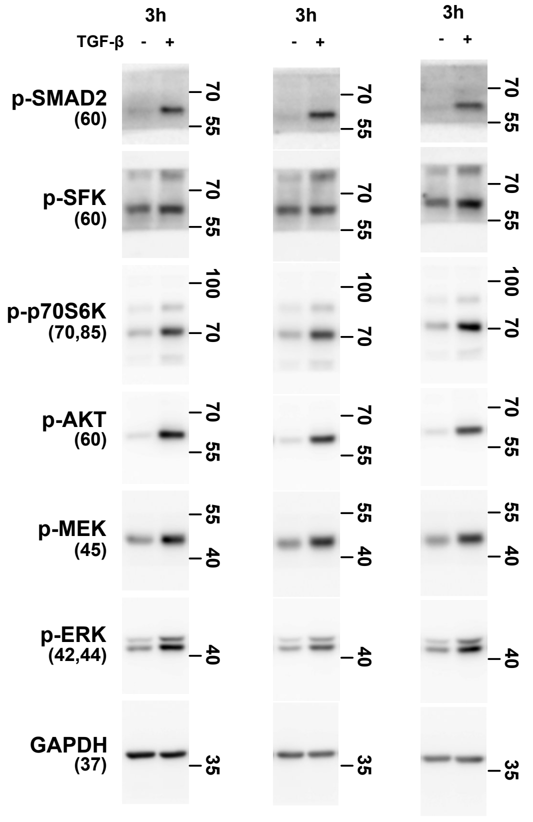

4 Biological Replicates

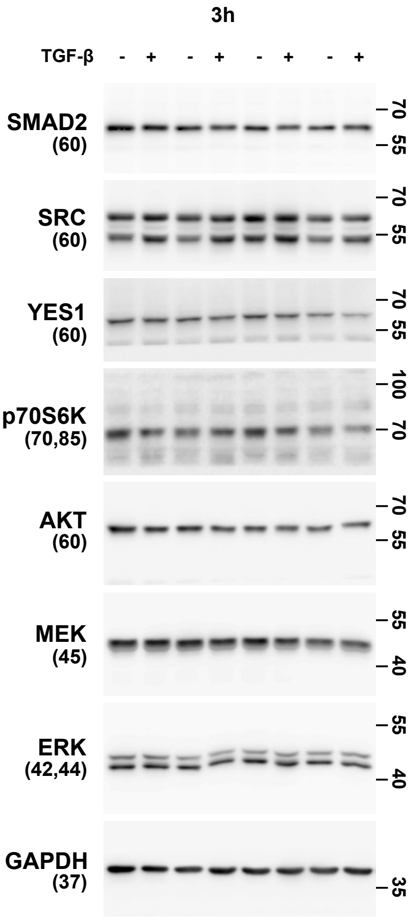

Fig. 4H

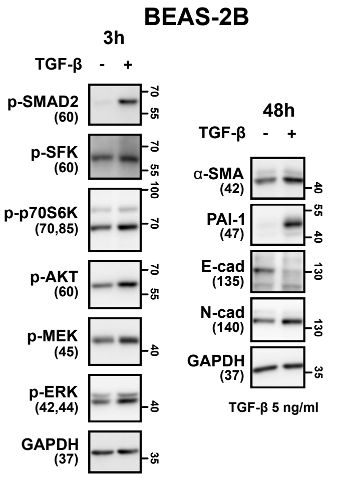

Supplementary Fig. 11

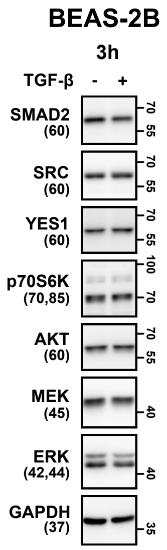

3 Biological Replicates

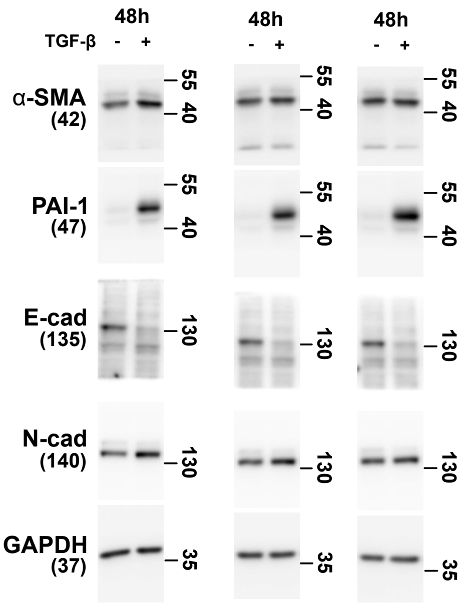

3 Biological Replicates

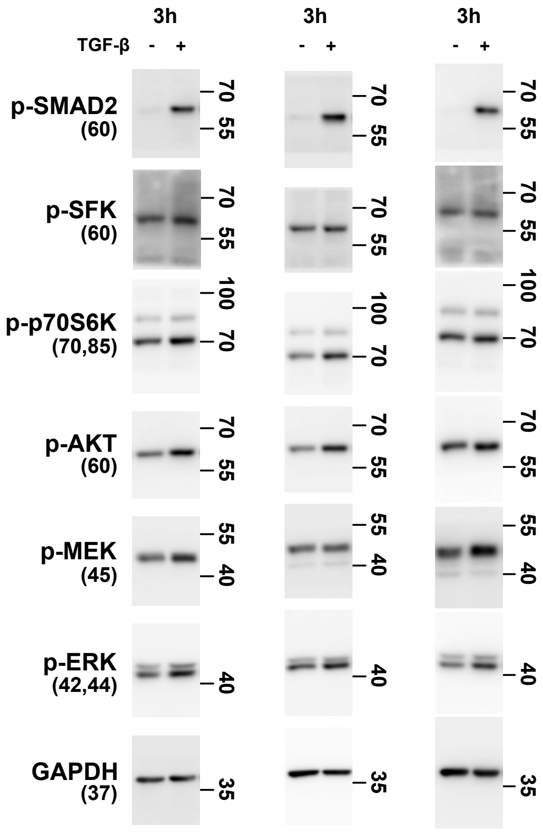

4 Biological Replicates

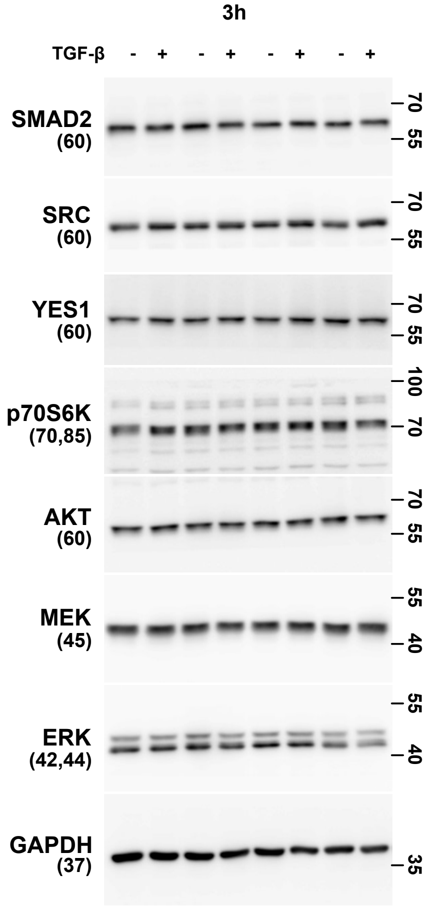

Fig. 4l

BEAS-2B

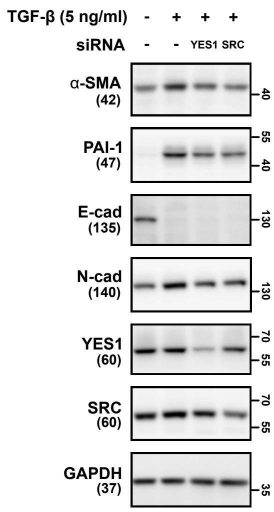

4 Biological Replicates

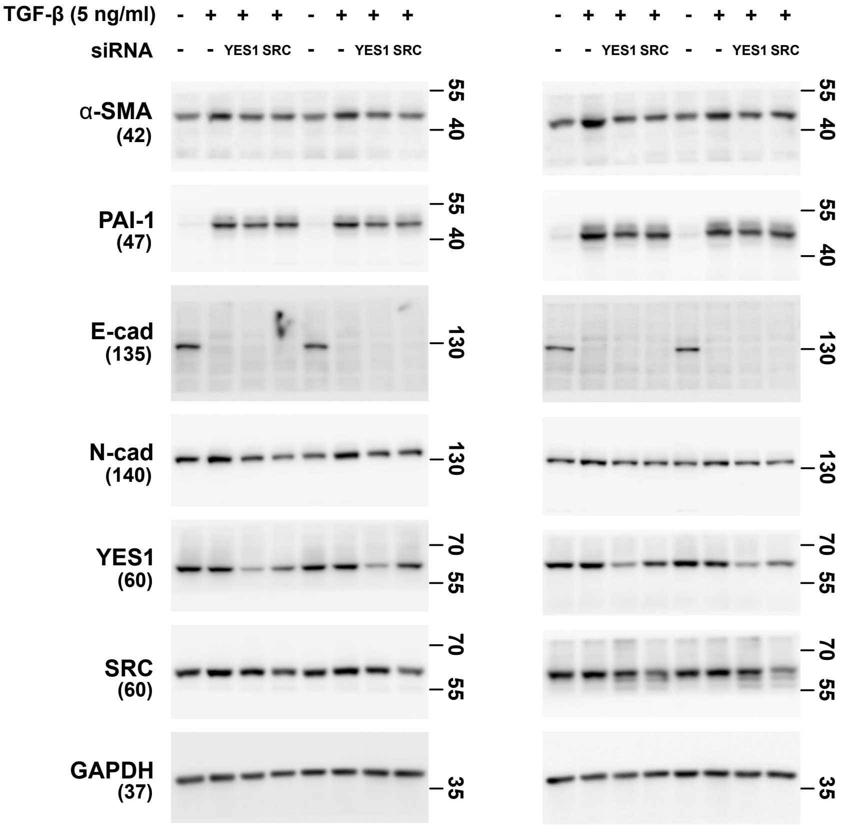

Fig. 4J

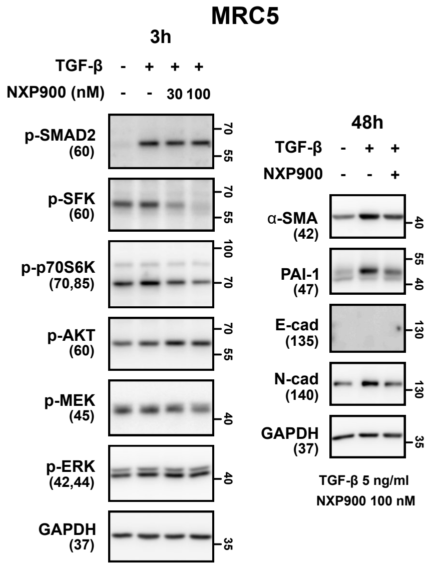

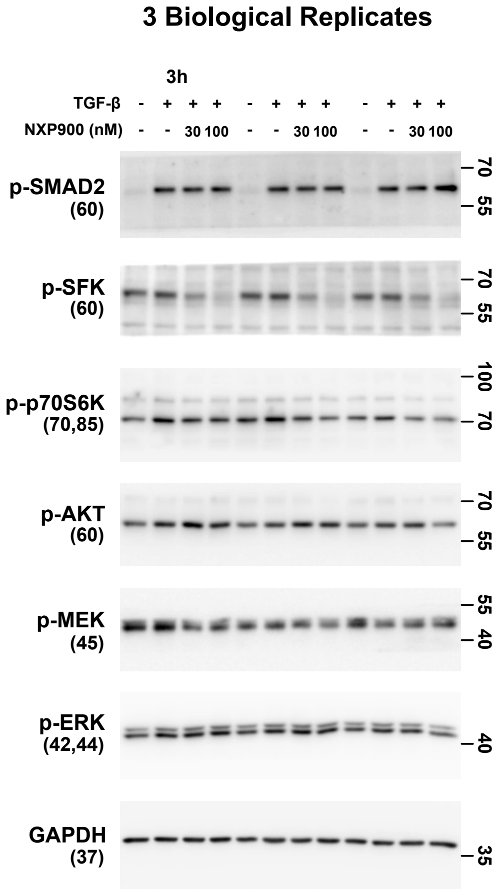

Supplementary Fig. 11

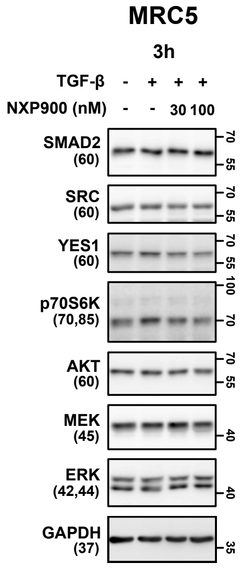

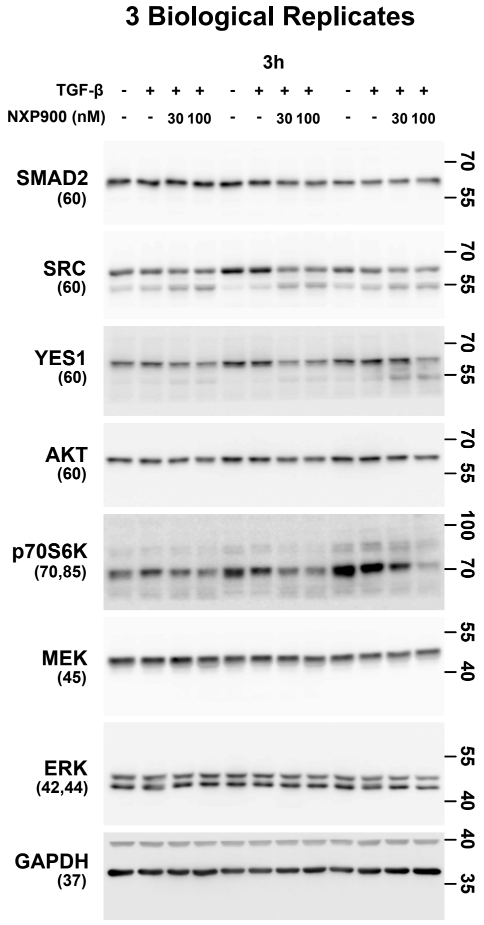

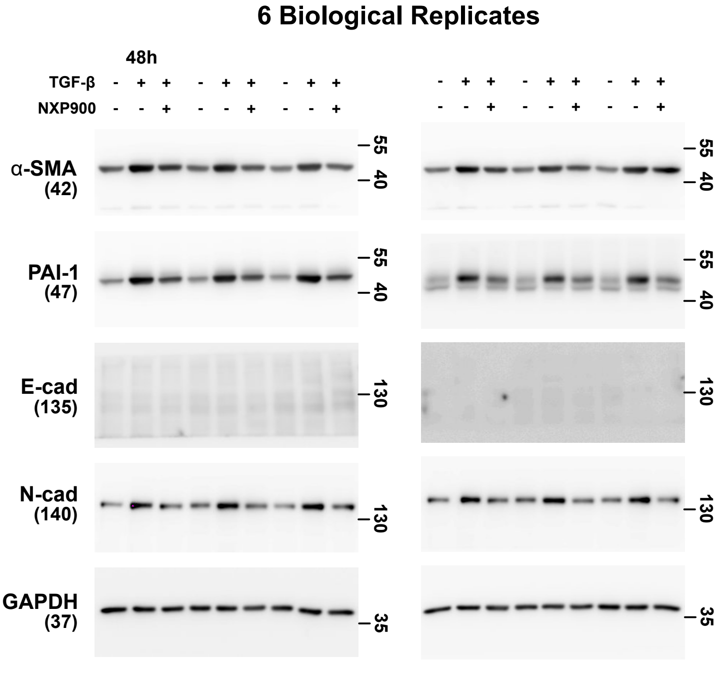

Fig. 4K

Supplementary Fig. 11

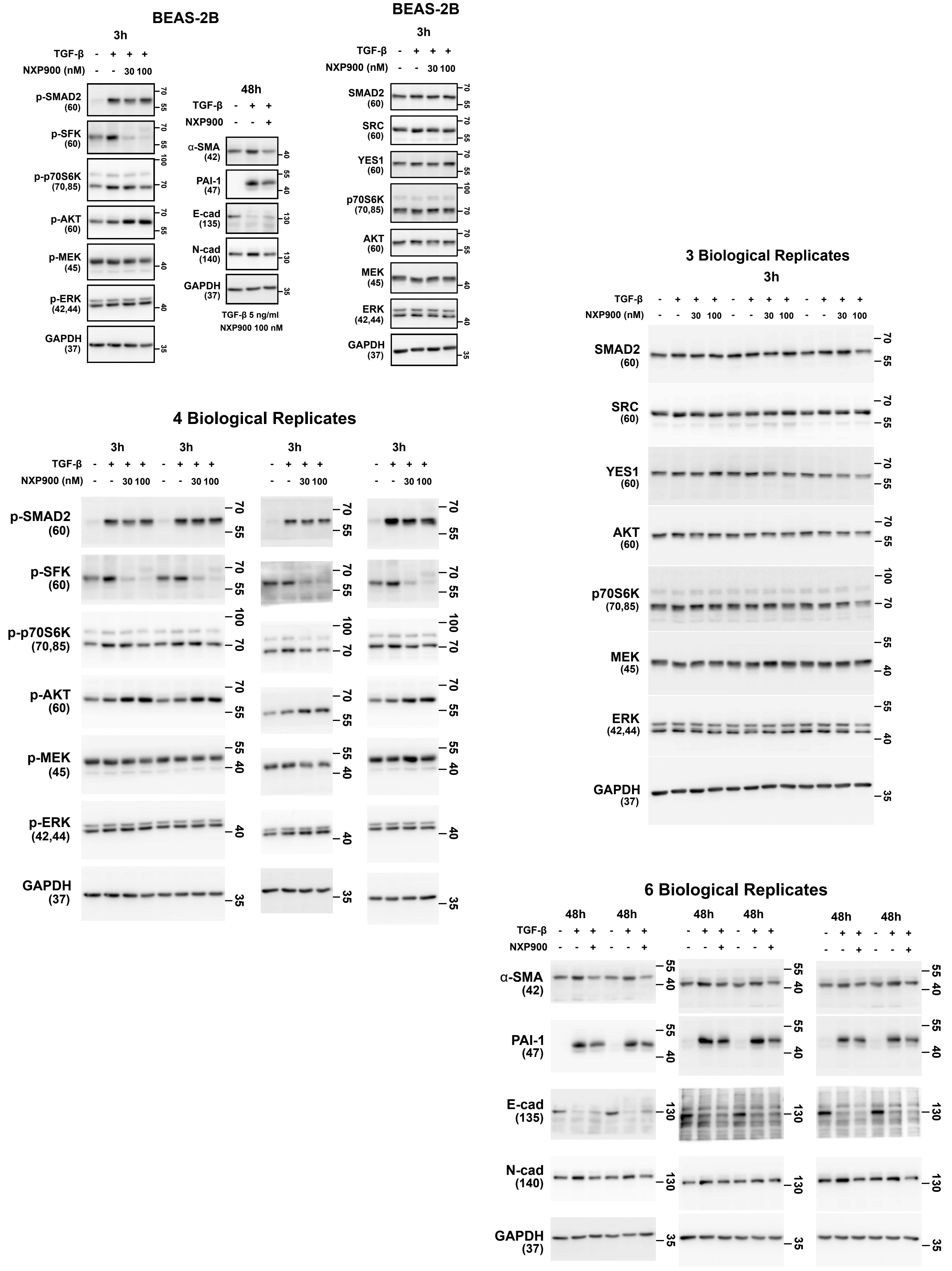

Fig. 4L

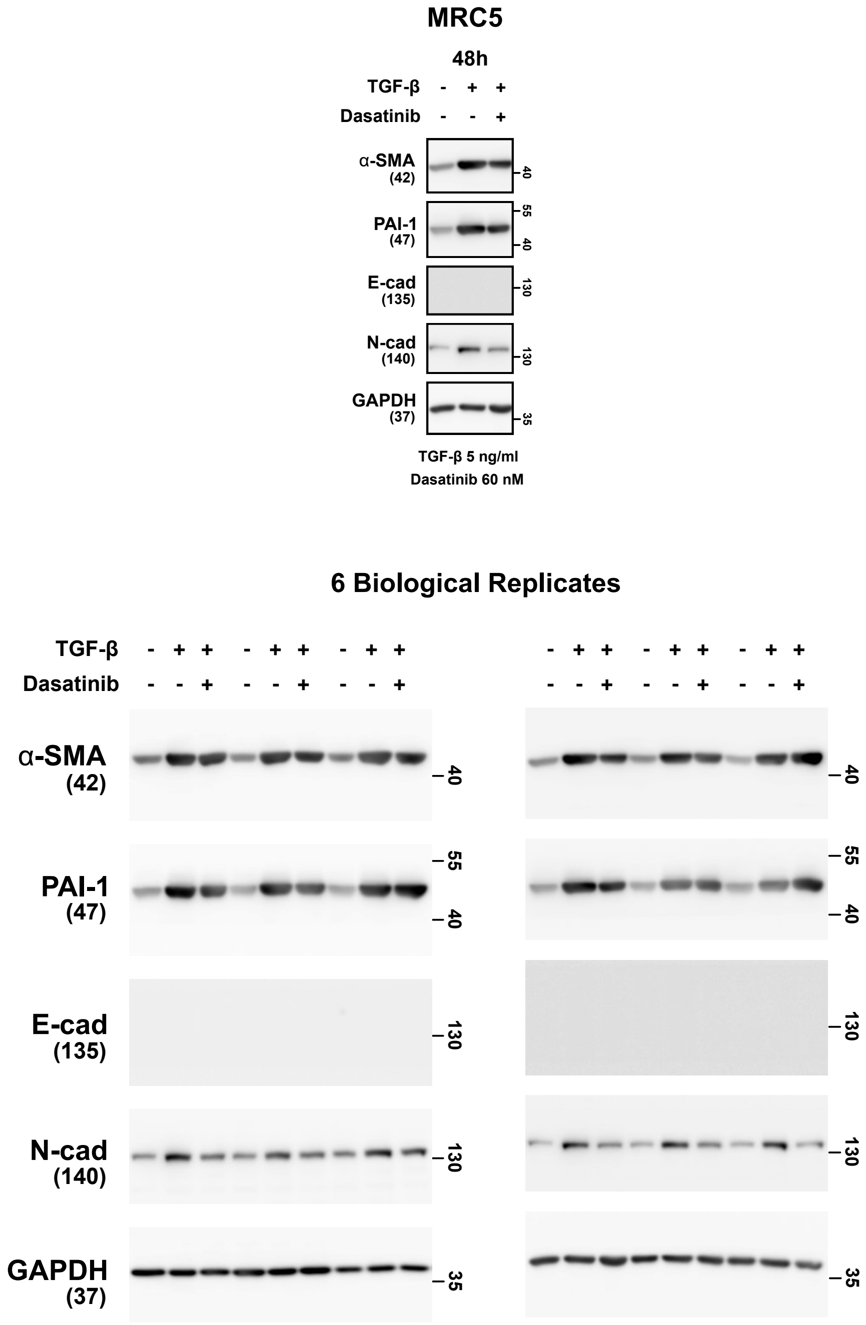

MRC5
48h
TGF-β - + +
Dasatinib - - +
α-SMA (42)
40
PAI-1 (47)
55
40
E-cad (135)
130
N-cad (140)
130
GAPDH (37)
35
TGF-β 5 ng/ml
Dasatinib 60 nM
6 Biological Replicates
TGF-β - + + - + + - + + - + + - + + - + +
Dasatinib - - + - - + - - + - - + - - + - - +
α-SMA (42)
PAI-1 (47)
E-cad (135)
N-cad (140)
GAPDH (37)

Fig. 4M

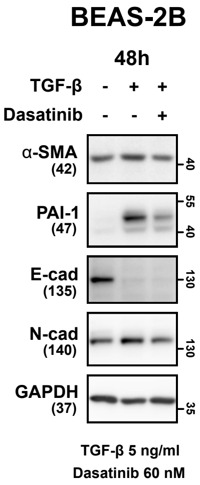
BEAS-2B
48h
TGF-β - + +
Dasatinib - - +
α-SMA (42)
40
PAI-1 (47)
55
40
E-cad (135)
130
N-cad (140)
130
GAPDH (37)
35
TGF-β 5 ng/ml
Dasatinib 60 nM

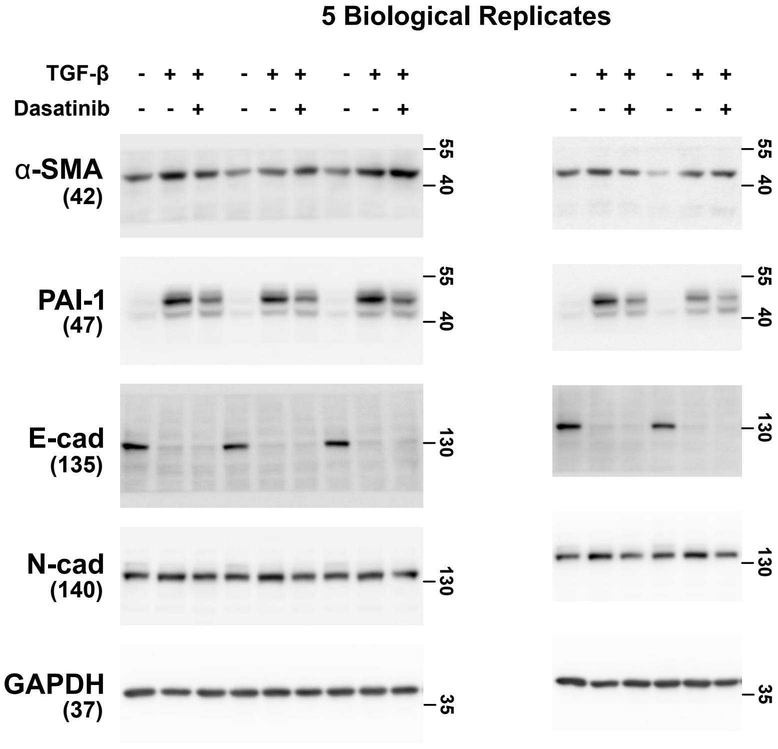
5 Biological Replicates
TGF-β - + + - + + - + +
Dasatinib - - + - - + - - +
TGF-β - + + - + +
Dasatinib - - + - - +
α-SMA (42)
55
40
PAI-1 (47)
55
40
E-cad (135)
130
N-cad (140)
130
GAPDH (37)
35

Fig. 5B

BEAS-2B

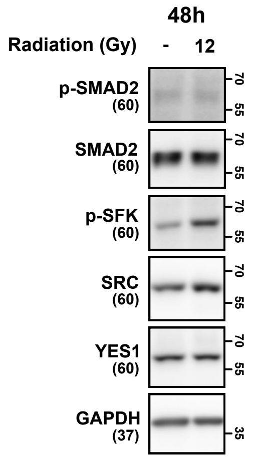

4 Biological Replicates

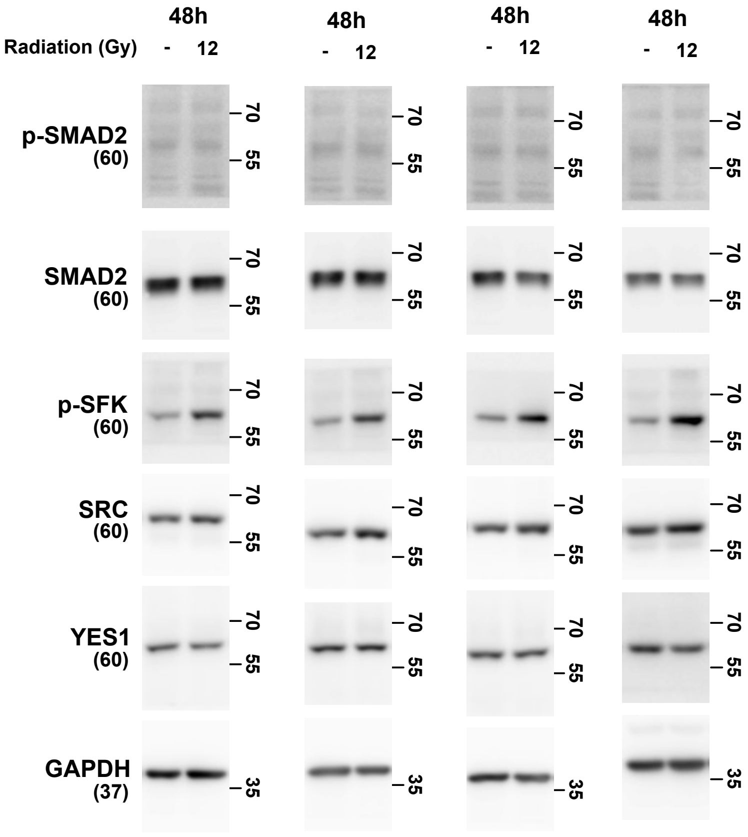

Fig. 5C

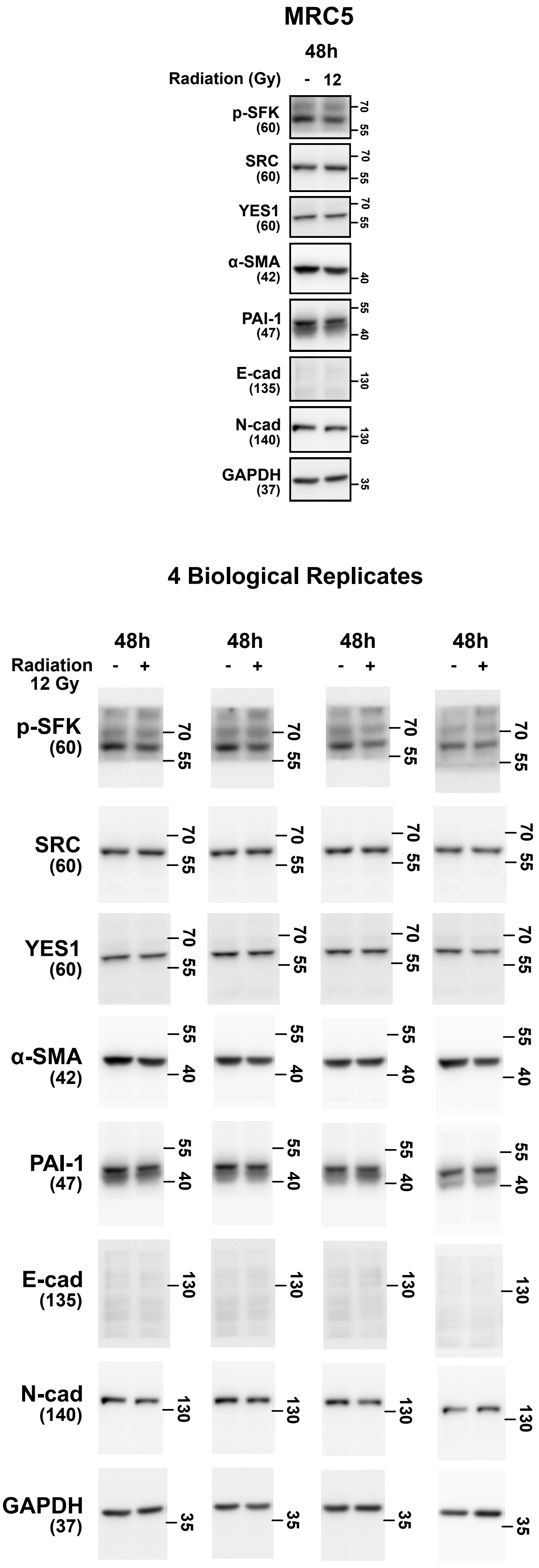

MRC5
48h
Radiation (Gy)
-
12
p-SFK (60)
SRC (60)
YES1 (60)
α-SMA (42)
PAI-1 (47)
E-cad (135)
N-cad (140)
GAPDH (37)
70
55
40
130
35
4 Biological Replicates
48h
Radiation 12 Gy
- +

Fig. 5D

BEAS-2B

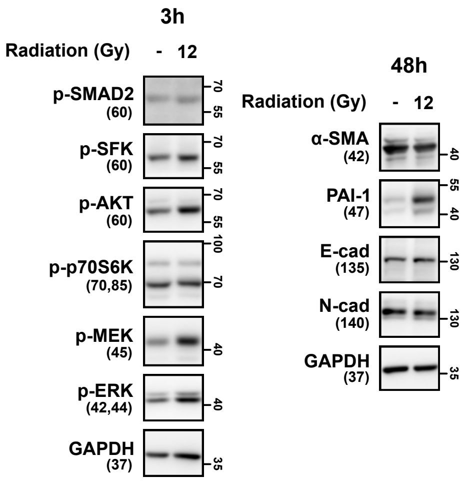

Supplementary Fig. 11

BEAS-2B

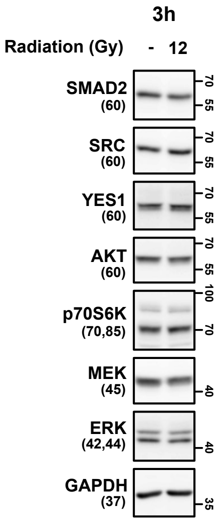

3 Biological Replicates

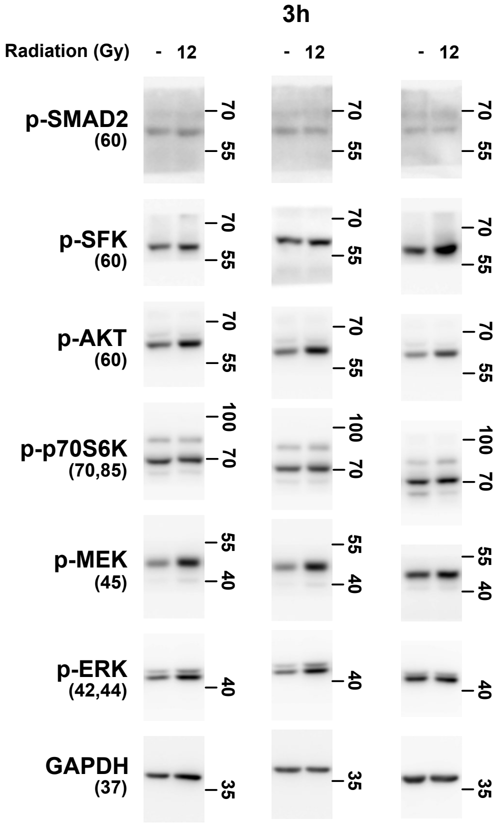

3 Biological Replicates

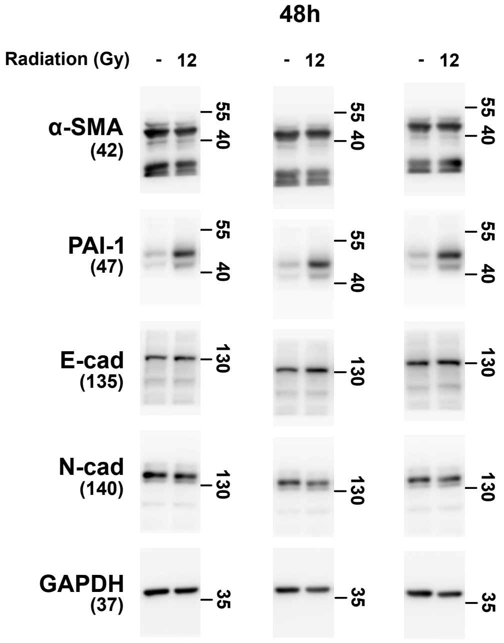

4 Biological Replicates

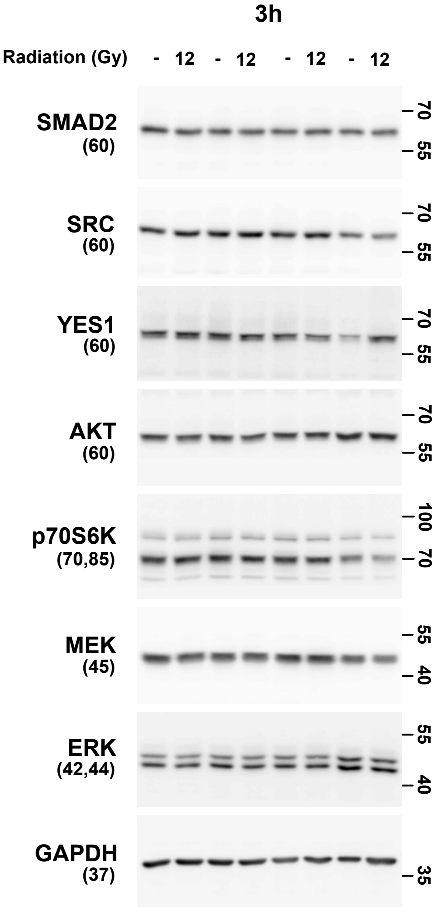

Fig. 5E

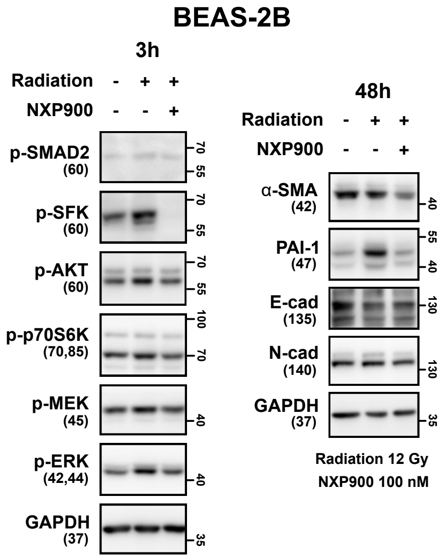

Supplementary Fig. 11

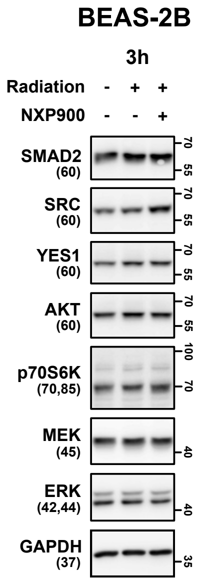

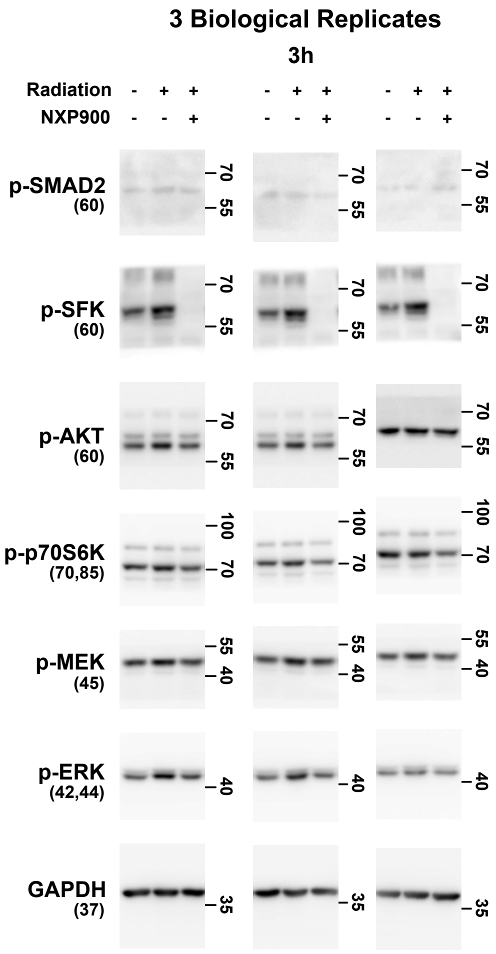

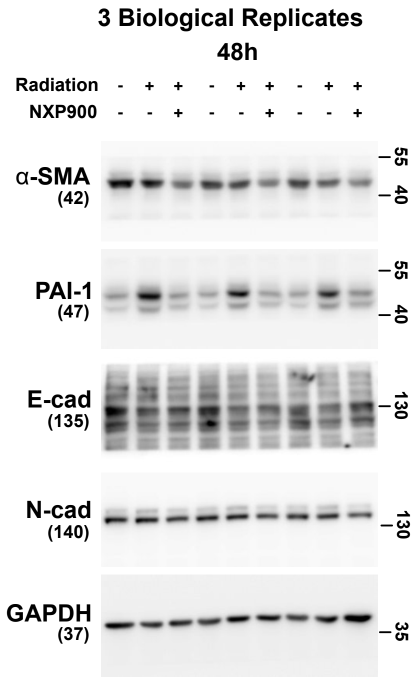

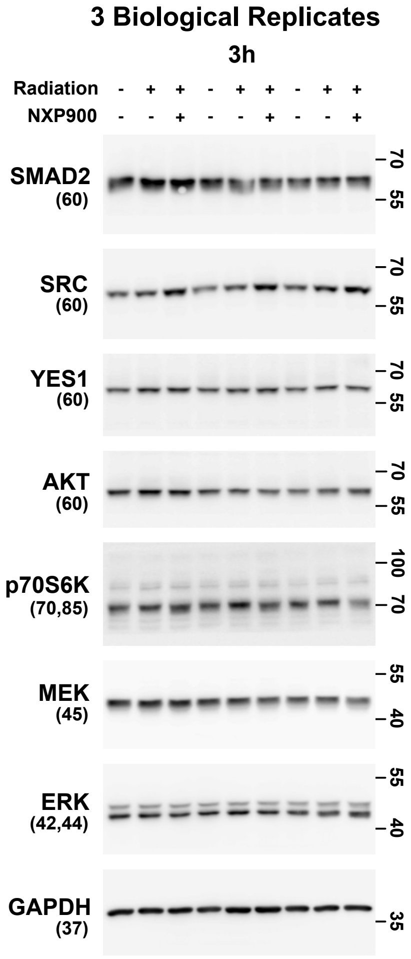

Fig. 5F

BEAS-2B

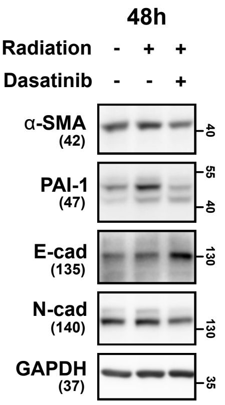

Radiation 12 Gy

Dasatinib 60 nM

6 Biological Replicates

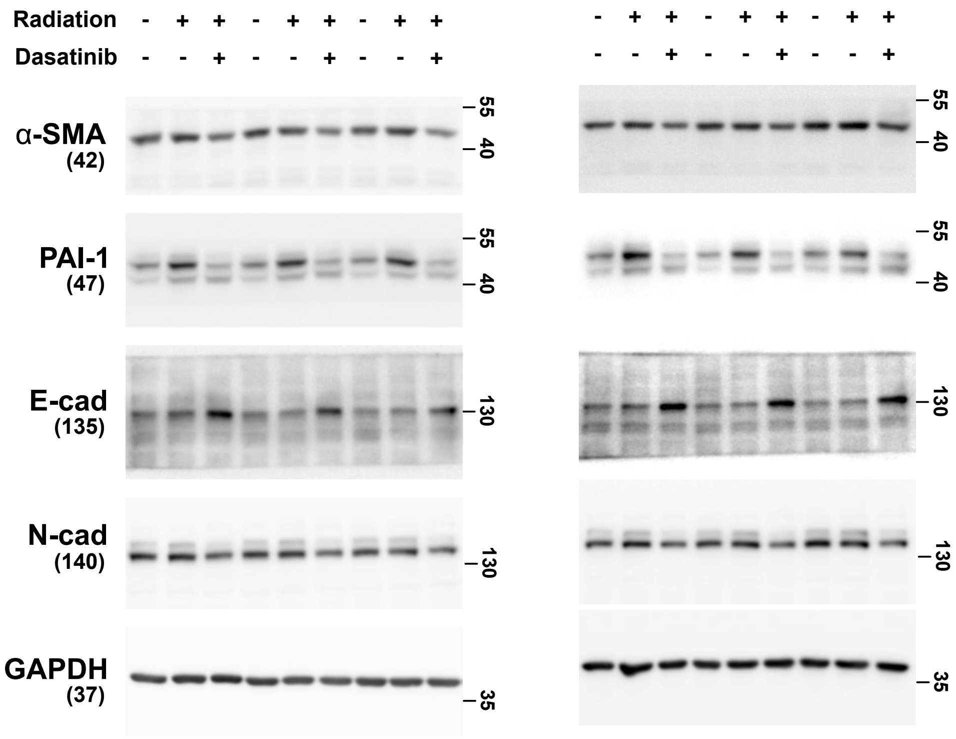

Fig. 7J

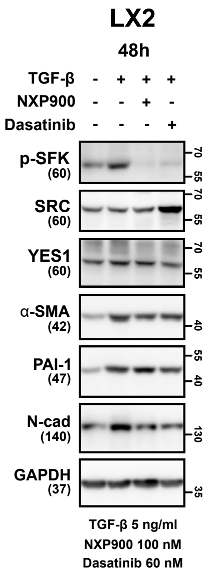

4 Biological Replicates

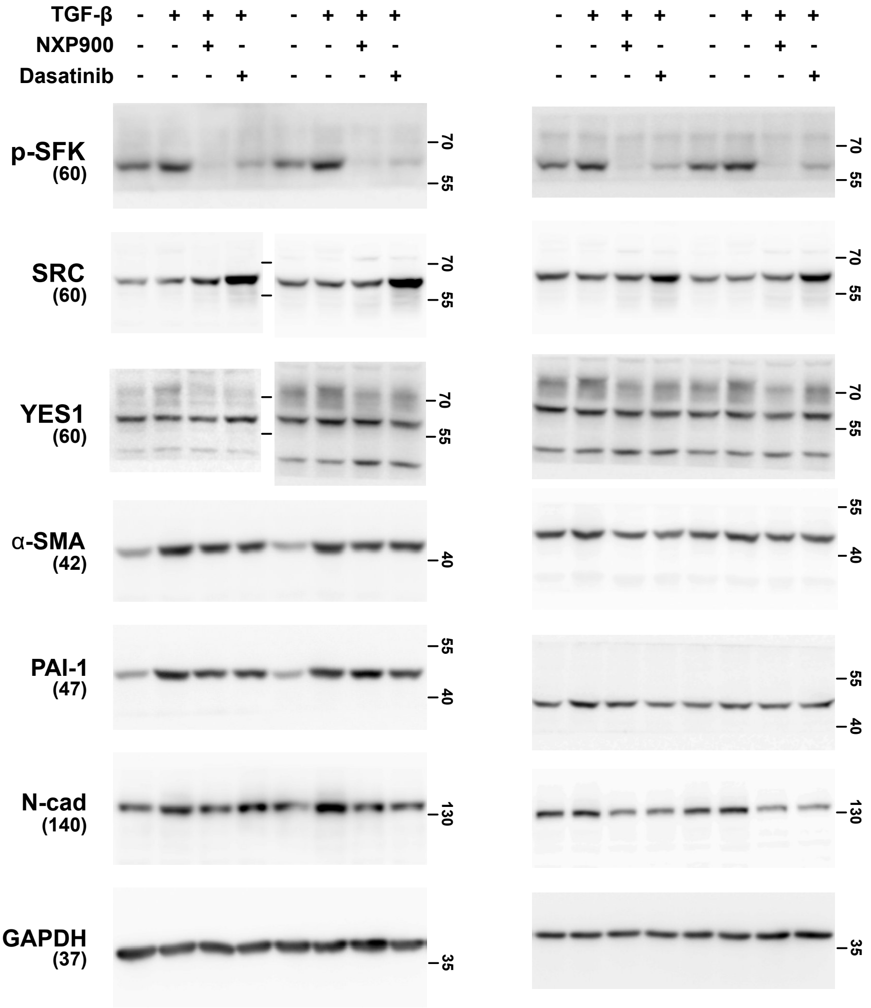

Fig. 7K

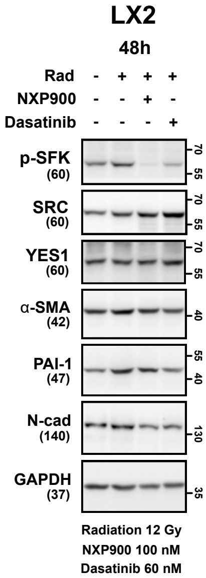

LX2
48h
Rad - + + +
NXP900 - - + -
Dasatinib - - - +
p-SFK (60)
SRC (60)
YES1 (60)
α-SMA (42)
PAI-1 (47)
N-cad (140)
GAPDH (37)
Radiation 12 Gy
NXP900 100 nM
Dasatinib 60 nM

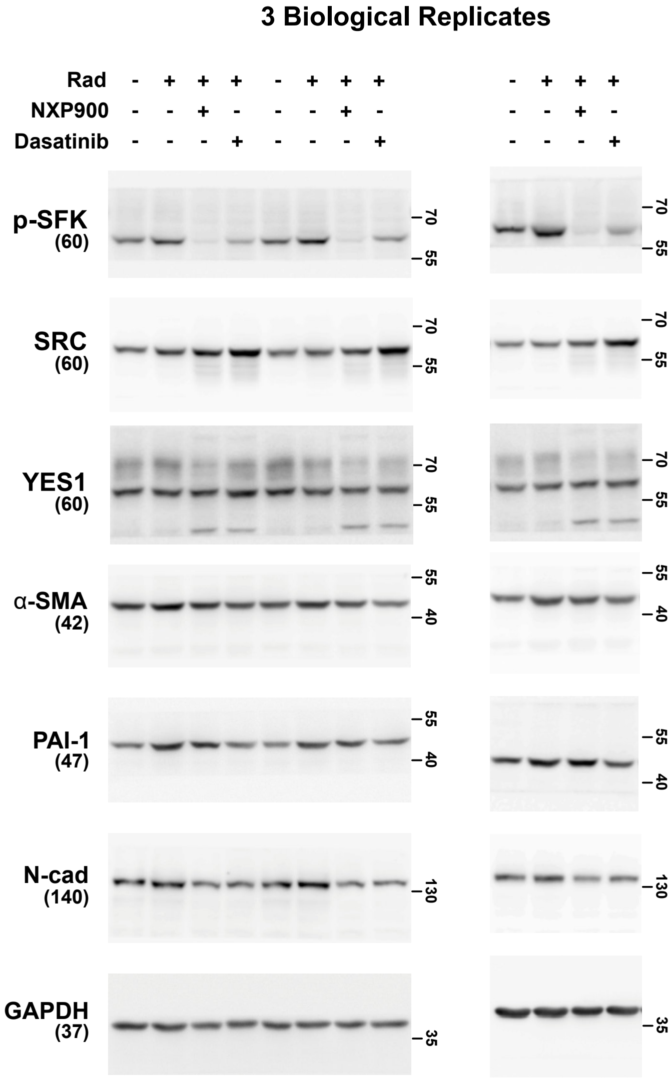

3 Biological Replicates
Rad - + + + - + + + - + + +
NXP900 - - + - - - + - - - + -
Dasatinib - - - + - - - + - - - +
p-SFK (60)
SRC (60)
YES1 (60)
α-SMA (42)
PAI-1 (47)
N-cad (140)
GAPDH (37)

Fig. 8G

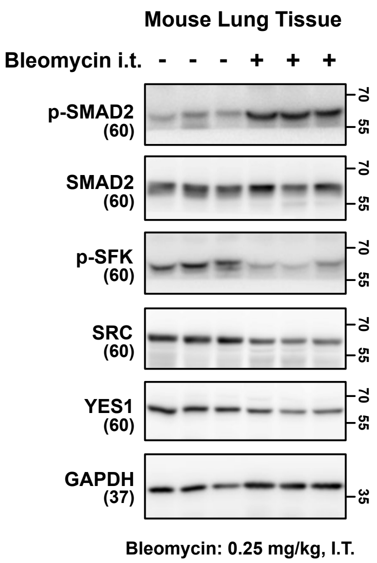
Mouse Lung Tissue
Bleomycin i.t. - - - + + +
p-SMAD2 (60)
SMAD2 (60)
p-SFK (60)
SRC (60)
YES1 (60)
GAPDH (37)
70
55
35
Bleomycin: 0.25 mg/kg, I.T.

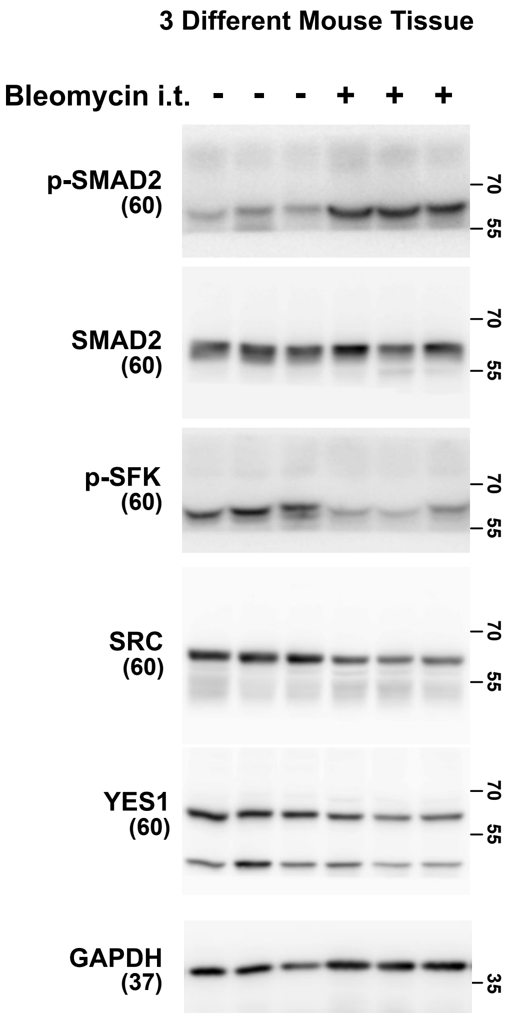
3 Different Mouse Tissue
Bleomycin i.t. - - - + + +
p-SMAD2 (60)
SMAD2 (60)
p-SFK (60)
SRC (60)
YES1 (60)
GAPDH (37)
70
55
35

Fig.8H

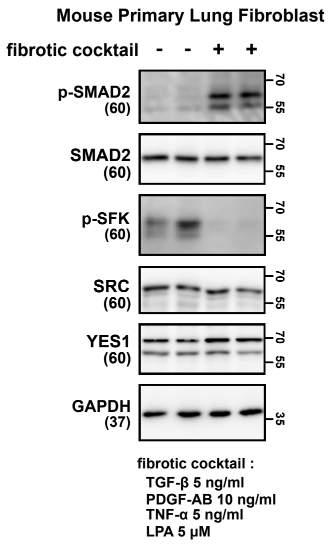
Mouse Primary Lung Fibroblast
fibrotic cocktail - - + +
p-SMAD2 (60)
SMAD2 (60)
p-SFK (60)
SRC (60)
YES1 (60)
GAPDH (37)
70
55
35
fibrotic cocktail :
TGF-β 5 ng/ml
PDGF-AB 10 ng/ml
TNF-α 5 ng/ml
LPA 5 μM

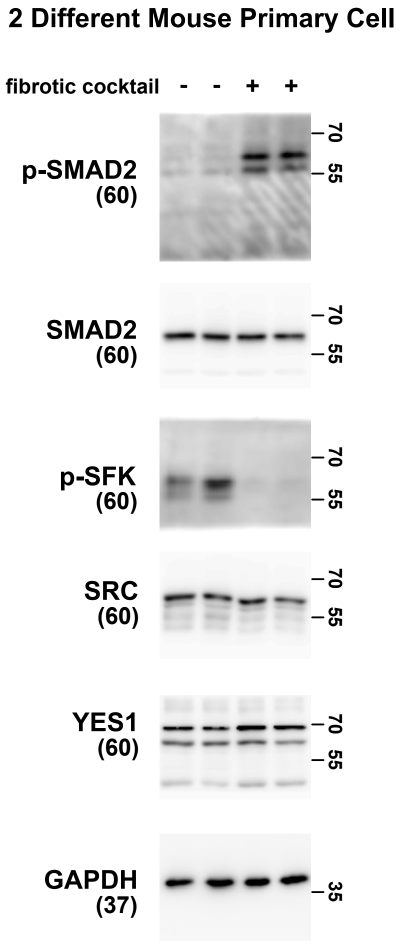
2 Different Mouse Primary Cell
fibrotic cocktail - - + +
p-SMAD2 (60)
SMAD2 (60)
p-SFK (60)
SRC (60)
YES1 (60)
GAPDH (37)
70
55
35

Supplementary Fig. S5

Control Radiation

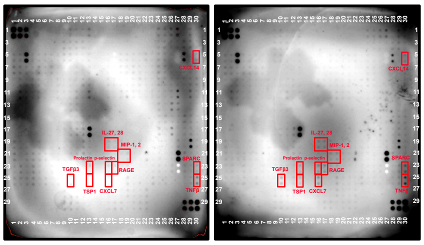

Increased by radiation

Control Radiation

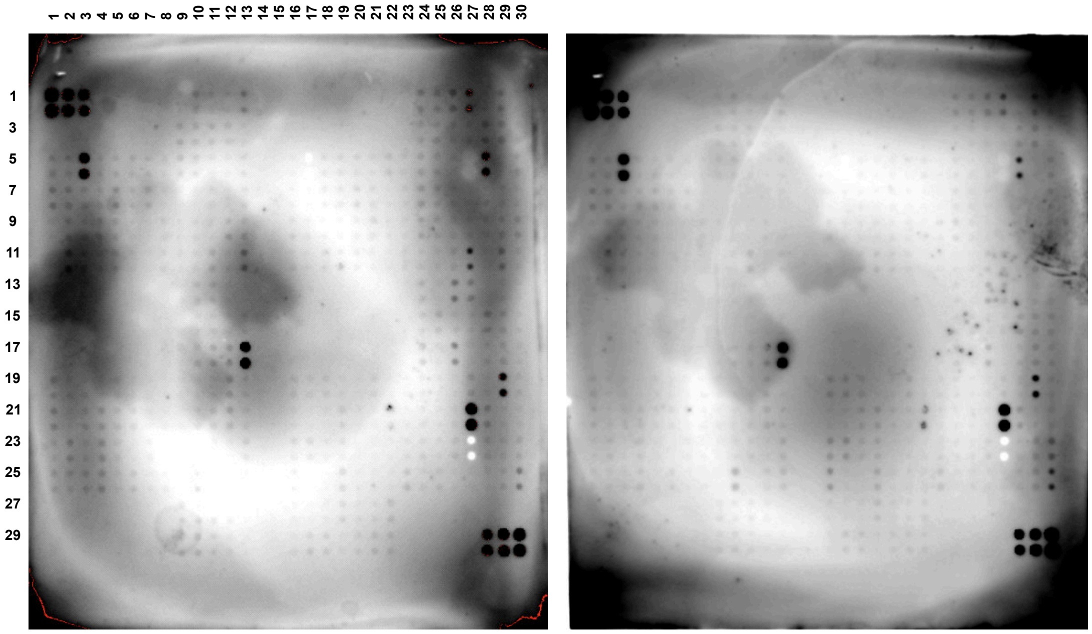

	1	2	3	4	5	6	7	8	9	10	11	12	13	14	15	16	17	18	19	20	21	22	23	24	25	26	27	28	29	30
1	POS1	POS2	POS3	Blank	Blank	Blank	Blank	Blank	1	2	3	4	5	Blank	Blank	Blank	Blank	Blank	Blank	Blank	Blank	Blank	Blank	6	7	8	9	10	11	12
2	POS1	POS2	POS3	Blank	Blank	Blank	Blank	Blank	1	2	3	4	5	Blank	Blank	Blank	Blank	Blank	Blank	Blank	Blank	Blank	Blank	6	7	8	9	10	11	12
3	Blank	Blank	Blank	Blank	Blank	Blank	Blank	Blank	13	14	15	16	17	Blank	Blank	Blank	Blank	Blank	Blank	Blank	Blank	Blank	Blank	18	19	20	21	22	23	24
4	Blank	Blank	Blank	Blank	Blank	Blank	Blank	Blank	13	14	15	16	17	Blank	Blank	Blank	Blank	Blank	Blank	Blank	Blank	Blank	Blank	18	19	20	21	22	23	24
5	25	26	27	28	29	30	31	32	33	34	35	36	37	Blank	Blank	38	39	40	41	42	43	44	45	46	47	48	49	50	51	52
6	25	26	27	28	29	30	31	32	33	34	35	36	37	Blank	Blank	38	39	40	41	42	43	44	45	46	47	48	49	50	51	52
7	53	54	55	56	57	58	59	60	61	62	63	64	65	Blank	Blank	66	67	68	69	70	71	72	73	74	75	76	77	78	79	80
8	53	54	55	56	57	58	59	60	61	62	63	64	65	Blank	Blank	66	67	68	69	70	71	72	73	74	75	76	77	78	79	80
9	81	82	83	84	85	86	87	88	89	90	91	92	93	Blank	Blank	94	95	96	97	98	99	100	101	102	103	104	105	106	107	108
10	81	82	83	84	85	86	87	88	89	90	91	92	93	Blank	Blank	94	95	96	97	98	99	100	101	102	103	104	105	106	107	108
11	109	110	111	112	113	114	115	116	117	118	119	120	121	Blank	Blank	122	123	124	125	126	127	128	129	130	131	132	133	134	135	136
12	109	110	111	112	113	114	115	116	117	118	119	120	121	Blank	Blank	122	123	124	125	126	127	128	129	130	131	132	133	134	135	136
13	Blank	Blank	Blank	Blank	Blank	Blank	Blank	Blank	137	138	139	140	141	Blank	Blank	Blank	Blank	Blank	Blank	Blank	Blank	Blank	Blank	142	143	144	145	146	147	148
14	Blank	Blank	Blank	Blank	Blank	Blank	Blank	Blank	137	138	139	140	141	Blank	Blank	Blank	Blank	Blank	Blank	Blank	Blank	Blank	Blank	142	143	144	145	146	147	148
15	Blank	Blank	Blank	Blank	Blank	Blank	Blank	Blank	149	150	151	152	153	Blank	Blank	Blank	Blank	Blank	Blank	Blank	Blank	Blank	Blank	154	155	156	157	158	159	160
16	Blank	Blank	Blank	Blank	Blank	Blank	Blank	Blank	149	150	151	152	153	Blank	Blank	Blank	Blank	Blank	Blank	Blank	Blank	Blank	Blank	154	155	156	157	158	159	160
17	Blank	Blank	Blank	Blank	Blank	Blank	Blank	Blank	161	162	163	164	165	Blank	Blank	Blank	Blank	Blank	Blank	Blank	Blank	Blank	Blank	166	167	168	169	170	171	172
18	Blank	Blank	Blank	Blank	Blank	Blank	Blank	Blank	161	162	163	164	165	Blank	Blank	Blank	Blank	Blank	Blank	Blank	Blank	Blank	Blank	166	167	168	169	170	171	172
19	173	174	175	176	177	178	179	180	181	182	183	184	185	Blank	Blank	186	187	188	189	190	191	192	193	194	195	196	197	198	199	200
20	173	174	175	176	177	178	179	180	181	182	183	184	185	Blank	Blank	186	187	188	189	190	191	192	193	194	195	196	197	198	199	200
21	201	202	203	204	205	206	207	208	209	210	211	212	213	Blank	Blank	214	215	216	217	218	219	220	221	222	223	224	225	226	227	228
22	201	202	203	204	205	206	207	208	209	210	211	212	213	Blank	Blank	214	215	216	217	218	219	220	221	222	223	224	225	226	227	228
23	229	230	231	232	233	234	235	236	237	238	239	240	241	Blank	Blank	242	243	244	245	246	247	248	249	250	251	252	253	254	255	256
24	229	230	231	232	233	234	235	236	237	238	239	240	241	Blank	Blank	242	243	244	245	246	247	248	249	250	251	252	253	254	255	256
25	257	258	259	260	261	262	263	264	265	266	267	268	269	Blank	Blank	270	271	272	273	274	275	276	277	278	279	280	281	282	283	284
26	257	258	259	260	261	262	263	264	265	266	267	268	269	Blank	Blank	270	271	272	273	274	275	276	277	278	279	280	281	282	283	284
27	Blank	Blank	Blank	Blank	Blank	Blank	Blank	Blank	285	286	287	288	289	Blank	Blank	290	291	292	293	294	295	296	Blank	Blank	Blank	Blank	Blank	Blank	Blank	Blank
28	Blank	Blank	Blank	Blank	Blank	Blank	Blank	Blank	285	286	287	288	289	Blank	Blank	290	291	292	293	294	295	296	Blank	Blank	Blank	Blank	Blank	Blank	Blank	Blank
29	Blank	Blank	Blank	Blank	Blank	Blank	Blank	Blank	297	298	299	300	301	Blank	Blank	302	303	304	305	306	307	308	Blank	Blank	Blank	Blank	Blank	POS3	POS2	POS1
30	Blank	Blank	Blank	Blank	Blank	Blank	Blank	Blank	297	298	299	300	301	Blank	Blank	302	303	304	305	306	307	308	Blank	Blank	Blank	Blank	Blank	POS3	POS2	POS1

VI. Antibody Array Target List

Number	Name	Number	Name	Number	Name	Number	Name	Number	Name
1	6Ckine	63	DPPIV	125	IGFBP-1	187	IL-28B	249	SCF R
2	Activin A	64	DR3	126	IGFBP-2	188	IL-31	250	SDF-1
3	Activin C	65	Dtk	127	IGFBP-3	189	IL-31 RA	251	SAA1
4	Activin R1B	66	EDAR	128	IGFBP-5	190	Insulin	252	Shh-N
5	Adiponectin	67	EGFR	129	IGFBP-6	191	Integrin beta-2	253	SIGIRR
6	AgRP	68	EG-VEGF	130	IGFBP-L1	192	I-TAC	254	SLPI
7	ALCAM	69	Endocan	131	IGF-1	193	GRO alpha	255	Soggy-1
8	ANGPTL2	70	Endoglin	132	IGF-2	194	Kremen-1	256	SPARC
9	ANGPTL3	71	Endostatin	133	IL-1 alpha	195	Kremen-2	257	Spinesin
10	Amphiregulin	72	Eotaxin-1	134	IL-1 beta	196	Lefty-1	258	TACI
11	Artemin	73	Eotaxin-2	135	IL-1 R4	197	Leptin R	259	TARC
12	Axl	74	Epigen	136	IL-1 R6	198	LEPTIN	260	TCA-3
13	bFGF	75	Epiregulin	137	IL-1 R9	199	LIF	261	IL-27 R alpha
14	B7-1	76	Erythropoietin	138	IL-1 R1	200	LIGHT	262	TECK
15	BAFF R	77	E-Selectin	139	IL-1 R2	201	LIX	263	TFPI
16	BCMA	78	FADD	140	IL-2	202	LRP-6	264	TGF beta 1
17	beta-Catenin	79	FAM3B	141	IL-2 R alpha	203	L-Selectin	265	TGF beta 2
18	BLC	80	Fas	142	IL-2 R beta	204	Lungkine	266	TGF beta 3
19	Betacellulin	81	Fas Ligand	143	IL-3	205	Lymphotactin	267	TGF beta R1
20	Cardiotrophin-1	82	Fc gamma RIIB	144	IL-3 R alpha	206	LTBR	268	TGF beta R2
21	IL-1ra	83	FGF R3	145	IL-3 R beta	207	MAdCAM-1	269	TSP-1
22	CCL28	84	FGF R4	146	IL-4	208	MCP-1	270	CXCL7
23	MIP-1 beta	85	FGF R5 beta	147	IL-4 R	209	MCP-5	271	Tie-2
24	MCP-3	86	FGF-21	148	IL-5	210	M-CSF	272	TIMP-1
25	MCP-2	87	Flt-3 Ligand	149	IL-5 R alpha	211	MDC	273	TIMP-2
26	CCR10	88	FLRG	150	IL-6	212	MFG-E8	274	TIMP-4
27	CCR3	89	Follistatin-like 1	151	IL-6 R	213	MFRP	275	TL1A
28	CCR4	90	Fractalkine	152	IL-7	214	MIG	276	TLR1
29	CCR6	91	Frizzled-1	153	IL-7 R alpha	215	MIP-1 alpha	277	TLR2
30	CCR7	92	Frizzled-6	154	IL-9	216	MIP-1 gamma	278	TLR3
31	CCR9	93	Frizzled-7	155	IL-9 R	217	MIP-2	279	TLR4
32	CD11b	94	Galectin-3	156	IL-10	218	MIP-3 alpha	280	TMEFF1
33	CD14	95	GCSF	157	IL-10 R alpha	219	MIP-3 beta	281	TNF RI
34	CRP	96	GDF-1	158	IL-11	220	MMP-2	282	TNF RII
35	CD27	97	GDF-3	159	IL-12 p40	221	MMP-3	283	TNF alpha
36	CD27 Ligand	98	GDF-5	160	IL-12 p70	222	MMP-9	284	TNF beta
37	CD30 Ligand	99	GDF-8	161	IL-12 R beta 1	223	MMP-12	285	Thrombopoietin
38	CD30	100	GDF-9	162	IL-13	224	MMP-14	286	TRAIL
39	CD40	101	GFR alpha-2	163	IL-13 R alpha 2	225	MMP-24	287	TRAIL R2
40	CD40 Ligand	102	GFR alpha-3	164	IL-15	226	NRG3	288	TRANCE
41	Cerberus 1	103	GFR alpha-4	165	IL-15 R alpha	227	Neurturin	289	TREM-1
42	Chordin-Like 2	104	GITR	166	IL-16	228	NGFR	290	TROY
43	F3	105	GITR Ligand	167	IL-17A	229	NOV	291	TSLP
44	IL-2 R gamma	106	Glut2	168	IL-17 RB	230	Osteoactivin	292	TSLP R
45	IP-10	107	GM-CSF	169	IL-17C	231	Osteopontin	293	TWEAK
46	Cripto-1	108	Granzyme B	170	IL-17D	232	Osteoprotegerin	294	TWEAK R
47	Crossveinless-2	109	Granzyme D	171	IL-17E	233	OX40 Ligand	295	Ubiquitin+1
48	Cryptic	110	Granzyme G	172	IL-17F	234	PDGF-C	296	uPAR
49	CSK	111	Gremlin-1	173	IL-17 RA	235	PDGF R alpha	297	Urokinase
50	CTACK	112	GHR	174	IL-17 RC	236	PDGF R beta	298	VCAM-1
51	CTLA-4	113	HGFR	175	IL-17 RD	237	Pentraxin-3	299	VE-Cadherin
52	CXCL14	114	HGF	176	IL-18 R alpha	238	PF4	300	VEGF-A
53	CXCL16	115	HVEM	177	IL-20	239	PlGF-2	301	VEGFR1
54	CXCR2	116	ICAM-1	178	IL-20 R alpha	240	Progranulin	302	VEGFR2
55	CXCR3	117	ICAM-2	179	IL-21	241	Prolactin	303	VEGFR3
56	CXCR4	118	ICAM-5	180	IL-21 R	242	P-Selectin	304	VEGF-B
57	CXCR6	119	ICK	181	IL-22	243	RAGE	305	VEGF-C
58	EGF	120	IFN-alpha/beta R1	182	IL-22BP	244	RANTES	306	VEGF-D
59	Decorin	121	IFN-alpha/beta R2	183	IL-23	245	RELM beta	307	WIF-1
60	DKK-1	122	IFN-beta	184	IL-23 R	246	Resistin	308	WISP-1
61	Dkk-3	123	IFN-gamma	185	IL-24	247	S100A10		
62	Dkk-4	124	IFN-gamma R1	186	IL-27	248	SCF		

Supplementary Fig. S6B

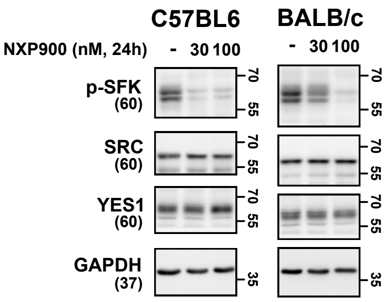

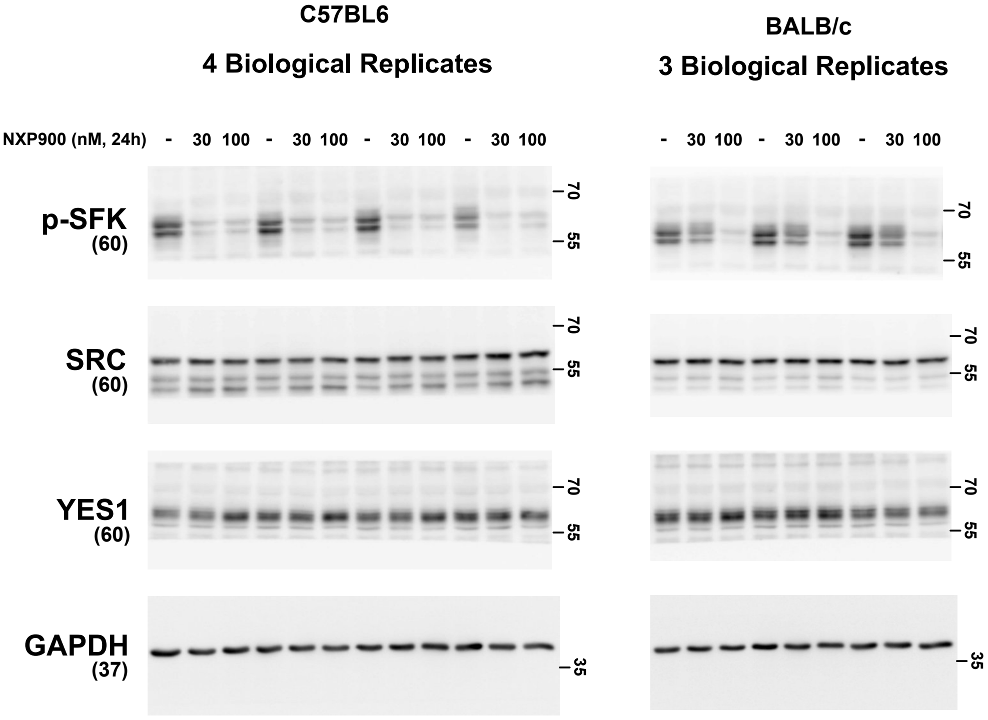

Supplementary Fig. S6G

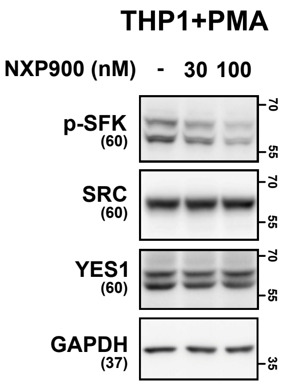

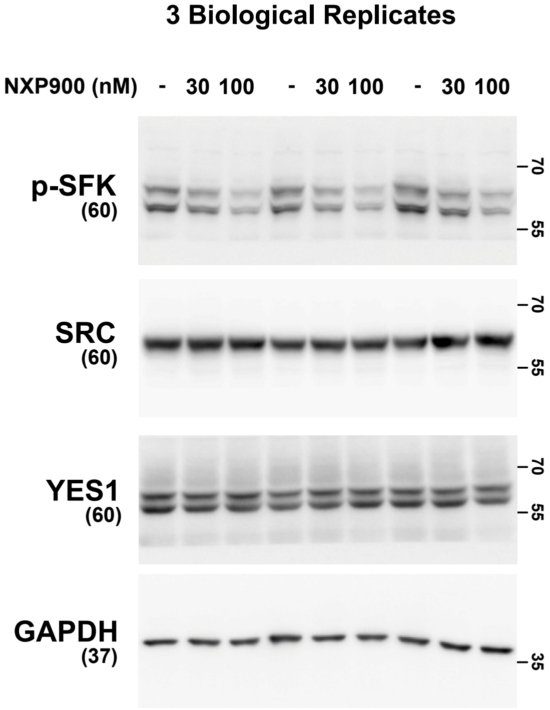

Supplementary Fig. S6J

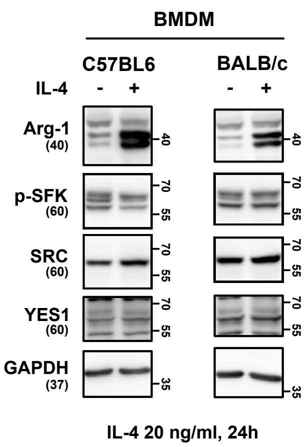

3 Biological Replicates

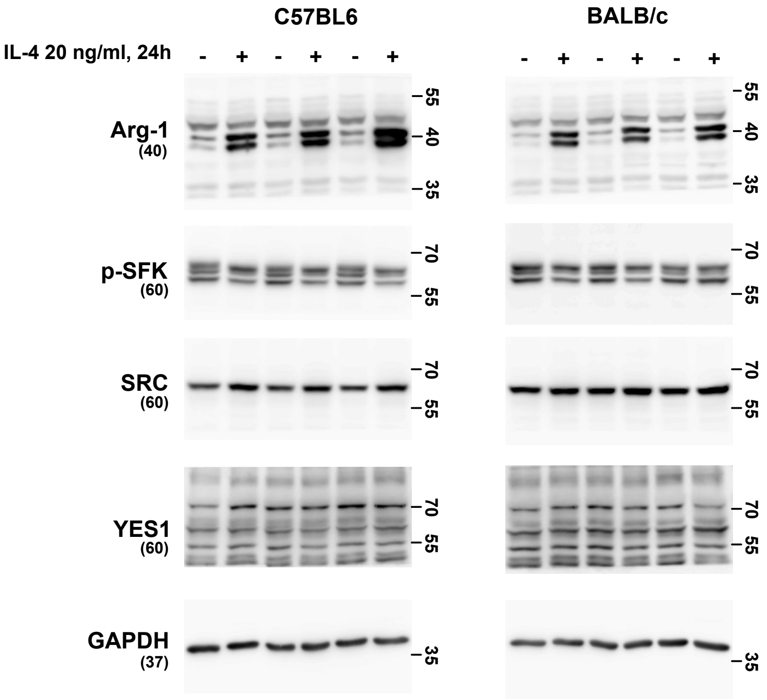

Supplementary Fig. S8E

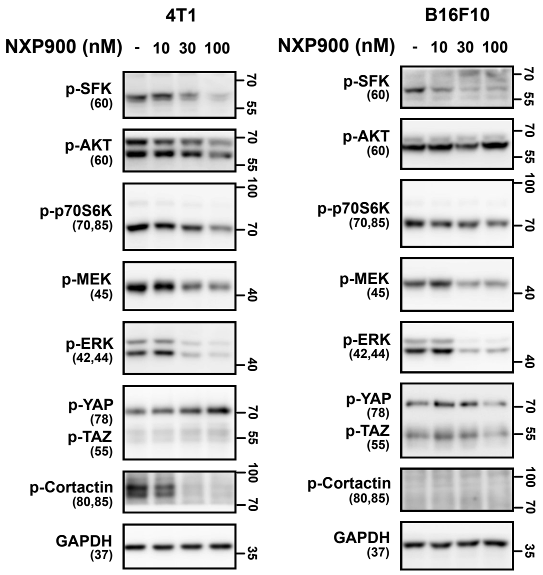

3 Biological Replicates

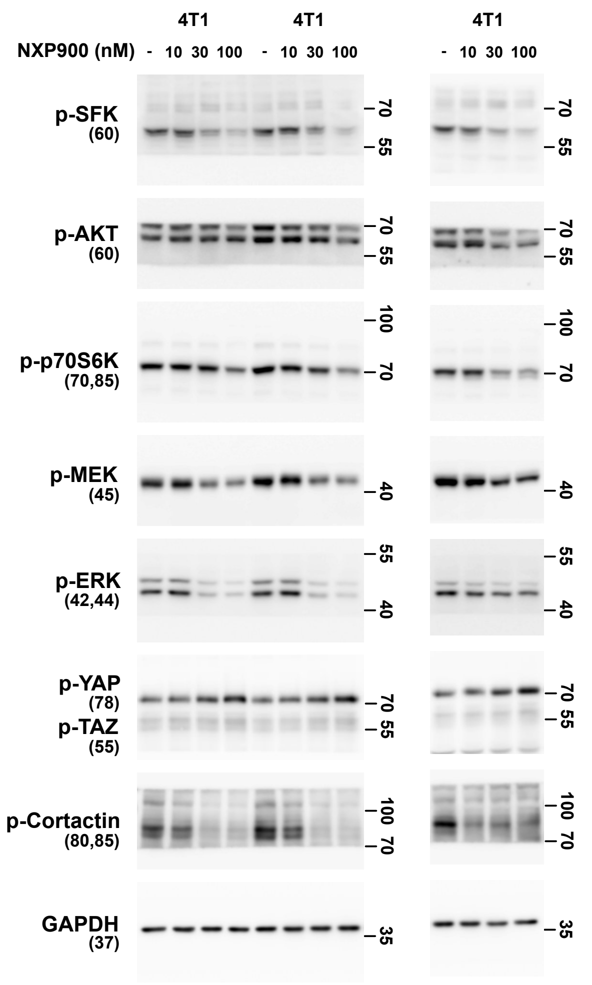

3 Biological Replicates

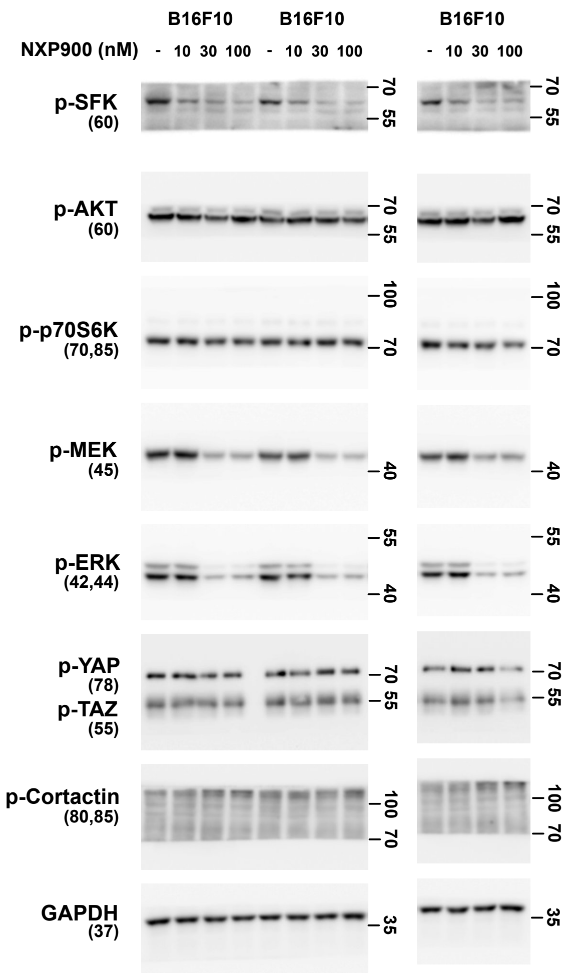

Supplementary Fig. S8F

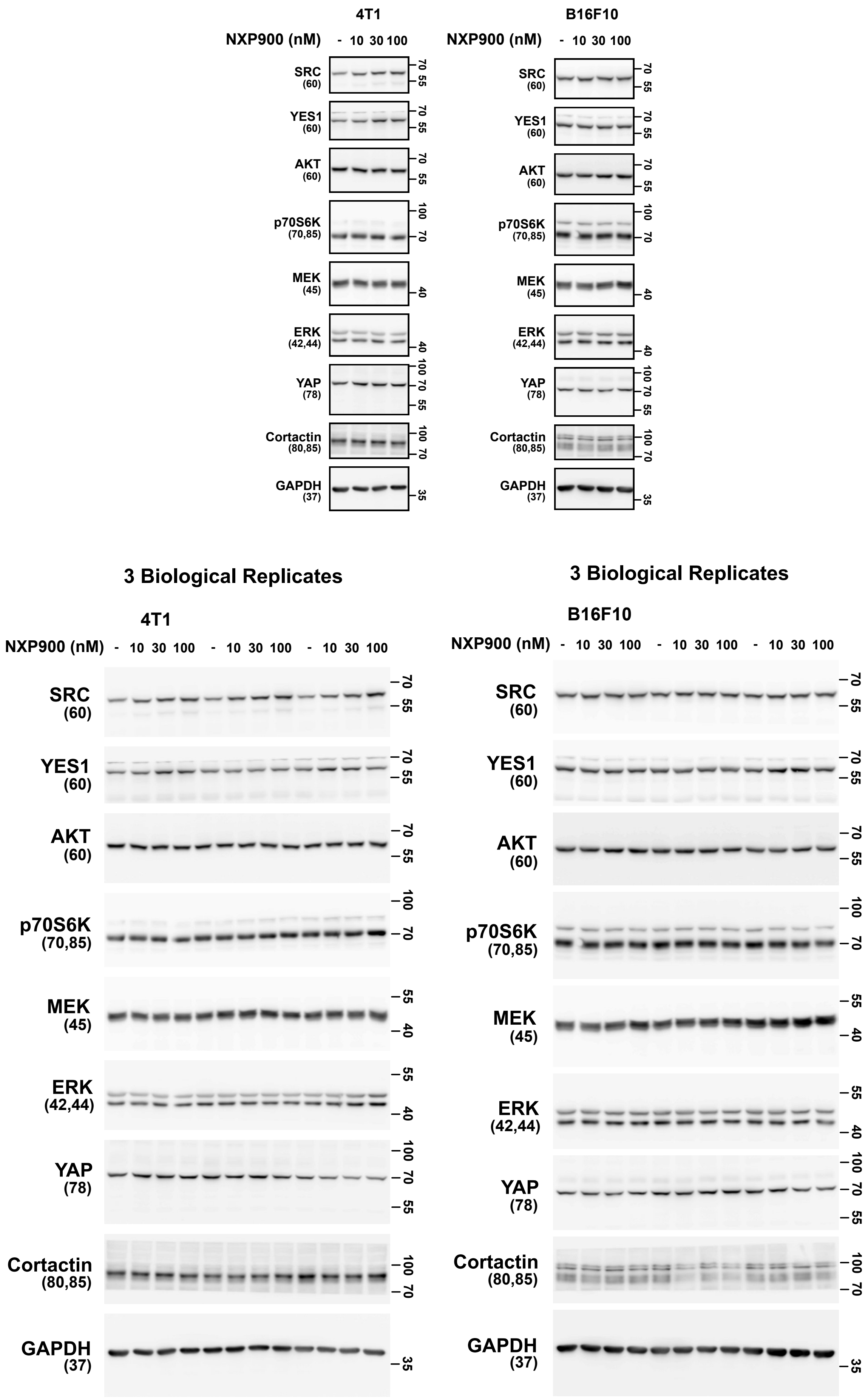

Supplementary Fig. S6I

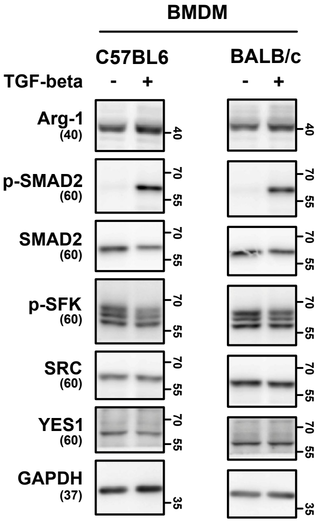

3 Biological Replicates

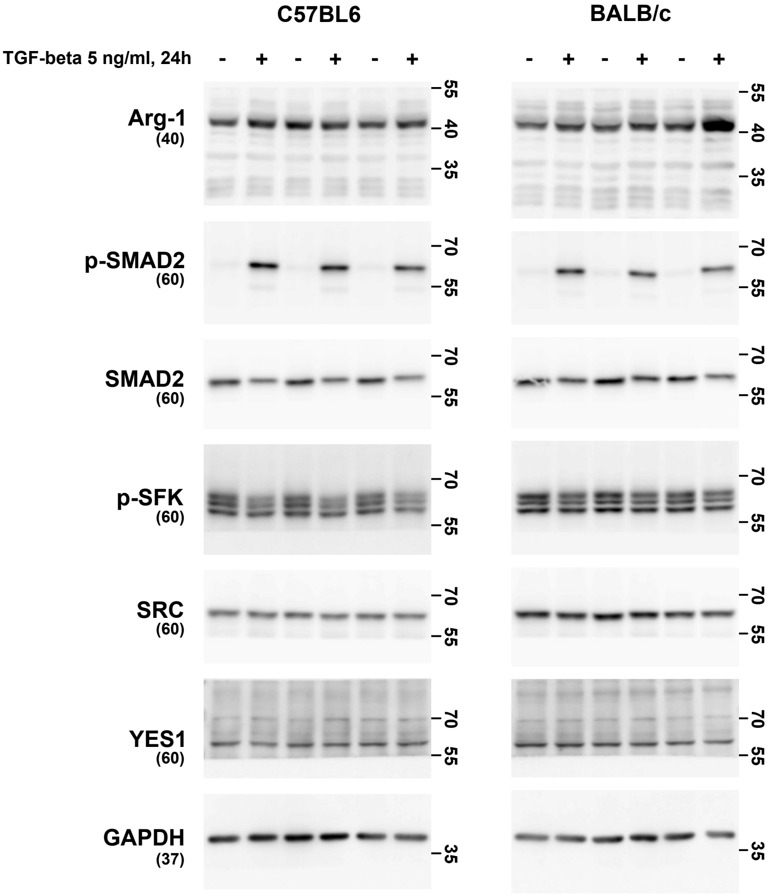

Supplement: Supplementary file 2 — Supplementary data. [file thnov15p0875s2.pdf]
